# Exploring Antimicrobial Activities of Fixed Oil From *Sanguisorba minor* Seeds by In Vitro and In Silico Analysis

**DOI:** 10.1002/open.70250

**Published:** 2026-07-02

**Authors:** Zehra Torun, Tuba Unver, Harun Uslu, Bunyamin Goktas

**Affiliations:** ^1^ Department of Pharmacognosy Inonu University, Faculty of Pharmacy Malatya Turkiye; ^2^ Department of Pharmaceutical Microbiology Inonu University, Faculty of Pharmacy Malatya Turkiye; ^3^ Department of Pharmaceutical Chemistry Fırat University, Faculty of Pharmacy Elazıg Turkiye; ^4^ Department of Pharmaceutical Chemistry Anadolu University, Graduate Education Institute Eskisehir Turkiye

**Keywords:** ADME, fatty acids, molecular docking, natural antimicrobials, *Sanguisorba minor*

## Abstract

This study aimed to demonstrate in vitro and in silico antimicrobial efficacy of the fixed oil derived from the seeds of *Sanguisorba minor* Scop., a plant traditionally utilized for wound healing, burn treatment, and hemorrhage control. As a result of GC–MS analysis, 14 distinct saturated and unsaturated fatty acids, predominantly linoleic acid (**11**, 43.25%), α‐linoleic acid (**12**, 25.33%), and oleic acid (**10**, 20.73%) were determined. The DPPH• and ABTS^+^ radical scavenging effects of the oil were determined to be of low‐middle activity. As a result of antibacterial and antifungal activities, MIC values against tested Gram‐positive and Gram‐negative species ranged between 1.406 and 11.25 µL/mL. While the MBC values of plant fixed oil for bacterial species ranged between 22.5 and 45 µL/mL, this value varied between 5.625 and 11.25 µL/mL for *Candida* species. Molecular docking studies revealed that the antifungal and antimicrobial activities of our ligands are expected to occur at micromolar levels. In particular, the antimicrobial activity of the fixed oil was demonstrated by the higher docking scores and inhibitory effects at lower concentrations of oleic acid (**10**), linoleic acid (**11**), and α‐linolenic acid (**12**), which specifically played a role in this process.

## Graphical abstract

The fixed oil from *Sanguisorba minor* Scop. seeds exhibits low antioxidant activity in DPPH• and ABTS^+^ assays, while it demonstrates high antimicrobial activity, with MBC (22.5 ‐ 45 μL/mL) and MFC (5.625 ‐ 11.25 μL/mL) values. Linoleic acid (43.26%), α‐linolenic acid (25.31%), and oleic acid (20.73%), identified by GC‐MS as the main fatty acids, are shown to be responsible for these effects through molecular docking and ADME predictions.

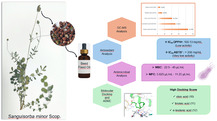

## 1 Introduction

Fixed oils, a compelling subject of inquiry in nutrition and health sciences, are composed of fatty acids that constitute the fundamental components of lipids, composed of carbon, hydrogen, and oxygen atoms, and exist in many forms, including saturated, monounsaturated, and polyunsaturated. Beyond their role in energy production, the fatty acids also play a crucial role in regulating essential cellular activities and controlling biological processes, including hormone synthesis, cellular signaling, and the preservation of cell membrane flexibility [1, 2]. Accordingly, research on the effects of dietary fatty acids has deepened our understanding of their importance for human health. The chapter authored by Yılmaz and his team summarizes the potential effects of fatty acids on cardiovascular health, diabetes and metabolic syndrome, neurodegenerative diseases, cancer, and their antimicrobial and antifungal properties [3].

The *Sanguisorba* genus, known to be part of the Rosaceae family [4], is mostly found in North America, Western Europe, and Asia [5]. It has been utilized for therapeutic purposes in humans and as a beneficial feed for animals for over 2000 years [6, 7]. The title ‘*Sanguisorba*’ is based on the Latin words “*sanguis*” (blood) and “*sorbere*” (to absorb), indicating its ability to stop bleeding [8]. In this context, the public commonly uses species from this genus to manage bleeding. Ten different species belonging to the genus *Sanguisorba* have been identified: *S. albanica* András. & Jáv., *S. annua* (Nutt. ex Hook.) Nutt. ex Torr. & A. Gray, *S. armena* Boiss., *S. canadensis* L., *S. hybrida* (L.) Nordborg, *S. menziesii* Rydb., *S. minor* Scop., *S. officinalis* Scop., *S. tenuifolia* Fisch. ex Link, and *S. verrucosa* (G.Don) Ces [9, 10].

*Sanguisorba minor* Scop., a wild edible plant [11, 12, 13], has been traditionally employed in the treatment of wounds, burns, scalds, hemorrhoids, hemorrhagic discharge, uterine hemorrhage, chronic intestinal infections, hematochezia, hemoptysis, tuberculosis, and the toxic effects of carbuncle [14], as well as in the management of rheumatism and gout [15]. It also offers various benefits, including blood sugar regulation [16], diuretic properties, and stimulation of digestion and appetite [17].

Plants belonging to the genus *Sanguisorba* are pharmacologically and medicinally valuable, and numerous studies have been conducted on the medicinal uses of species belonging to this genus [15, 18, 19]. However, studies on *S. minor* are limited in the literature. Studies performed with this species typically use extracts, essential oils, and fixed from the root, stem, or leaf parts of the plant [7, 20, 21]. When research on fixed oil content is examined, the fatty acid composition of the fixed oil derived from the aerial parts of *S. minor* [6, 20, 22] and *S. minor* ssp. *muricata* [23], and the fixed oil extracted from the seeds of *S. minor* [24] have been documented.

This study aimed to ascertain the phytochemical composition of the fixed oil derived from *S. minor* Scop. This study attempted to determine the phytochemical composition of the fixed oil extracted from *S. minor* Scop. seeds, elucidate its antioxidant and antimicrobial properties by in vitro methods, and identify the compounds potentially responsible for the antimicrobial activity using in silico molecular modeling and ADME techniques. The primary rationale for selecting this plant is its prevalence in nature, ease of accessibility, and traditional application in the treatment of wounds and burns susceptible to infection. *S. minor*, named for its hemostatic properties, will serve as the foundation for clinical studies aimed at assessing its in vitro antioxidant activity against cellular free radicals associated with these disease states, as well as its in vitro antimicrobial efficacy against bacterial and fungal infections that may arise depending on the duration of treatment. Moreover, the article's uniqueness was established by demonstrating the in vitro antimicrobial activity of the seed oil and assessing the effects of its fatty acids in silico.

## 2 Results

### 2.1 Yield, Density, and Chemical Composition of Fixed Oil

**FIGURE 1:**
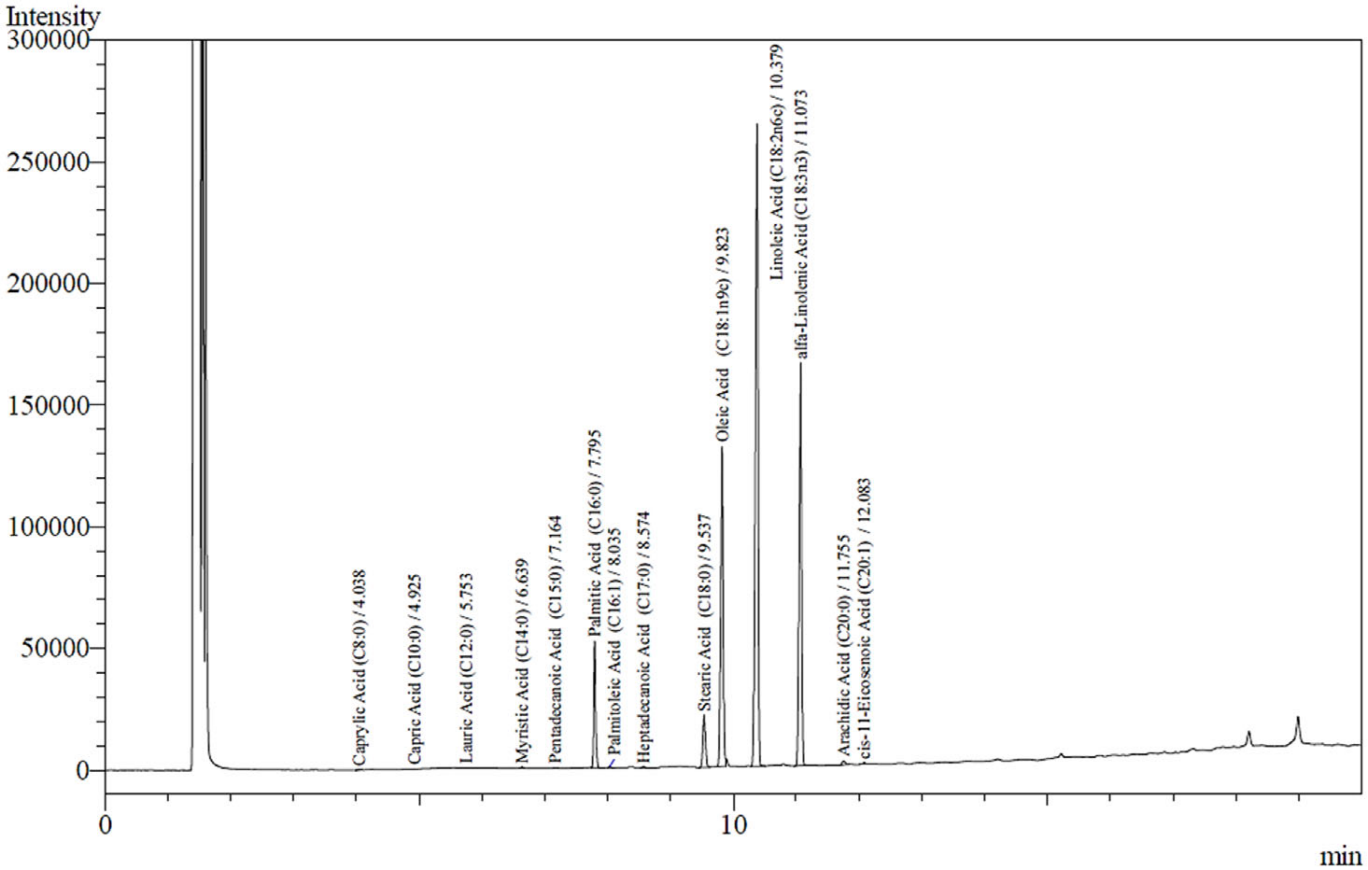
GC–MS chromatogram of phytochemical components of fixed oil from *S. minor* seeds.

As a result of continuous extraction, 1.5777 g of fixed oil was obtained from seed powder (17.9030 g). The fixed oil yield from *S. minor* Scop. seeds is 8.8% w/w. The density of the fixed oil was measured with precision scales and determined to be *p* = 0.948 g/mL (20°C). The fixed oil gas chromatography–mass spectrometry (GC–MS) chromatogram is displayed in Figure 1. Fourteen components were discovered, comprising 100% of the fixed oil. Among these, five compounds were selected as the most dominant compounds found at more than 1% v/v (Table 1). These are linoleic acid (**11**, 43.25%), α‐linolenic acid (**12**, 25.33%), oleic acid (**10**, 20.73%), palmitic acid (**6**, 6.41%), and stearic acid (**9**, 3.65%), respectively. These five compounds are believed to significantly influence the properties of the fixed oil, whereas the other nine compounds, present at concentrations below 1% v/v, could potentially contribute to the overall activity.

**TABLE 1:** Phytochemical components of fixed oil from *S. minor* seeds.

Peak/compound number	Compound	Retantion time, min	Content, %v/v
1	Caprylic acid (C8:0)	4.038	0.0098
2	Capric acid (C10:0)	4.925	0.0152
3	Lauric acid (C12:0)	5.753	0.0101
4	Myristic acid (C14:0)	6.639	0.0595
5	Pentadecanoic acid (C15:0)	7.164	0.0228
6	Palmitic acid (C16:0)	7.795	**6.4106**
7	Palmitoleic acid (C16:1)	8.035	0.0647
8	Heptadecanoic acid (C17:0)	8.574	0.0461
9	Stearic acid (C18:0)	9.537	**3.6507**
10	Oleic acid (C18:1n9c)	9.823	**20.7304**
11	Linoleic acid (C18:2n6c)	10.379	**43.2587**
12	α‐Linolenic acid (C18:3n3)	11.073	**25.3311**
13	Arachidic acid (C20:0)	11.755	0.3084
14	*cis‐*11‐Eicosenoic acid (C20:1)	12.083	0.0818
	**TOTAL**		**100.00**

*Note*: The bold values highlighted in the table represent the percentage content of the most abundant compounds in the fixed oil. These compounds are thought to be responsible for the activity.

### 2.2 Antioxidant Activity Result

The antioxidant result of the fixed oil obtained from *S. minor*, according to the DPPH• and ABTS^+^ radical scavenging, was evaluated against gallic acid (GAE), one of the natural antioxidants, using the independent sample *T*‐test. The IC_50_ values of the GAE in the DPPH• and ABTS^+^ assays were determined to be 0.0083 ± 0.0028 mg/mL and 0.0247 ± 0.0064 mg/mL, respectively. The IC_50_ values of the fixed oil in the DPPH• and ABTS^+^ assays were determined to be 100.13 ± 0.62 mg/mL and 2.75 ± 0.76 mg/mL, respectively (Table 2). GAE, employed as a calibration standard, demonstrated linearity throughout the concentration range of 0.001–2 mg/mL for the DPPH• (*y* = 0.0011x + 72 938, *R* ^2^ = 0.992) and the ABTS^+^ (*y* = 1788.6x − 0.6354, *R* ^2^ = 0.9931) assays. The IC_50_ value was reported as mean ± standard deviation (*n* = 3). The DPPH• and ABTS^+^ radical scavenging activities of the fixed oil were quantified as mg GAE/g oil. Variability among replicate % inhibition levels for each experiment was assessed using Student's *t*‐test in Excel (*p* < 0.05). The low standard deviation values signify good repeatability of the measurements for both antioxidant assays.

**TABLE 2:** Antioxidant result of the fixed oil of *S. minor*.

Assays	**IC**_**50**_ **of fixed oils (mg/mL ± standard deviation)**	Antioxidant capacity (mg GAE/g fixed oil ± standard deviation)	
DPPH•	100.13 ± 0.62^a^	0.083 ± 0.028	
ABTS^**+**^	2.75 ± 0.76^a^	8.98 ± 3.40	

*Note*: IC_50_ values of fixed oil are presented as mean ± standard deviation (*n* = 3). Antioxidant capacities of fixed oil are presented as mg of GAE per gram of fixed oil ± standard deviation (*n* = 3).

a Statistically significant differences (*p* < 0.05, *n* = 3).

### 2.3 Antibacterial and Antifungal Activity Results

**FIGURE 2:**
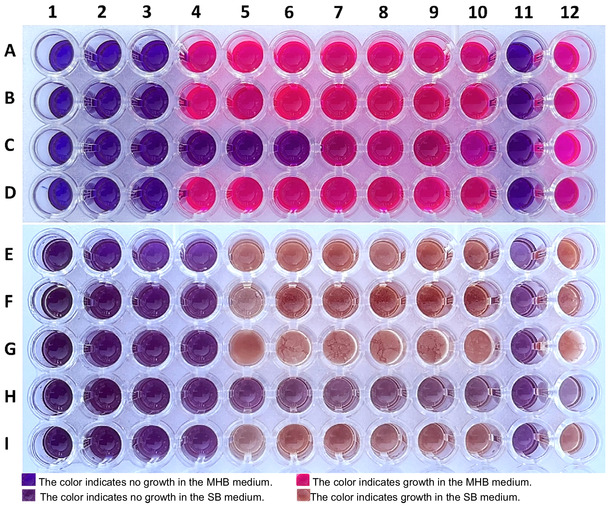
Antibacterial and antifungal activity test results of *S. minor* seed fixed oil against *E. coli* (A), *K. pneumoniae* (B), *S. aureus* (C), *E. aerogenes* (D), *C. tropicalis* (E), *C. glabrata* (F), *C. krusei* (G), *C. parapsilosis* (H), and *C. albicans* (I). The concentration of plant seed oil varies between 45 (1st well), 22.5 (2nd well), 11.25 (3rd well) (MIC for *E. coli*, *K. pneumoniae* and *E. aerogenes*), 5.625 (4th well) (MIC for *C. tropicalis*, *C. glabrata*, *C. krusei*, *C. parapsilosis* and *C. albicans*), 2.813 (5th well), 1.406 (6th well) (MIC for *S. aureus*), 0.703 (7th well), 0.352 (8th well), 0.176 (9th well), and 0.088 (10th well) µL/mL. MIC is the lowest concentration of plant seed oil at which microorganisms cannot grow, and there is no color change or visible turbidity. Eleventh and twelfth wells are the negative and positive controls, respectively (a reference drug was not used).

As a result of the antibacterial and antifungal tests, studies observed the color change in the microplate treated with resazurin, and the minimum inhibitory concentration (MIC) values were determined (Figure 2). In this study, the color change observed in wells treated with resazurin indicates the metabolic activity in those wells. Therefore, the blue‐purple color observed at the initial plant concentrations (in the first wells) changes to pink‐salmon as microorganisms grow in the wells. The 11th well is a negative control containing only liquid culture medium and indicating no contamination during the experiment. The 12th well is a positive control containing broth media and the standard microorganism inoculated at the same time, to prove the viability of the microorganism. *E. coli*, *K. pneumoniae,* and *E. aerogenes* grew from the 4th well. Therefore, the MIC value for these three bacteria was determined as 11.25 µL/mL*. S. aureus* grew from the 7th well; therefore, the MIC value in the 6th well was determined as 1.406 µL/mL. All tested *Candida* species grew from the 5th well. Therefore, the MIC value for all *Candida* species was determined as 5.625 µL/mL. Although the color difference in the wells where *C. parapsilosis* growth was observed after inoculation in the H row was not very pronounced, a comparison with the color of the positive control in the 12th well indicates that *C. parapsilosis* started growing from the 5th well onward. The lack of pronounced color difference here suggests that it may be due to a possible margin of error in manual processing.

**FIGURE 3:**
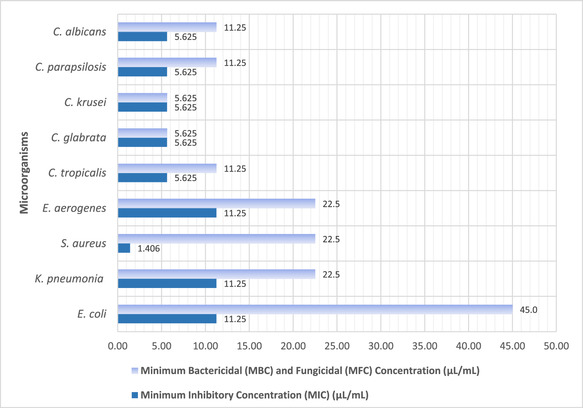
Minimum inhibitory concentrations (MICs) and minimum bactericidal (MBC) and fungicidal (MFC) concentrations of *S. minor* seed fixed oil.

Samples taken from the wells with no growth were reinoculated onto an agar medium, and minimum bactericidal concentrations (MBCs) and minimum fungicidal concentrations (MFCs) were determined (Figure 3). As a result, it was observed that *S. minor* seed fixed oil had a cidal effect on microorganisms at specific rates. The MBC value of the fixed oil against *E. coli* was determined as 45 µL/mL. Moreover, the MBC value of the fixed oil against *K. pneumoniae*, *S. aureus*, and *E. aerogenes* was determined as 22.5 µL/mL. The MFC value of the fixed oil against *C. tropicalis*, *C. parapsilosis*, and *C. albicans* was found to be 11.25 µL/mL. Finally, the lowest MFC value of the fixed oil against *C. glabrata* and *C. krusei* was determined as 5.625 µL/mL (Figure 3).

### 2.4 Molecular Docking Analysis Results

**FIGURE 4:**
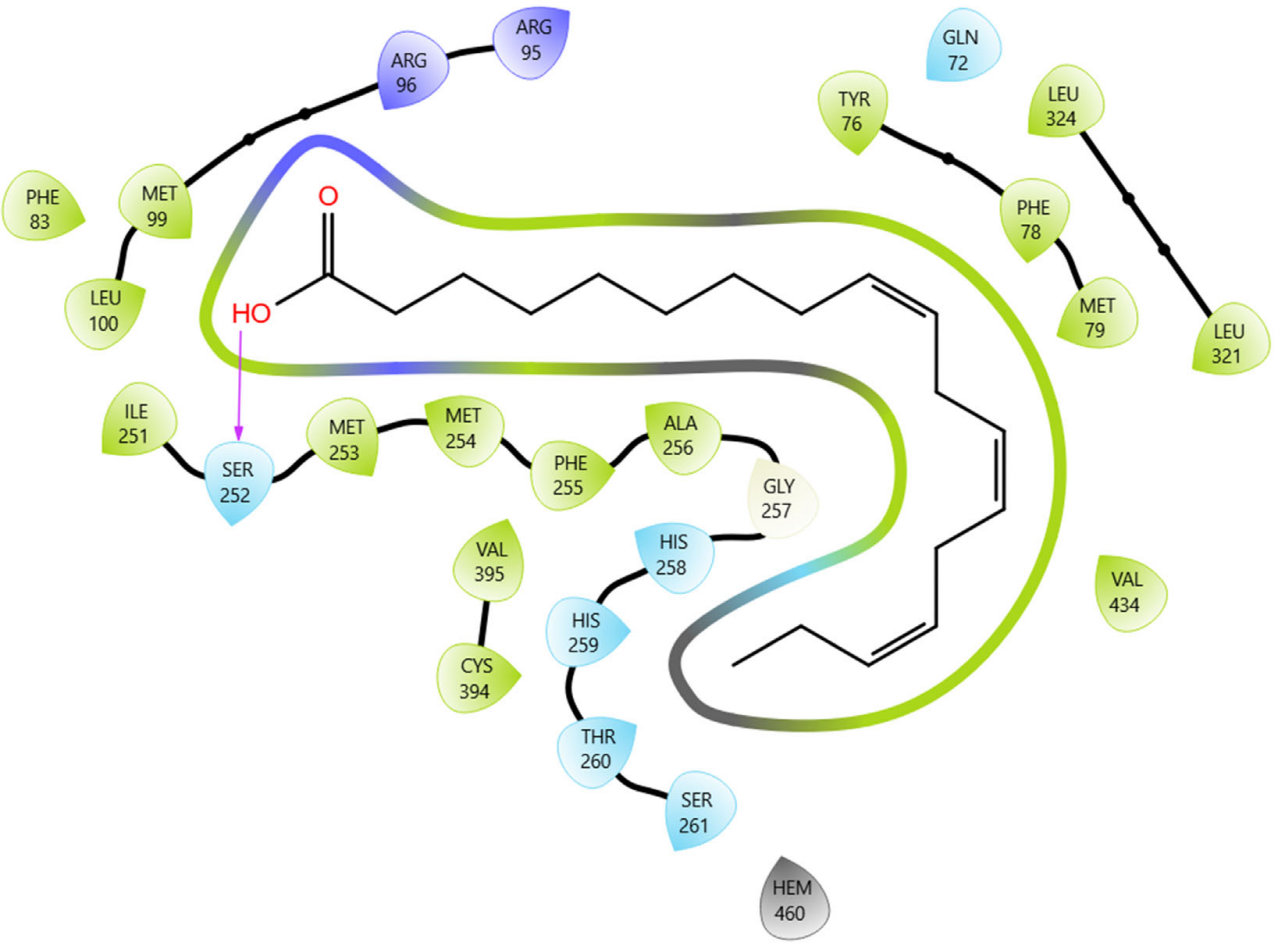
2D interaction diagram with 1EA1 for α‐linolenic acid (**12**).

**FIGURE 5:**
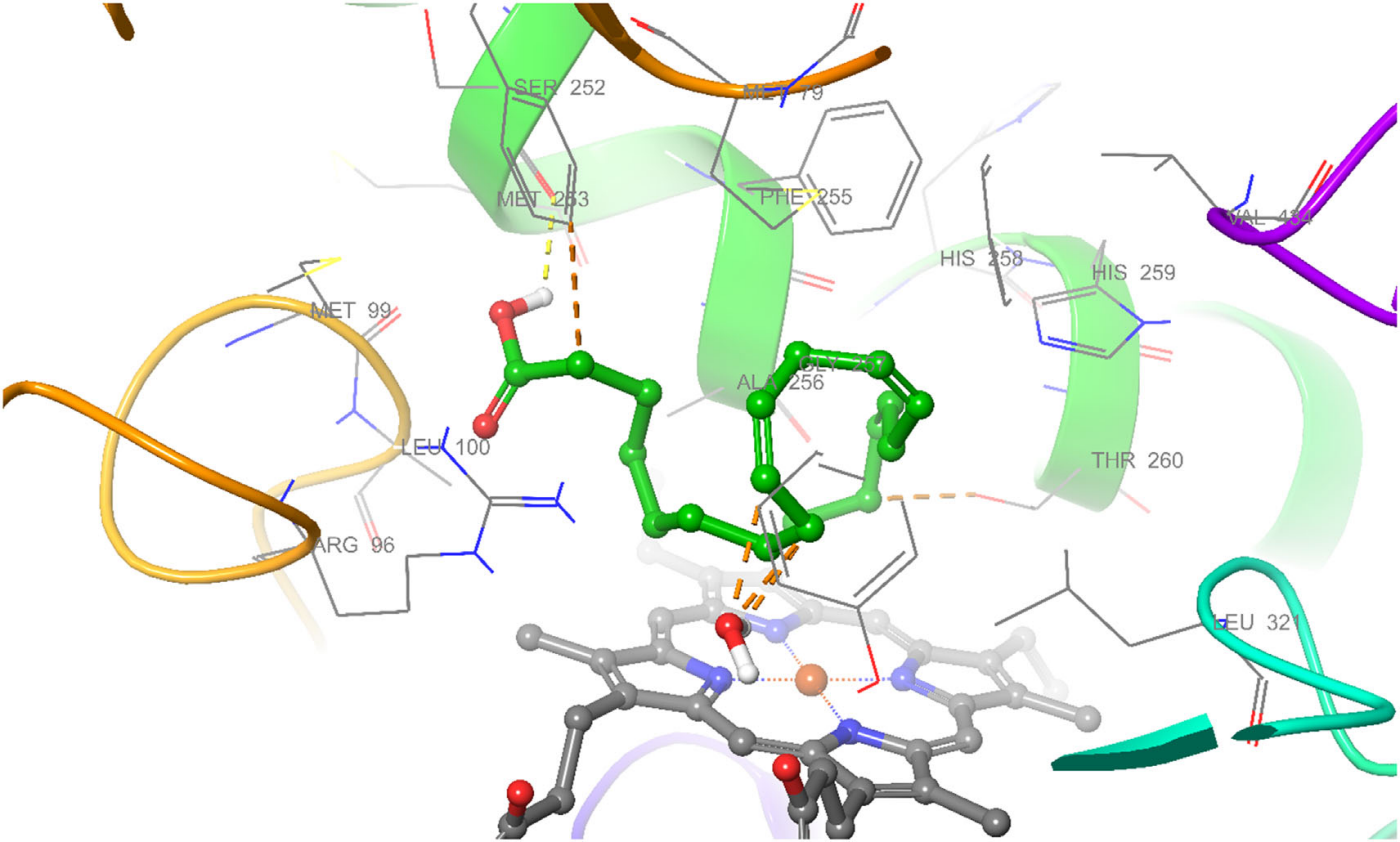
3D interaction diagram with 1EA1 for α‐linolenic acid (**12**).

The active binding sites of the receptor 1EA1 have been previously calculated or determined in the protein database [25]. In light of this data, docking studies were performed to see the interaction modes of all compounds constituting the fixed oil from *S. minor* seeds with the active site of the macromolecule. Binding types and associated residues were generated in detail by Maestro Viewer (Table 3, Figures 4 and 5). Some residues previously identified as important for the interaction between *Mycobacterium tuberculosis* and its cofactor HEM were described in detail in our previous study [26, 27]. All of our ligands except 2, 7, 10, and 12 were found to hydrogen bond with ARG96 of *M. tuberculosis* Cytochrome P450 14 alpha‐sterol demethylase (CYP51). The cocrystal ligand TPF (fluconazole) found in the crystal structure of Cytochrome P450 14 alpha‐sterol demethylase (CYP51) from *M. tuberculosis* was also found to interact with this residue in a similar manner. However, it was concluded that none of our ligands interacted with HEM460 and did not form a metal complex. The interaction modes with 1EA1 for compound **12** was visualized in 2D and 3D with the Maestro viewer (Figures 4 and 5).

**TABLE 3:** Molecular docking scores, interaction types, and estimated inhibition constants of fixed oil from *S. minor* seeds and PDB ID:1EA1.

Comp.	Autodock results			Vina results
	**Hydrogen** **bonding** **residues**	Estimated inhibition constant, Ki, µM	**Best** **docking** **score**	Best docking score
1	ARG96	806.92	−4.22	−5.4
2	—	219.64	−4.99	−5.8
3	ARG96	111.42	−5.39	−6.1
4	ARG96	41.74	−5.97	−6.5
5	ARG96	46.19	−5.91	−6.4
6	ARG96	29.59	−6.18	−6.7
7	—	37.26	−6.04	−7.0
8	ARG96	29.91	−6.17	−4.7
9	ARG96	21.70	−6.36	−6.6
10	—	18.77	−6.46	−7.1
11	ARG96	4.87	−7.25	−5.1
12	SER252	3.97	−7.37	−5.4
13	ARG96	14.81	−6.59	−7.0
14	ARG96	5.78	−7.15	−6.7

*Note*: docking score: estimated free energy of binding (kcal/mol).

Abbreviation: µM, micromolar.

**FIGURE 6:**
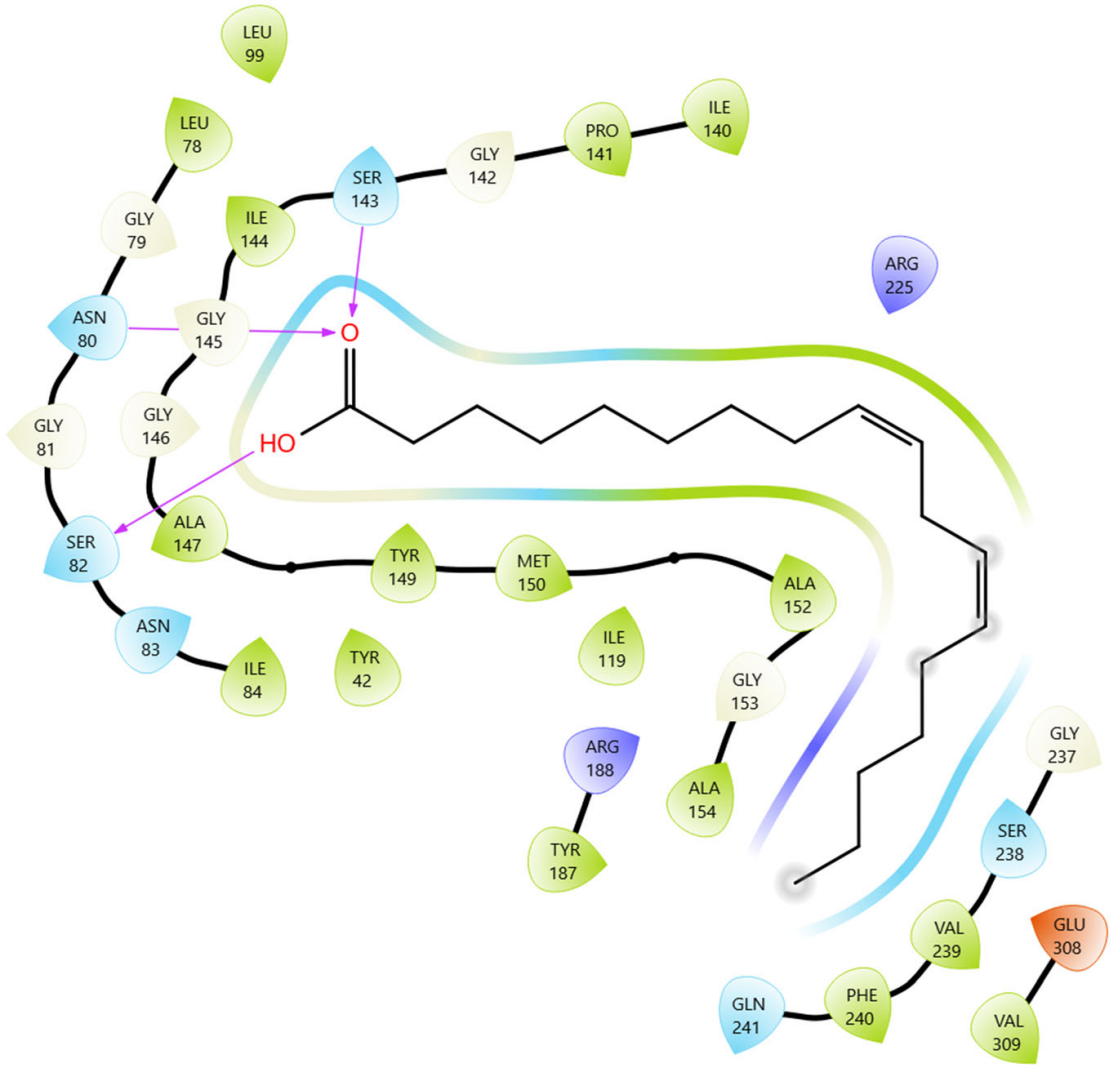
2D interaction diagram with 1HSK for linoleic acid (**11**).

**FIGURE 7:**
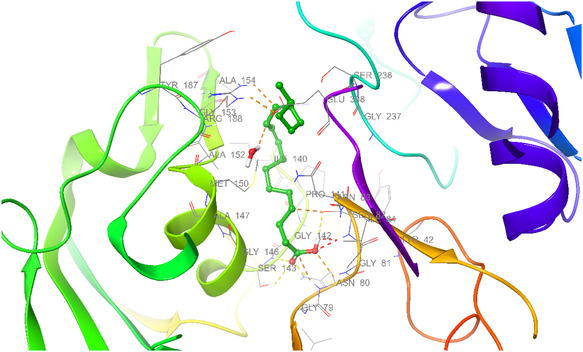
3D interaction diagram with 1HSK for linoleic acid (**11**).

The active binding sites of the receptor 1HSK have been previously calculated or determined in the protein database [28]. In light of this data, docking studies were performed to see the interaction modes of all compounds constituting the fixed oil from *S. minor seeds* with the active site of the macromolecule. Binding types and associated residues were generated in detail by Maestro Viewer (Table 4, Figures 6 and 7). Some residues previously identified as important for the interaction between *S. aureus* Murb and its cofactor FAD were described in detail in our previous study [29, 30]. Our ligands were found to form hydrogen bonds with ASN80, GLY81, SER82, SER143, GLY153, and ARG225. The cofactor FAD found in the *S. aureus* murb crystal structure was also found to interact with these residues in a similar manner. The interaction modes with 1HSK for compound **11** were visualized in 2D and 3D with the Maestro Viewer (Figures 6 and 7).

**TABLE 4:** Molecular docking scores, interaction types, and estimated inhibition constants of fixed oil from *S. minor* seeds and PDB ID:1HSK.

Comp.	Autodock results			Vina results
	**Hydrogen** **bonding** **residues**	Estimated inhibition constant, Ki, µM	**Best** **docking** **score**	Best docking score
1	ASN80 SER143	69.77	−5.67	−5.5
2	ASN80 SER82 SER143	24.99	−6.28	−5.6
3	ASN80 SER82	14.98	−6.58	−5.4
4	ASN80 SER143	9.01	−6.88	−6.7
5	ASN80 SER143	5.25	−7.20	−4.1
6	ASN80 SER143	5.31	−7.20	−7.0
7	SER82 SER143	2.13	−7.74	−7.0
8	ASN80 SER143	7.49	−6.99	−6.3
9	ASN80 SER82 SER143	12.66	−6.68	−6.3
10	ASN83	1.41	−7.98	−6.9
11	ASN80 SER82 SER143	1.05	−8.16	−5.1
12	GLY81 SER143	1.41	−7.98	−6.1
13	ASN80 SER143	4.76	−7.26	−4.6
14	GLY153 ARG225	16.30	−6.53	−6.5

*Note*: docking score: estimated free energy of binding (kcal/mol).

Abbreviation: µM, micromolar.

### 2.5 ADME Predictions Results

SMILES data of all compounds constituting the fixed oil obtained from *S. minor* seeds were obtained and used in the ADME software. When the LogS values, which are indicators of the water solubility of chemical compounds, were examined, it was seen that the solubilities of compounds numbered **1–14** were between −2.26 and −6.14. The dissolution rate class was seen to range from poorly soluble to soluble. When the cLogP results, which are the expression of the oil solubility value of the selected compounds, were examined, it was observed that the compounds showed values between 2.23 and 6.43. When the GI value, which indicates gastrointestinal absorption, was examined, it was seen that other compounds, except compounds numbered **13** and **14**, could show high absorption (Tables 5 and 6).

**TABLE 5:** Druglikeness, water solubility, and pharmacokinetic properties of fixed oil from *S. minor* seeds.

Comp.	Druglikeness					Water solubility		Pharmacokinetics	
	Lipinski	Ghose	Veber	Egan	Muegge	LogS	Class	GI abs.	***F***
1	+	−	+	+	−	−2.26	Soluble	High	0.85
2	+	+	+	+	−	−2.96	Soluble	High	0.85
3	+	+	+	+	+	−3.07	Soluble	High	0.85
4	+	+	−	+	−	−4.31	Moderatly	High	0.85
5	+	+	−	+	−	−4.66	Moderatly	High	0.85
6	+	+	−	+	−	−5.02	Moderatly	High	0.85
7	+	+	−	+	−	−4.70	Moderatly	High	0.85
8	+	−	−	−	−	−5.37	Moderatly	High	0.85
9	+	−	−	−	−	−5.73	Moderatly	High	0.55
10	+	−	−	−	−	−5.41	Moderatly	High	0.85
11	+	−	−	−	−	−5.41	Moderatly	High	0.85
12	+	−	−	−	−	−5.41	Moderatly	High	0.85
13	+	−	−	−	−	−6.44	Poorly	Low	0.85
14	+	−	−	−	−	−6.14	Poorly	Low	0.85

*Note*: LogS: ESOL, class: −6 < moderately < −4. *F*: bioavailability score.

Abbreviations: comp, compounds; GI abs, gastrointestinal absorption.

**TABLE 6:** The physicochemical and lipophilicity properties of fixed oil from *S. minor* seeds.

Comp.	Physicochemical properties							Lipophilicity
	MW	Fsp3	RB	HBA	HBD	MR	TPSA	cLogP
1	144.21	0.88	6	2	1	42.34	37.30	2.23
2	172.26	0.90	8	2	1	51.96	37.30	3.00
3	200.32	0.92	10	2	1	61.57	37.30	3.51
4	228.37	0.93	12	2	1	71.18	37.30	4.45
5	242.40	0.93	13	2	1	75.90	37.30	4.84
6	256.42	0.94	14	2	1	80.80	37.30	5.20
7	254.41	0.81	13	2	1	80.32	37.30	4.94
8	270.45	0.94	15	2	1	85.60	37.30	5.57
9	284.48	0.94	16	2	1	90.41	37.30	5.93
10	282.46	0.83	15	2	1	89.94	37.30	5.65
11	280.45	0.72	14	2	1	89.46	37.30	5.65
12	278.43	0.61	13	2	1	88.99	37.30	5.65
13	312.53	0.95	18	2	1	100.03	37.30	6.62
14	310.51	0.85	17	2	1	99.55	37.30	6.43

Abbreviations: comp, compounds; Fsp3, fraction Csp3; HBA, number of hydrogen bond acceptors; HBD, number of hydrogen bond donors; MR, molar refractivity; MW, molecular weight; RB, number of rotatable bonds; TPSA, total polar surface area.

**FIGURE 8:**
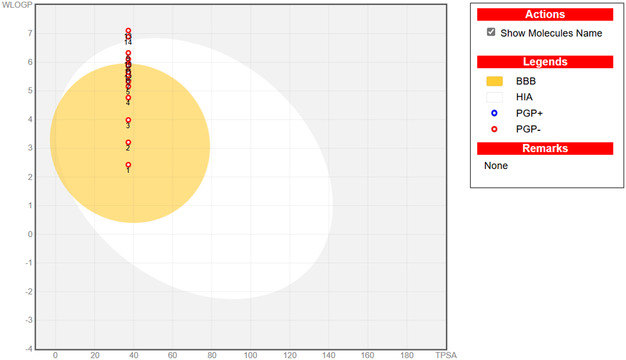
Boiled egg figure of components of fixed oil from *S. minor* seeds.

In Figure 8, when the boiled egg model showing oral bioavailability and blood–brain barrier permeability is examined, compounds numbered **13** and **14** are located outside both regions, especially because they have more than 18 carbons. The presence of compounds numbered **1–10** in the colored areas may mean they are also compatible with physicochemical parameters. Compounds numbered **11** and **12** are located in the white region, meaning that they might not pass through the blood‐brain barrier, but that their gastrointestinal absorption might be good.

## 3 Discussion

Fixed oils, advantageous for various aspects of human health, have a blend of saturated and unsaturated fatty acids. Studies on fixed oils from the *Sanguisorba* genus are limited and open to further development. Bunse et al. extracted fixed oil from the desiccated seeds of *Sanguisorba officinalis*, achieving a yield of 29% utilizing CH_2_Cl_2_. The oil's composition includes unsaturated fatty acids: 39% linolenic acid (C18:3), 36% linoleic acid (C18:2), 17% oleic acid (C18:1), and <0.5% eicosenoic acid (C20:1), alongside saturated fatty acids: 4% palmitic acid (C16:0), 2% stearic acid (C18:0), <2% arachidic acid (C20:0), and behenic acid (C22:0). The principal fatty acids are linolenic acid, linoleic acid, and oleic acid. The content study of the fixed oil derived from *S. officinalis* seeds was conducted for the first time [18]. The essential oil derived from the aerial parts of *S. officinalis* was determined to comprise 22 distinct compounds, including the unsaturated fatty acid dihomo‐γ‐linolenic acid and the saturated fatty acid undecanoic acid. The antibacterial efficacy of the extracted essential oil was assessed by measuring the inhibition zones of growth against ten distinct bacterial strains: *Sarcina lutea*, *Enterococcus faecalis*, *E. faecium*, *Escherichia coli*, *Pseudomonas fluorescens*, *P. aeruginosa*, *Streptococcus pneumoniae*, *Bacillus coagulans*, *B. subtilis*, and *B. megaterium*. The essential oil exhibited an 8–10 mm inhibitory zone against *E. coli*, *B. subtilis*, *P. aeruginosa*, and *B. megaterium* strains, while demonstrating inactivity against other bacteria. Nevertheless, these studies suggest that the antibacterial activity is derived from phenolic acids rather than fatty acids [19]. In another study, the anti‐inflammatory properties of dichloromethane extract derived from various solvent fractions of *S. tenuifolia* roots have been established, and the fatty acid α‐hydroxytetracosanic acid has been identified [31].

Upon reviewing the studies completed on *S. minor* to date, limited studies were identified as pertaining to fixed oil. Fixed oil was extracted from the aerial parts of *S. minor* ssp. *muricata*, yielding 5.9%. Palmitic acid comprises 29% of the total fatty acids, followed by linoleic acid (22.6%), linolenic acid (21.4%), and oleic acid (12.2%) [23]. The research by Elgersma et al. examined the total quantity and kinds of fatty acids in *S. minor* [6]. Karkanis et al. examined the fatty acid composition of fixed oils derived from the root and aerial parts of the plant. The results indicate substantial variations in fatty acid contents derived from the aerial parts and roots. Specifically, α‐linolenic acid (49.4%−52.4%) was prevalent in the aerial parts of the plant, followed by palmitic acid (14.6%−15.6%) and linoleic acid (12.9%−13.1%). Stearic, tricosylic, lauric, and eicosatrienoic acids were identified in lesser quantities. Conversely, roots exhibited elevated levels of linoleic acid (20.7%−23.8%) and tricosylic acid (20.5%−24.1%), followed by α‐linolenic acid (12.8%−15.4%) and palmitic acid (11.9%−13.1%). Stearic, oleic, dihomo‐γ‐linolenic, and behenic acids were identified in lesser quantities [20]. In the study conducted by Ceccanti et al., edible aerial parts of *S. minor*, which is growing both from its natural enviroment and cultivated on a local farm, were collected, dried, and afterward evaluated by nutritional composition analysis. In this comparative analysis, lauric, pentadecanoic, palmitic, stearic, and lignoceric acids were observed to be more abundant in the naturally cultivated species, whereas oleic and linoleic acids were found to be higher in the farm‐grown species. However, the major fatty acid in the aerial part of the species is found to be *α*‐linolenic acid (36.28%–37.50%), followed by palmitic acid (25.06%–22.03%) and linoleic acid (11.33%–13.40%) [22]. In addition to these studies, the first study on fatty acids in *S. minor* seeds was conducted by Kaplan et al. The fixed oil yields of seeds from 20 distinct genotypes ranged from 8.85% to 15.66%. The main components of the obtained fixed oils are palmitic acid (4.55%–10.40%) from saturated fatty acids, while oleic acid (19.57%–34.34%), linoleic acid (30.51%–49.84%) and linolenic acid (26.06%–39.76%) are unsaturated fatty acids [24].

The results of this study indicate that the fixed oil extracted from *S. minor* seeds, with a specific gravity of 0.948 g/mL and a yield of 8.8% w/w, has a total of 14 distinct saturated and unsaturated fatty acids, of which 5 are predominant. These compounds are linoleic acid (**11**, 43.25%), α‐linolenic acid (**12**, 25.33%), oleic acid (**10**, 20.73%), palmitic acid (**6**, 6.41%), and stearic acid (**9**, 3.65%), respectively, and are predicted to be responsible for the effectiveness of the fixed oil. When compared with previous study data, fixed oil yield and major compound contents are mutually supportive. A study on linoleic acid, indicated that it had no DPPH• radical scavenging action [32]. The α‐linoleic acid has been documented to demonstrate antioxidant properties by diminishing lipid peroxidation and restoring antioxidant enzymes, including superoxide dismutase (SOD), glutathione peroxidase (GPx), and catalase [33]. Oleic acid's antioxidant activity considerably mitigated oxidative stress by decreasing lipid peroxidation and inhibiting SOD activity, while also exhibiting a potent DPPH• radical scavenging effect against cadmium and cadmium‐induced oxidative stress in rats [34]. The antioxidant activity of palmitic acid compound isolated from the *Syzygium litoralle* was found with an IC_50_ value of 189.9 μg/mL [35]. Although there is no research exclusively on stearic acid, it has been shown that it protects cortical neurons from oxidative stress by enhancing internal antioxidant enzymes [36]. While these fatty acids have demonstrated antioxidant action in specific studies, assessing this property in the fixed oil in which they were identified in the current study has proven challenging.

To evaluate the antioxidant activity of fixed oils, highly toxic solvents such as ethyl acetate [37] and toluene [38], as well as solvents considered safe, such as DMSO, methanol, distilled water, and buffer solutions, are typically used to assess their antioxidant effects. In our study, DMSO was preferred as a solvent for fixed oil, while methanol was used for GAE. According to the data obtained, the radical scavenging effects of fixed oil on DPPH• and ABTS^+^ were determined as 0.083 ± 0.028 and 8.98 ± 3.40 mg GAE/g fixed oil, respectively. The difference between the values is due to the fact that DPPH• is more sensitive in hydrophilic systems, while ABTS^+^ is sensitive to antioxidants in both hydrophilic and lipophilic structures. In conclusion, although fixed oil showed a higher antioxidant capacity in the ABTS^+^ analysis, it had a low‐middle effect against GAE.

The five most abundant compounds detected in plant seed fixed oil were linoleic acid, α‐linolenic acid, oleic acid, palmitic acid, and stearic acid, respectively. These compounds have significant antimicrobial effects, as demonstrated in the literature [39, 40, 41]. According to the antimicrobial activity test results of plant seed fixed oil, the tested *Candida* species (MICs ranged between 5.625 and 11.25 µL/mL) were slightly more sensitive to the plant oil than the bacterial species (MICs ranged between 22.5 and 45 µL/mL). The differences in results are attributed to variations in the cell wall structures of the microorganisms. Therefore, the predominant antimicrobial activity of plant seed fixed oil can be attributed to these compounds.

Fatty acids, such as linoleic acid and oleic acid, generally affect the cell membranes of microorganisms. Consequently, they cause effects such as disruption of the electron transport chain, uncoupling of oxidative phosphorylation, and inhibition of enzymatic activity and nutrient uptake [40, 42, 43]. Consequently, they disrupt cell membrane permeability, leading to cell lysis. Furthermore, they may exhibit antimicrobial effects by inhibiting DNA/RNA replication in microorganisms [39, 44]. The antifungal mechanism of action of fatty acids against yeast cells generally involves disrupting cell membrane integrity by affecting the lipid bilayer of yeast cell membranes. Thus, fatty acids cause the uncontrolled release of intracellular electrolytes and proteins, ultimately leading to the lysis of fungal cells [45, 46]. In our previous study, plant extracts containing linoleic acid, oleic acid, palmitic acid, and stearic acid demonstrated potent antimicrobial activity against the tested bacteria and yeast species [41]. A study using fatty acids found that long‐ and medium‐chain fatty acids, such as linoleic acid, were more effective against oral microorganisms than short‐chain fatty acids, inhibiting the tested bacteria and *Candida* by 50%–60% [47]. Oleic and linoleic acid can increase the membrane permeability of microorganisms and have been found to cause lysis of *Streptococcus faecalis* cells [48]. In another study, electron microscopy revealed that linoleic acid induces the lysis of *Helicobacter pylori* cells [49].

A study determined the bactericidal activity of linoleic acid and α‐linolenic acid obtained from Indonesian fermented soybeans against *Staphylococcus aureus* and *Bacillus subtilis* [50]. Another study tested the antibacterial activity of linoleic and oleic acids isolated from *Helichrysum pedunculatum* leaves. Linoleic acid inhibited the growth of the tested Gram‐positive bacteria (MICs ranged from 0.01 to 1.0 mg/mL), and oleic acid inhibited three of the tested Gram‐positive bacteria (MIC: 1.0 mg/mL) [51]. McGaw and colleagues reported that linoleic acids isolated from *Schotia brachypetala* roots exhibited antibacterial activity against *Bacillus* and *Clostridium* species [52]. Ivanova and colleagues demonstrated the bactericidal activity of palmitic and stearic acid crystals against *Pseudomonas aeruginosa* and *Staphylococcus aureus*, as visualized using atomic force microscopy (AFM) and scanning electron microscopy (SEM) images [53]. Considering all these data, the predominant content of Linoleic acid, oleic acid, palmitic acid, and stearic acid in the plant in this study can be significantly associated with the antimicrobial test results found.

Molecular docking studies revealed that all ligands interacted in the binding sites for both 1EA1 and 1HSK. However, no interaction was observed with the HEM residue, which is important for 1EA1. Therefore, when the results of both macromolecules were compared, it was seen that our ligands may be more effective on 1HSK than on 1EA1. When the results of in silico and in vitro experiments are evaluated together, it can be said that especially oleic acid (**10**), linoleic acid (**11**), and α‐linolenic acid (**12**) will show antibacterial properties. The ADME profiles of compounds 1–12 with a maximum of 18 carbons were found to be good, but compounds arachidic acid (**13**) and c*is*‐11‐eicosenoic acid (**14**) were found to have poor pharmacokinetic properties.

## 4 Conclusion

Natural fixed oils can provide health benefits by diminishing infection risk via antimicrobial effects. Therefore, there is a need for natural sources rich in fatty acids with proven activity in this area. The present study demonstrates such benefits through in vitro and in silico analyses of fixed oil obtained from *S. minor* Scop seeds. The fixed oil is distinguished by its 5 main components, which include 14 distinct saturated and unsaturated fatty acids. It has a low‐middle antioxidant potential due to its unsaturated fatty acids. The plant seeds’ fixed oil exhibited strong antimicrobial properties due to the fatty acids it contained. This property was demonstrated by similar MIC values, without any specific selectivity between bacterial and yeast species. Molecular docking studies showed that when the in silico experimental results are evaluated together, it can be said that especially oleic acid (**10**), linoleic acid (**11**), and α‐linolenic acid (**12**) may have better antibacterial properties compared to antifungal. These results demonstrate that the fixed oil derived from *S. minor* seed is a promising candidate for preliminary in vitro and in silico studies, which will guide future research regarding its pharmaceutical and medicinal uses in both laboratory and clinical settings.

## 5 Experimental Section

### 5.1 Plant Material and Fixed Oil Extraction

*S. minor* Scop. were grown at Düziçi, Osmaniye (37.2416607185802, 36.45139292053739) by Müzeyyen Torun. The plant seeds were harvested by Dr. Torun following the flowering phase, during June and July 2021. The specimen is housed in the Herbarium of Inonu University Faculty of Pharmacy under voucher number ZT101. The seeds were desiccated in the shade at ambient temperature. 17.9030 g of seed powder was extracted with 300 mL n‐hexane (boiling at 69°C) in a Soxhlet apparatus for 8 h. The extracts were concentrated with the aid of a rotary evaporator at low pressure (200 mbar) and 40°C to obtain a fixed oil. Yield calculation was performed by weighing the fixed oil obtained from the powdered plant at room temperature using a precision balance after it had been separated from the solvent [54, 55].

### 5.2 Density Calculation

The density of the fixed oil was measured using the relative density model. In this model, the density of a given volume of oil at 20°C is the ratio of the mass of water at the same temperature and volume. A 25 mL pycnometer filled with distilled water at 20°C was weighed (W1). The same pycnometer was weighed again after 2 mL of distilled water was removed using a micropipette (W2). Finally, 2 mL of fixed oil was added to replace the missing 2 mL, and the pycnometer was weighed again (W3). The density of the fixed oil was determined using the formula (W3‐W2)/(W1‐W2), based on the density of distilled water, which is 1 g/mL [56].

### 5.3 Chemical Composition by GC‐FID Analysis

The chemical constituents of fixed oil from *S. minor* Scop. according to the standard containing 37 components were analyzed after esterification by GC‐FID at Atatürk University Eastern Anatolia High Technology Application and Research Centre (DAYTAM). GC–MS analysis was performed using a Shimadzu QP‐2010 Ultra (Ultra‐fast model, Japanese) gas chromatograph equipped with a flame ionization detector (FID) and an Agilent GC Capillary Column (DB‐FastFAME GC column, 30 m, 0.25 mm ID, 0.25 µm). The oven temperature was held constant at 50°C for 0.50 min and then programed at a rate of 30°C/min to 194°C and kept constant at this temperature for 3.5 min and then programed at a rate of 5°C/min to 240°C for 2 min. The column oven temperature initially was 50°C. The carrier gas was helium with a flow rate of 1 ml/min, with a linear velocity of 39.0 cm/s, a split ratio of 50. Total program time is 20 min, and the total ion current ranged from 40 to 400 m/z. Single injection performed. The injection volume was 1 µL, the injector temperature was 250°C, and the detector temperature was 260°C. The percentages of compounds were measured using the area normalization method without considering response factors [57].

The constituents of the fixed oil were determined by calculating their retention indices and comparing the associated mass spectra with those archived in a computer database. Relative area percentages obtained from FID were employed for quantification without adjustment factors [57].

### 5.4 Determination of Antioxidant Activity

#### 5.4.1 DPPH• Radical Scavenging Assay

Dilutions ranging from 40,000 to 78.125 μg/mL were made in dimethyl sulfoxide (DMSO) using the fixed oil of the plant. A 50 μL sample from each dilution was dispensed into the wells of a 96‐well plate. Each dilution received 150 μL of 0.1 mM DPPH• solution. GAE served as a reference chemical (1–0.001 mg/mL). DMSO was used as a control for the fixed oil, whereas MeOH was employed for GAE. The plate was incubated for 30 min at 37°C in the absence of light. Absorbance was then quantified at 517 nm. The experiment was performed in triplicate [58]. The values are presented as mean ± standard deviation (*n* = 3). The IC_50_ values were determined by formulating a regression equation derived from the percentage inhibition based on Formula (1). The antioxidant capacity of the fixed oil was measured as mg GAE/g sample based on this IC_50_ value in Formula (2).

### 5.5 ABTS^+^ Radical Scavenging Assay

The method was applied with modifications [59]. Using distilled water, a 2.45 mM potassium persulfate solution was formulated as stock 1, and a 7 mM ABTS^+^ solution was produced as stock 2. The prepared solutions were combined in a 1:1 ratio and thereafter incubated at ambient temperature in the absence of light for 12–16 h. The ABTS^+^ solution was diluted with DMSO at a 2:9 ratio. A 200 μL (0.1754 g) of fixed oil was dispersed in 800 μL of DMSO to prepare the sample stock solution. The fixed oil, with an initial concentration of 175.4 mg/mL, was diluted at a 50% ratio and distributed into 96‐well plates. In each well, there were 100 μL of sample and 100 μL of ABTS + solution. GAE, prepared in MeOH, was used as the reference compound, diluted at a 50% ratio at concentrations ranging from 1 to 0.001 mg/mL. Due to the significant antioxidant activity of GAE, the loading was performed using 20 μL of GAE and 200 μL of ABTS^+^ solution. DMSO served as a control for the fixed oil, whereas MeOH was utilized for GAE. Absorbance was measured at 734 nm 10 min after application. The experiment was conducted in triplicate. Values are expressed as mean ± standard deviation (*n* = 3). IC_50_ values were established by developing a regression equation based on percentage inhibition according to Formula (1). The antioxidant ability of the fixed oil was quantified in mg GAE/g per sample according to the IC_50_ value in Formula (2)



(1)

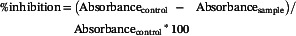






(2)

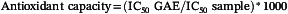




### 5.6 Antibacterial and Antifungal Activity Tests

#### 5.6.1 Strains and Media

In antibacterial activity tests, Muller–Hinton Agar (MHA) (Merk, Darmstadt, Germany) and Muller–Hinton Broth (MHB) media (Himedia, Nashik, India) were used for the bacterial species. In antifungal activity tests, Sabouraud Broth (SB) (Biolife, Milan, Italy) and Sabouraud Agar (SA) (Chemsolute, Renningen, Germany) were used for *Candida* species. Four different Gram‐positive and Gram‐negative bacteria were used for the antibacterial activity of fixed oil from *S. minor*. Of these, *Escherichia coli* (ATCC 10536), *Klebsiella pneumoniae* (ATCC 13883), *Staphylococcus aureus* (ATCC 12600), and *Enterobacter aerogenes* (ATCC 13048) were supplied by the American Type Culture Collection. Five different *Candida* species were used for the antifungal activity of fixed oil from *S. minor*. These species are *Candida tropicalis* (ATCC 13803), *Candida krusei* (ATCC 14 243), *Candida parapsilosis* (ATCC 22019), *Candida glabrata* (ATCC 2001), and *Candida albicans* (ATCC 14053), all obtained from the American Type Culture Collection.

#### 5.6.2 Determination of MICs and MBCs/MFCs

In order to determine the antibacterial and antifungal properties of *S. minor* fixed oil against the tested microorganisms, the method used in our previous studies was applied [60, 61, 62]. Therefore, the inhibitory effect of the fixed oil of the plant seed was studied using the resazurin‐based broth dilution method. First, 81 µL of the fixed oil was dissolved in 900 µL of DMSO and vortexed sufficiently at room temperature for sufficient dissolution. 100 µL of the DMSO solution containing the fixed oil was distributed into the first wells of the microplate. Therefore, the fixed oil concentration varied between 45 and 0.088 µL/mL from the first well to the tenth well of the microplate. Then, 1 µL of the standards prepared for each microorganism tested was inoculated into each well. The microplates, kept in the incubator at 36ºC overnight, were treated with resazurin the next day and put back in the incubator for 3–4 h. The MIC values of the seed fixed oil were determined by observing the color change on the microplate. Then, to determine MBCs and MFCs, samples were taken from the first wells on the microplate where no growth was observed, and the inhibitory properties of the fixed oil on microorganisms were determined by inoculating them onto MHA (for bacterial species) and SA (for *Candida* species).

### 5.7 Molecular Docking Analysis

Compounds identified in the fixed oil from *S. minor* seeds were plotted in ChemOffice program by obtaining their SMILES from PubChem (https://pubchem.ncbi.nlm.nih.gov) and energy minimizations were performed. Molecular docking studies were performed using a standard procedure to determine the binding modes of all compounds in the active site of 1HSK macromolecule to evaluate antimicrobial activity and in 1EA1 macromolecule to evaluate antifungal activity [28, 29, 30, 31, 32, 33, 34, 35, 36, 37, 38, 39, 40, 41, 42, 43, 44, 45, 46, 47, 48, 49, 50, 51, 52, 53, 54, 55, 56, 57, 58, 59, 60, 61, 62, 63]. Crystal structures of the macromolecules were retrieved from the Protein Data Bank (PDB) server (https://www.rcsb.org/, accessed July 2, 2025). Macromolecules were optimized using the Autodock and Maestro programs. Both Autodock [64] and Vina [65] programs were used for molecular docking studies. As we have done similar work before [30, 60], FAD was redocked to the target site of the macromolecule (1HSK) to validate the docking program and the RMSD value was found to be less than two (<1.65) in the same way Fluconazole (TPF) was redocked to the target site of the macromolecule (1EA1) to validate the docking program and the RMSD value was found to be less than one (<0.99). The results were visualized using Maestro viewer [66].

### 5.8 ADME Predictions

The SwissADME website was used to calculate the physicochemical properties of the compounds detected in fixed oil from S. minor seeds, such as fat solubility, water solubility, gastrointestinal absorption, and blood–brain barrier permeability (**1–14**), and then to make predictions based on the results (http://www.swissadme.ch/, access date: 02.07.2025) [67, 68, 69].

## Author Contributions

Z.T. and T.U. designed the research. Z.T., T.U., H.U., and B.G. performed the research. Z.T., T.U., H.U., and B.G. contributed data. Z.T., T.U., H.U., and B.G. analyzed data. Z.T., T.U., H.U., and B.G. wrote the article.

## Conflicts of Interest

The authors declare no conflicts of interest.

## Supporting information

Supplementary Material

## Data Availability

The data that support the findings of this study are available from the corresponding author upon reasonable request.
