# Supplementary material for: Exploring Antimicrobial Activities of Fixed Oil From Sanguisorba minor Seeds by In Vitro and In Silico Analysis
Source: ChemistryOpen. 2026 Jul 2;15(7):e70250. doi: 10.1002/open.70250 (PMC13328840; doi:10.1002/open.70250)
Supplement: Supplementary file 1 — Supplementary Material [file OPEN-15-e70250-s001.pdf]

Exploring Antimicrobial Activities of Fixed Oil from *Sanguisorba Minor* Seeds by *In Vitro* and *In Silico* Analysis

Zehra Torun*[a] , Tuba Unver[b], Harun Uslu[c], Bunyamin Goktas[c,d]

[a] Dr. Z. Torun
Department of Pharmacognosy
Inonu University, Faculty of Pharmacy
Malatya/Turkiye
E-mail: zehra.torun@inonu.edu.tr

[b] Doc. Dr. T. Unver
Department of Pharmaceutical Microbiology
Inonu University, Faculty of Pharmacy
Malatya/Turkiye

[c] Dr. H. Uslu
Department of Pharmaceutical Chemistry
Fırat University, Faculty of Pharmacy
Elazıg/Turkiye

[d] Ass. Prof. B. Goktas
Department of Pharmaceutical Chemistry
Anadolu University, Graduate School
Eskisehir/Turkiye

Graphical Abstract

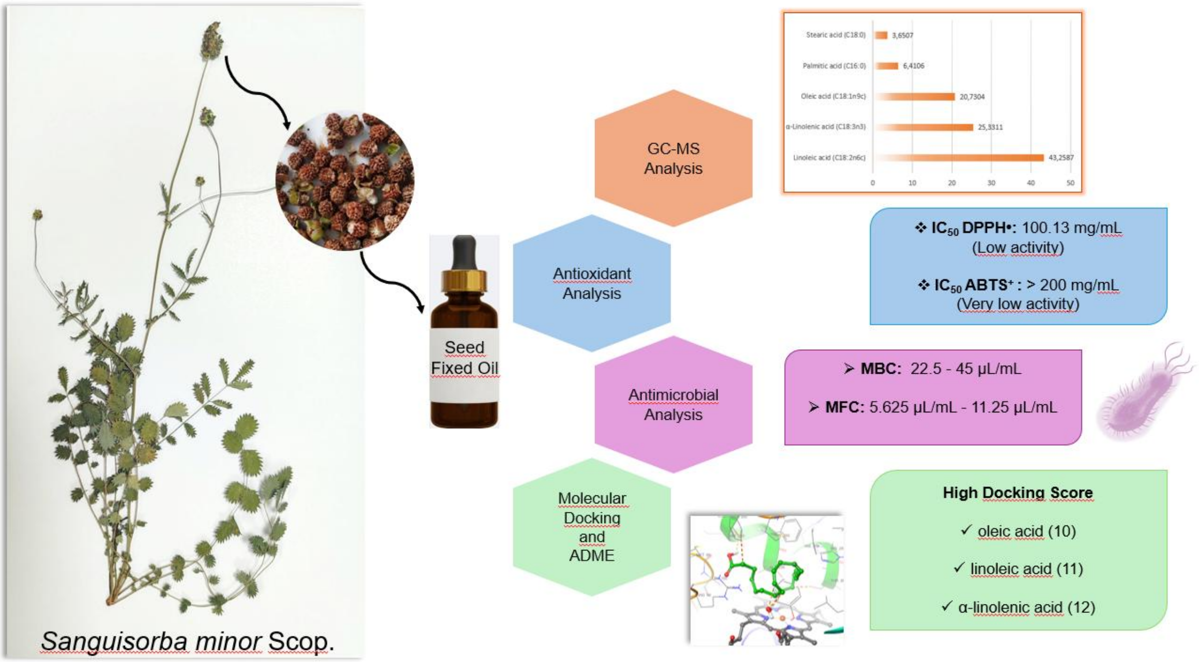

CONTENT LISTS

Figure 1S. 2D interaction diagram with 1HSK for **1**......1
Figure 2S. 3D interaction diagram with 1HSK for **1**......1
Figure 3S. 2D interaction diagram with 1HSK for **2**......2
Figure 4S. 3D interaction diagram with 1HSK for **2**......2
Figure 5S. 2D interaction diagram with 1HSK for **3**......3
Figure 6S. 3D interaction diagram with 1HSK for **3**......3
Figure 7S. 2D interaction diagram with 1HSK for **4**......4
Figure 8S. 3D interaction diagram with 1HSK for **4**......4
Figure 9S. 2D interaction diagram with 1HSK for **5**......5
Figure 10S. 3D interaction diagram with 1HSK for **5**......5
Figure 11S. 2D interaction diagram with 1HSK for **6**......6
Figure 12S. 3D interaction diagram with 1HSK for **6**......6
Figure 13S. 2D interaction diagram with 1HSK for **7**......7
Figure 14S. 3D interaction diagram with 1HSK for **7**......7
Figure 15S. 2D interaction diagram with 1HSK for **8**......8
Figure 16S. 3D interaction diagram with 1HSK for **8**......8
Figure 17S. 2D interaction diagram with 1HSK for **9**......9
Figure 18S. 3D interaction diagram with 1HSK for **9**......9
Figure 19S. 2D interaction diagram with 1HSK for **10**......10
Figure 20S. 3D interaction diagram with 1HSK for **10**......10
Figure 21S. 2D interaction diagram with 1HSK for **11**......11
Figure 22S. 3D interaction diagram with 1HSK for **11**......11
Figure 23S. 2D interaction diagram with 1HSK for **12**......12
Figure 24S. 3D interaction diagram with 1HSK for **12**......12
Figure 25S. 2D interaction diagram with 1HSK for **13**......13
Figure 26S. 3D interaction diagram with 1HSK for **13**......13
Figure 27S. 2D interaction diagram with 1HSK for **14**......14
Figure 28S. 3D interaction diagram with 1HSK for **14**......14
Figure 29S. 2D interaction diagram with 1EA1 for **1**......15
Figure 30S. 3D interaction diagram with 1EA1 for **1**......15
Figure 31S. 2D interaction diagram with 1EA1 for **2**......16
Figure 32S. 3D interaction diagram with 1EA1 for **2**......16
Figure 33S. 2D interaction diagram with 1EA1 for **3**......17
Figure 34S. 3D interaction diagram with 1EA1 for **3**......17
Figure 35S. 2D interaction diagram with 1EA1 for **4**......18

Figure 36S. 3D interaction diagram with 1EA1 for **4**......18
Figure 37S. 2D interaction diagram with 1EA1 for **5**......19
Figure 38S. 3D interaction diagram with 1EA1 for **5**......19
Figure 39S. 2D interaction diagram with 1EA1 for **6**......20
Figure 40S. 3D interaction diagram with 1EA1 for **6**......20
Figure 41S. 2D interaction diagram with 1EA1 for **7**......21
Figure 42S. 3D interaction diagram with 1EA1 for **7**......21
Figure 43S. 2D interaction diagram with 1EA1 for **8**......22
Figure 44S. 3D interaction diagram with 1EA1 for **8**......22
Figure 45S. 2D interaction diagram with 1EA1 for **9**......23
Figure 46S. 3D interaction diagram with 1EA1 for **9**......23
Figure 47S. 2D interaction diagram with 1EA1 for **10**......24
Figure 48S. 3D interaction diagram with 1EA1 for **10**......24
Figure 49S. 2D interaction diagram with 1EA1 for **11**......25
Figure 50S. 3D interaction diagram with 1EA1 for **11**......25
Figure 51S. 2D interaction diagram with 1EA1 for **12**......26
Figure 52S. 3D interaction diagram with 1EA1 for **12**......26
Figure 53S. 2D interaction diagram with 1EA1 for **13**......27
Figure 54S. 3D interaction diagram with 1EA1 for **13**......27
Figure 55S. 2D interaction diagram with 1EA1 for **14**......28
Figure 56S. 3D interaction diagram with 1EA1 for **14**......28

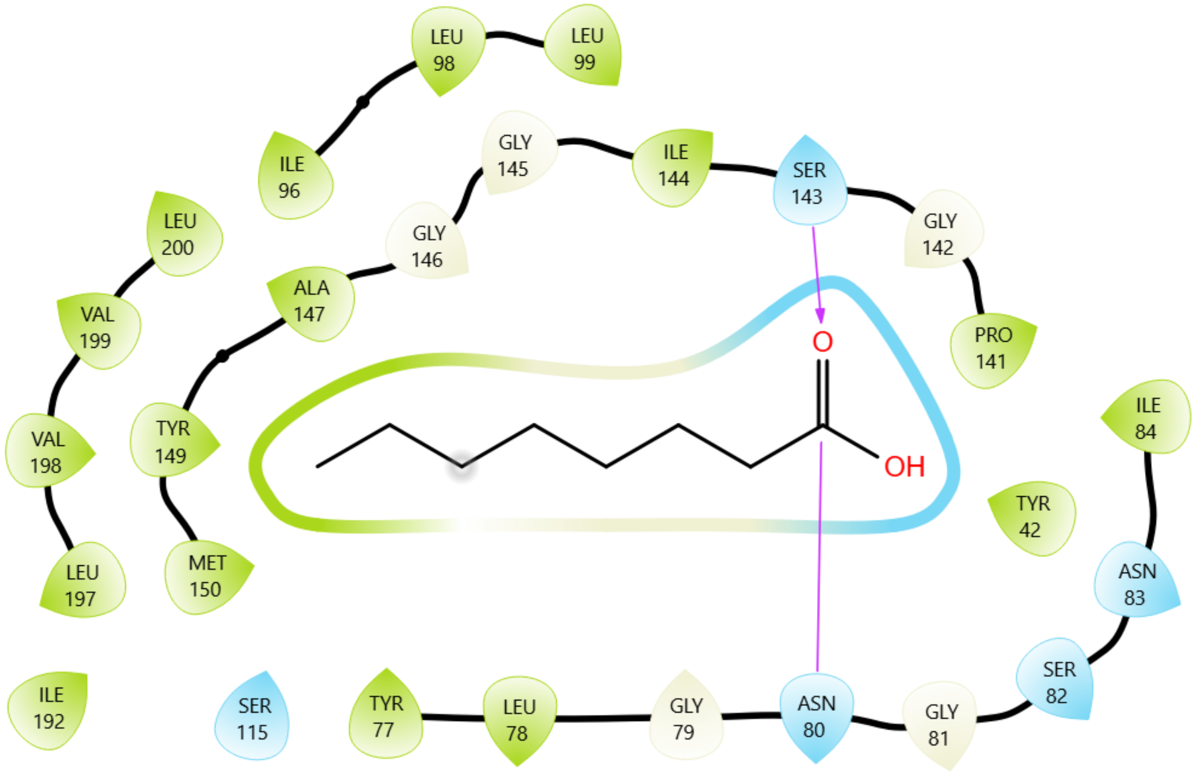

Figure 1S. 2D interaction diagram with 1HSK for **1**.

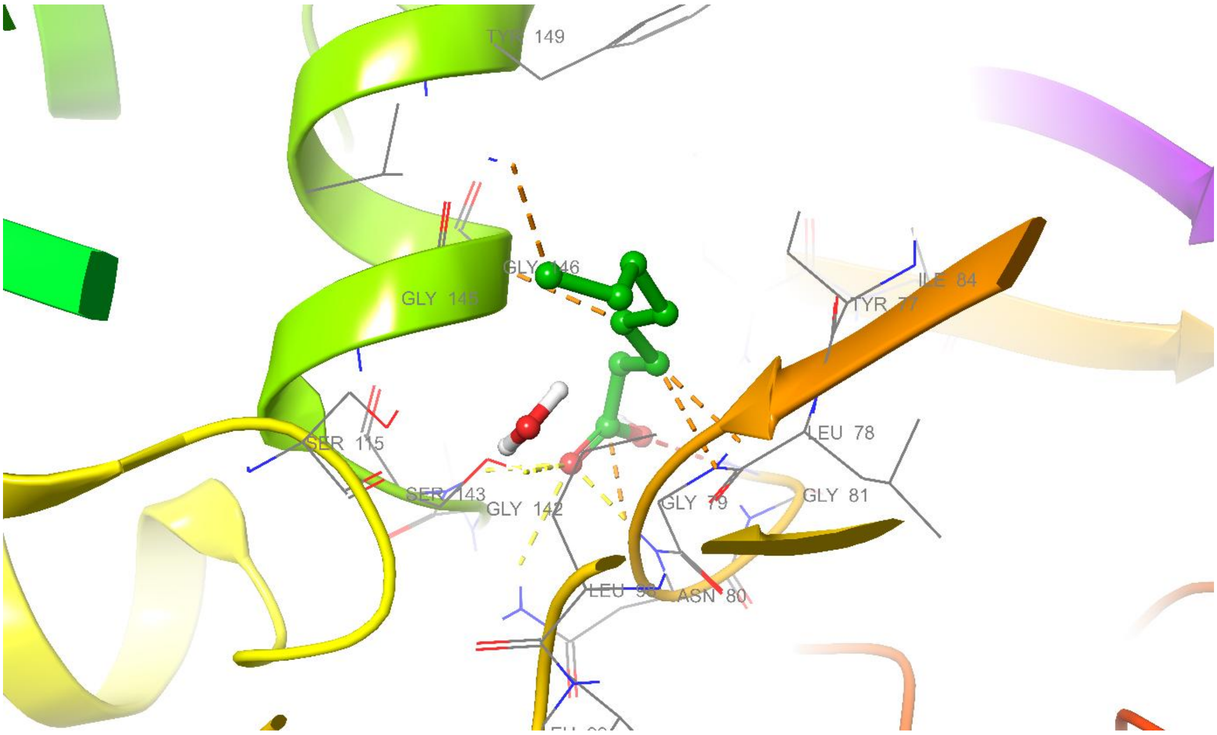

Figure 2S. 3D interaction diagram with 1HSK for **1**.

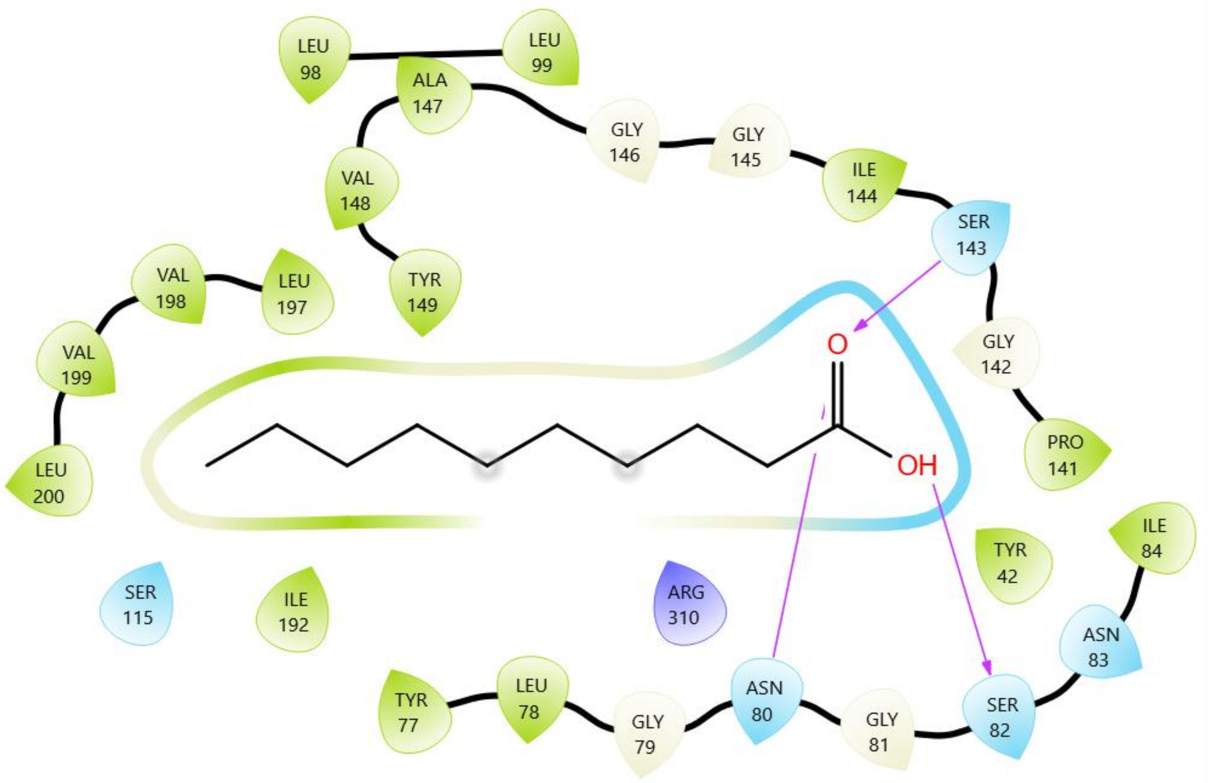

Figure 3S. 2D interaction diagram with 1HSK for **2**.

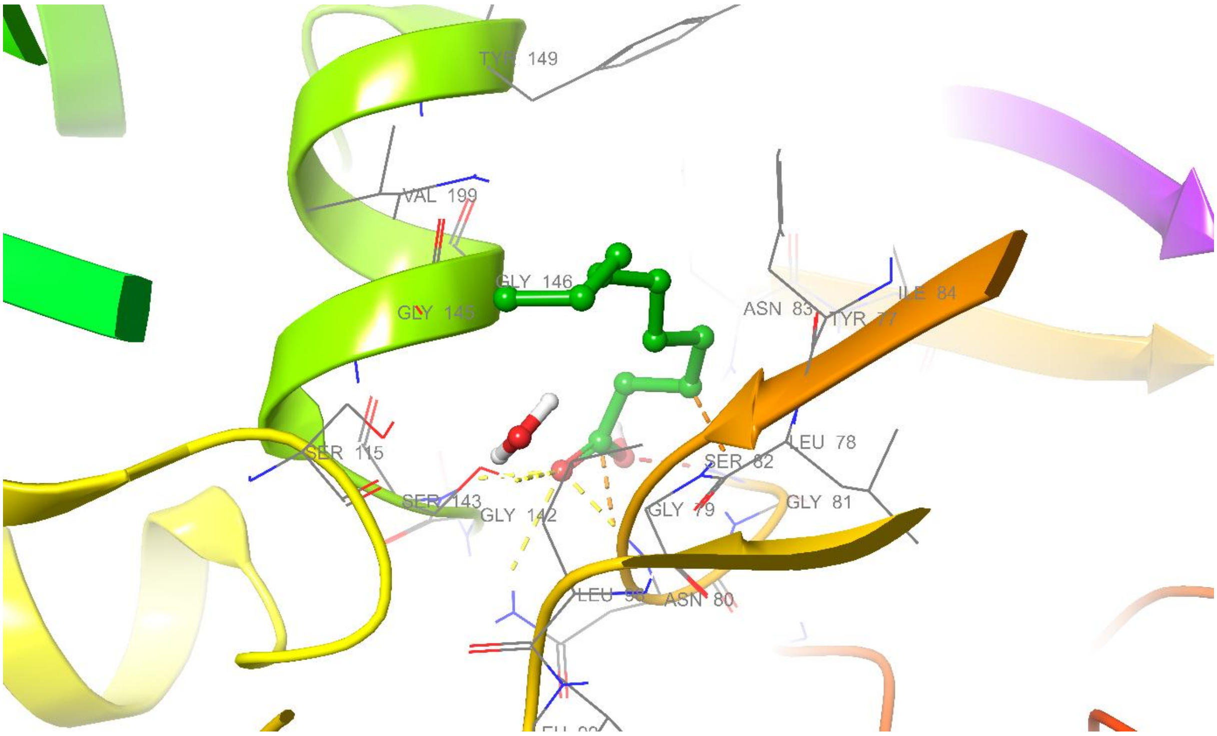

Figure 4S. 3D interaction diagram with 1HSK for **2**.

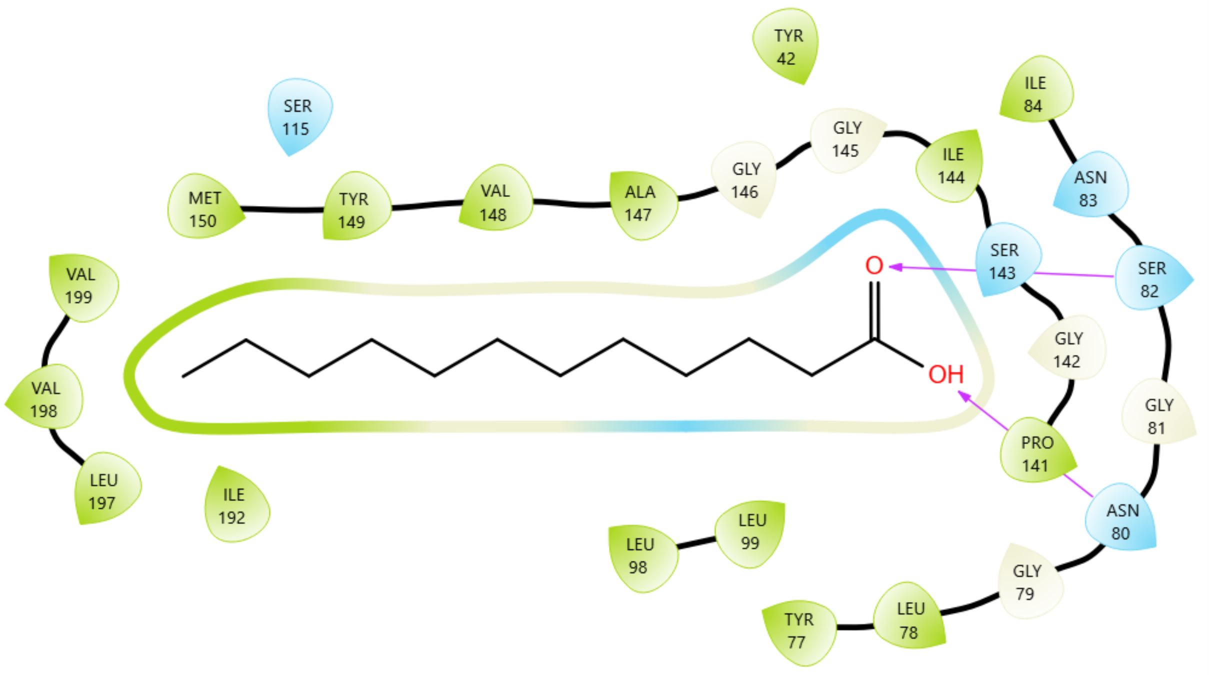

Figure 5S. 2D interaction diagram with 1HSK for **3**.

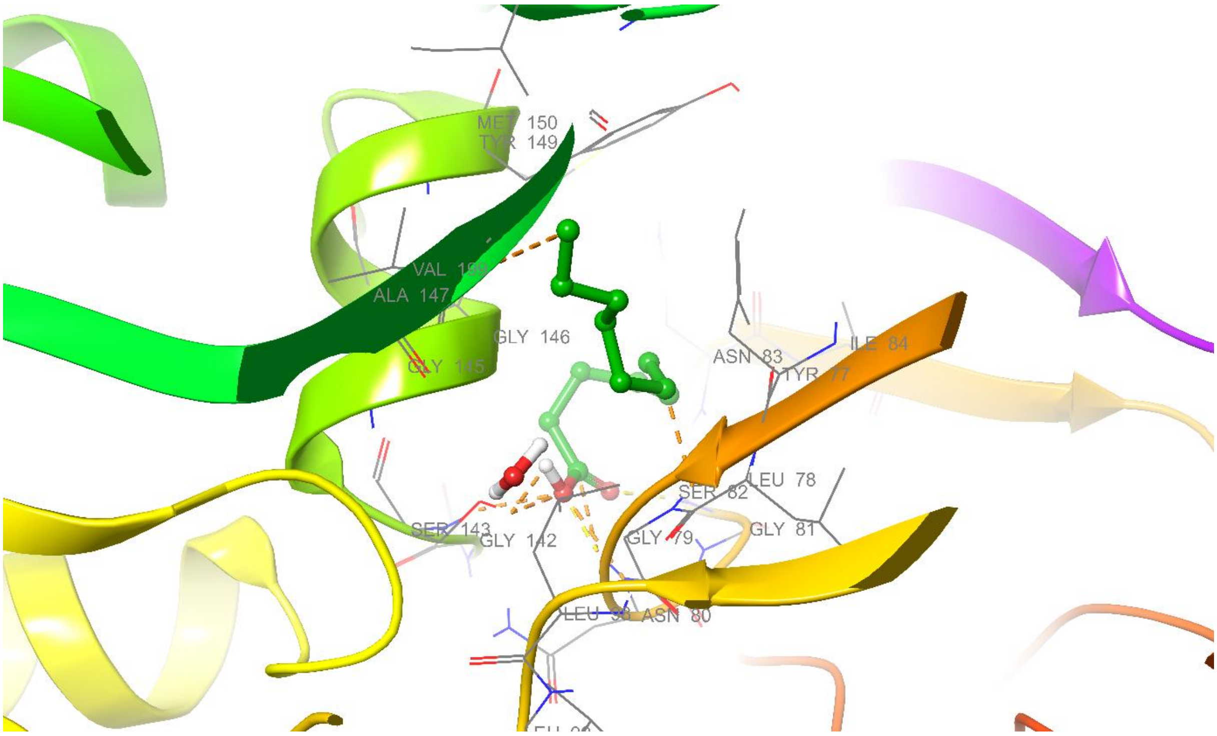

Figure 6S. 3D interaction diagram with 1HSK for **3**.

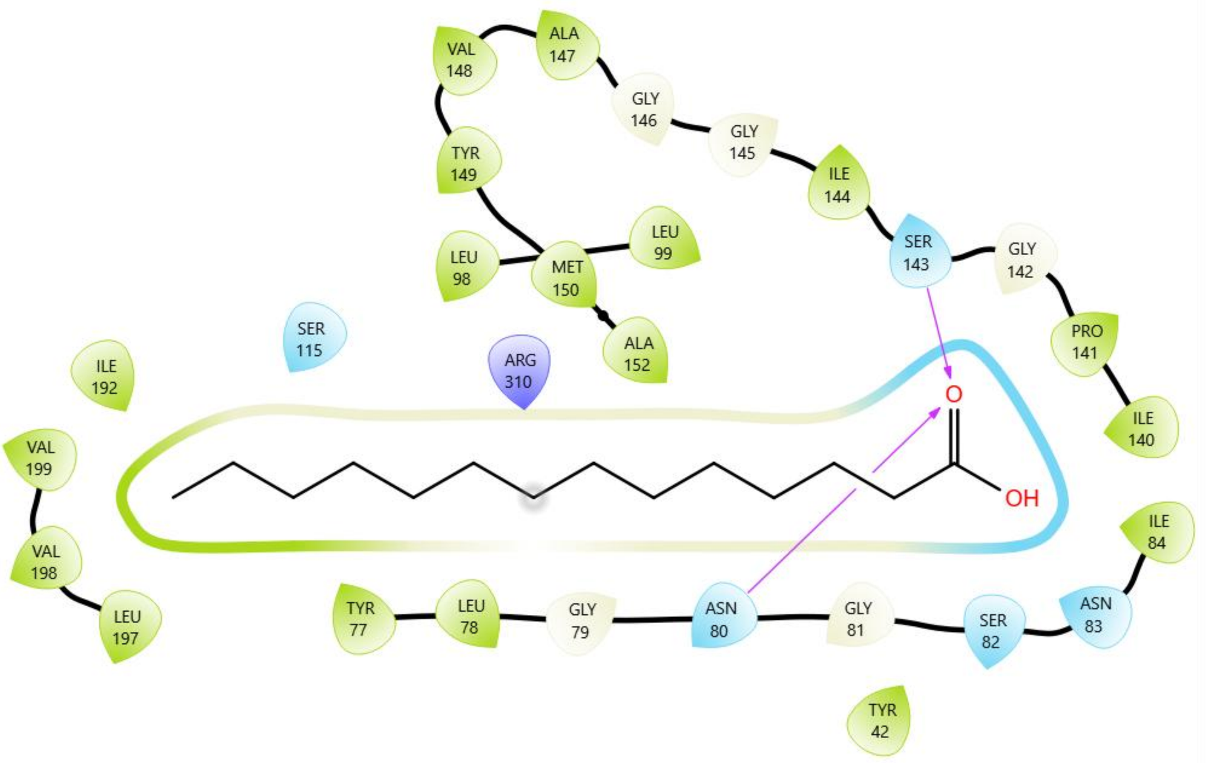

Figure 7S. 2D interaction diagram with 1HSK for **4**.

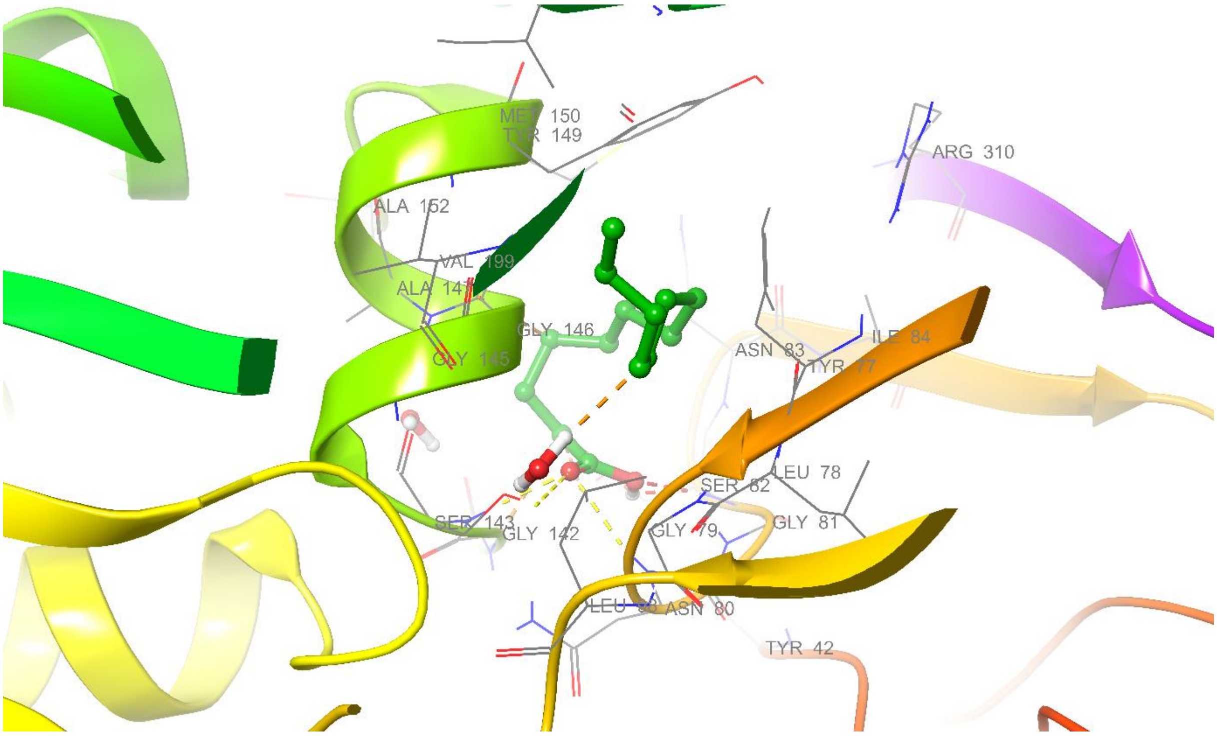

Figure 8S. 3D interaction diagram with 1HSK for **4**.

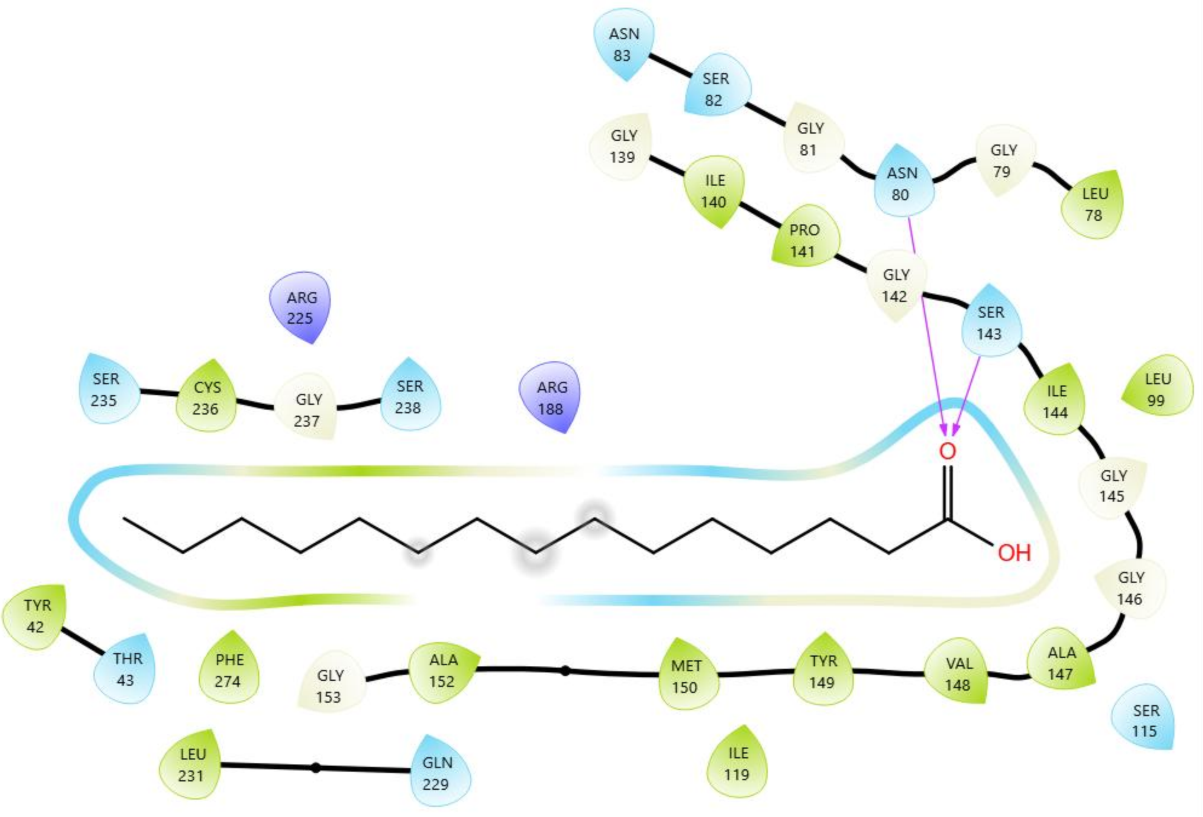

Figure 9S. 2D interaction diagram with 1HSK for **5**.

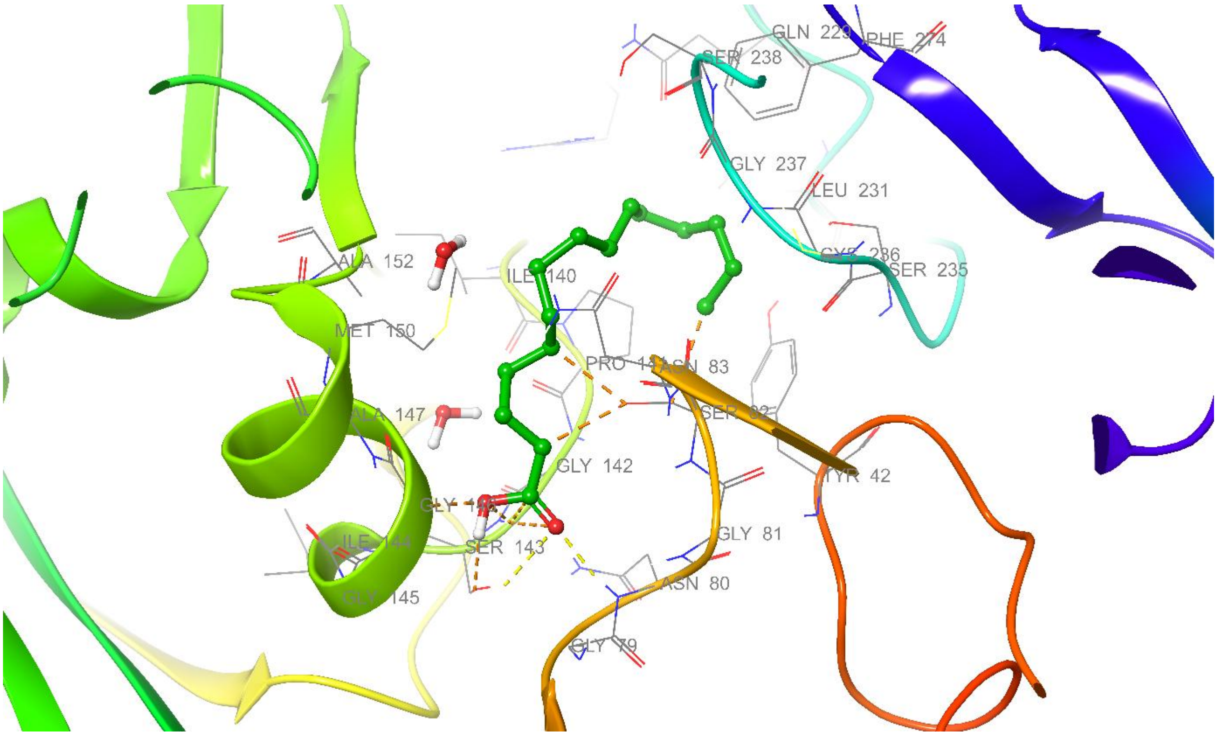

Figure 10S. 3D interaction diagram with 1HSK for **5**.

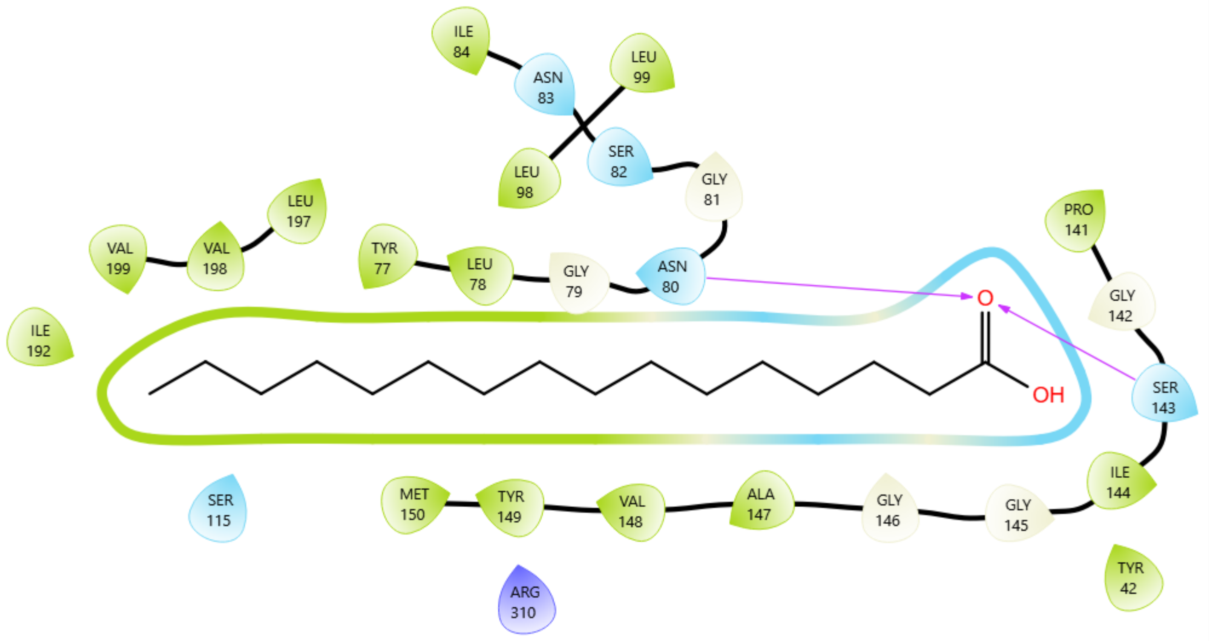

Figure 11S. 2D interaction diagram with 1HSK for **6**.

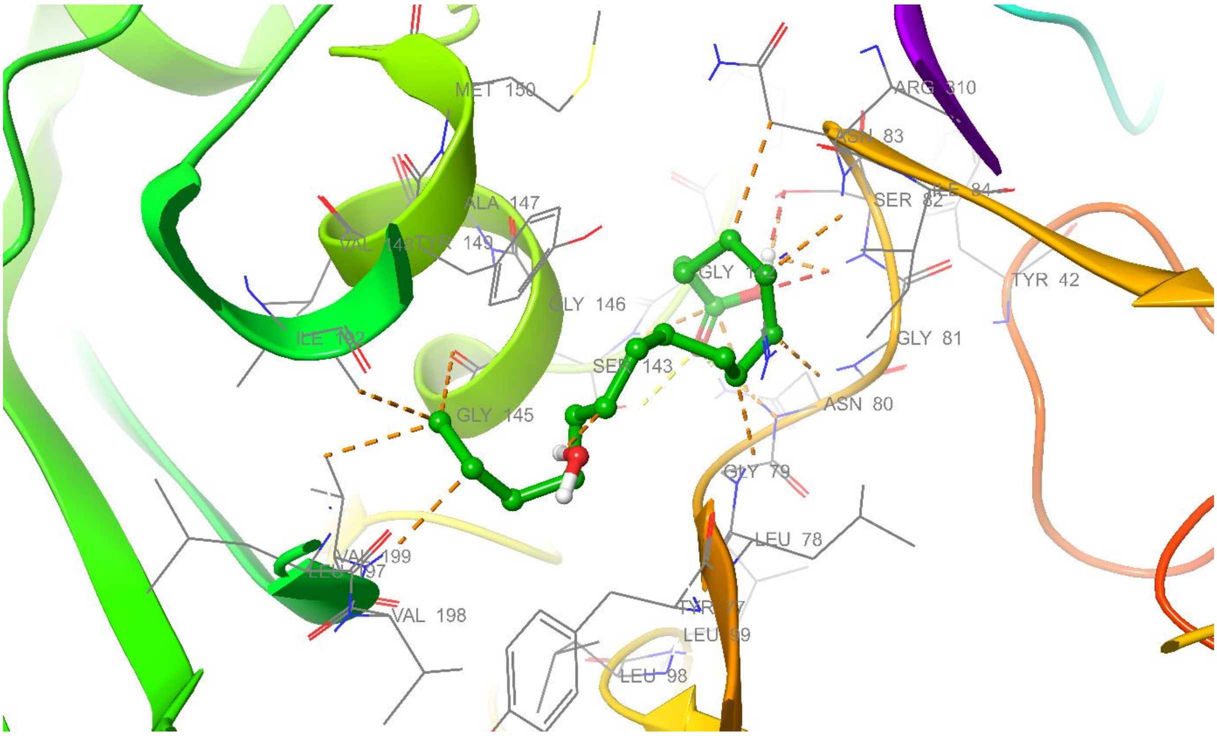

Figure 12S. 3D interaction diagram with 1HSK for **6**.

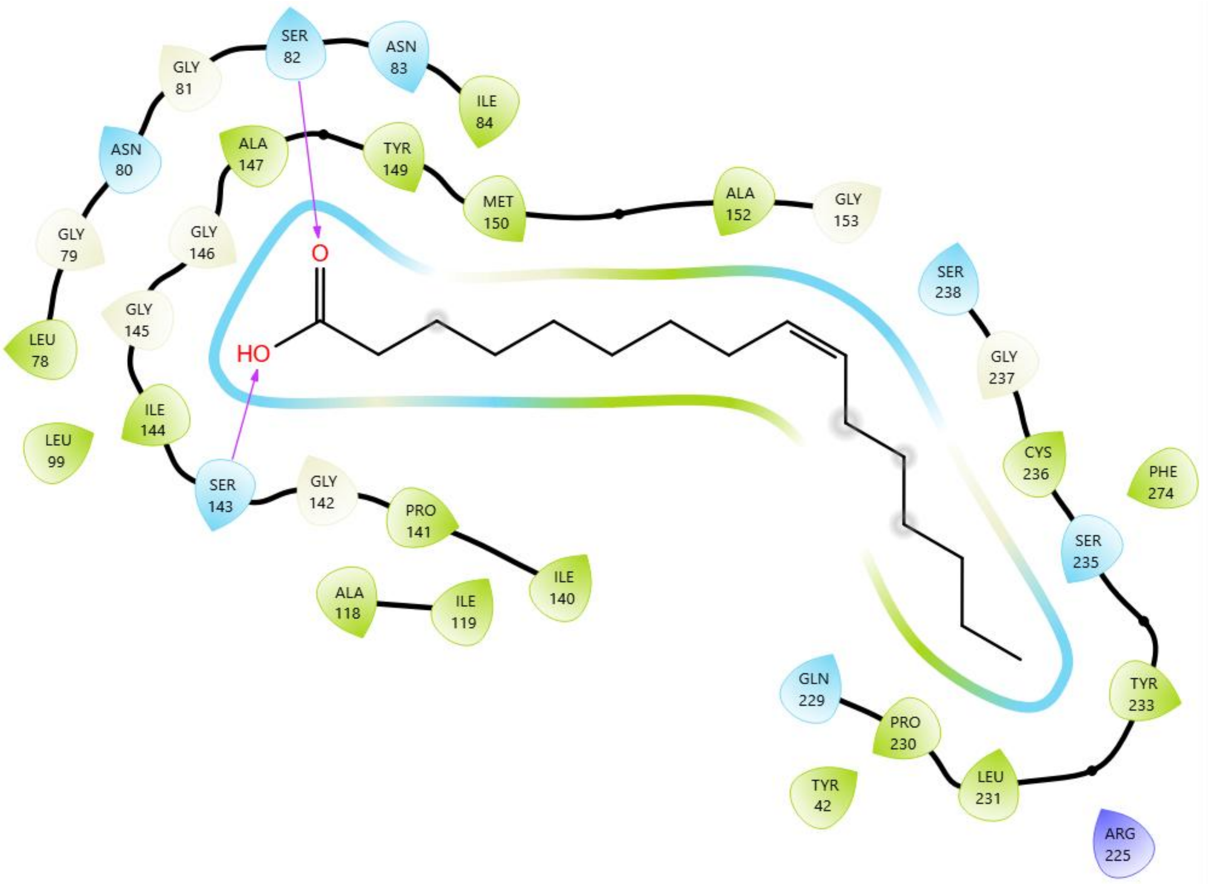

Figure 13S. 2D interaction diagram with 1HSK for **7**.

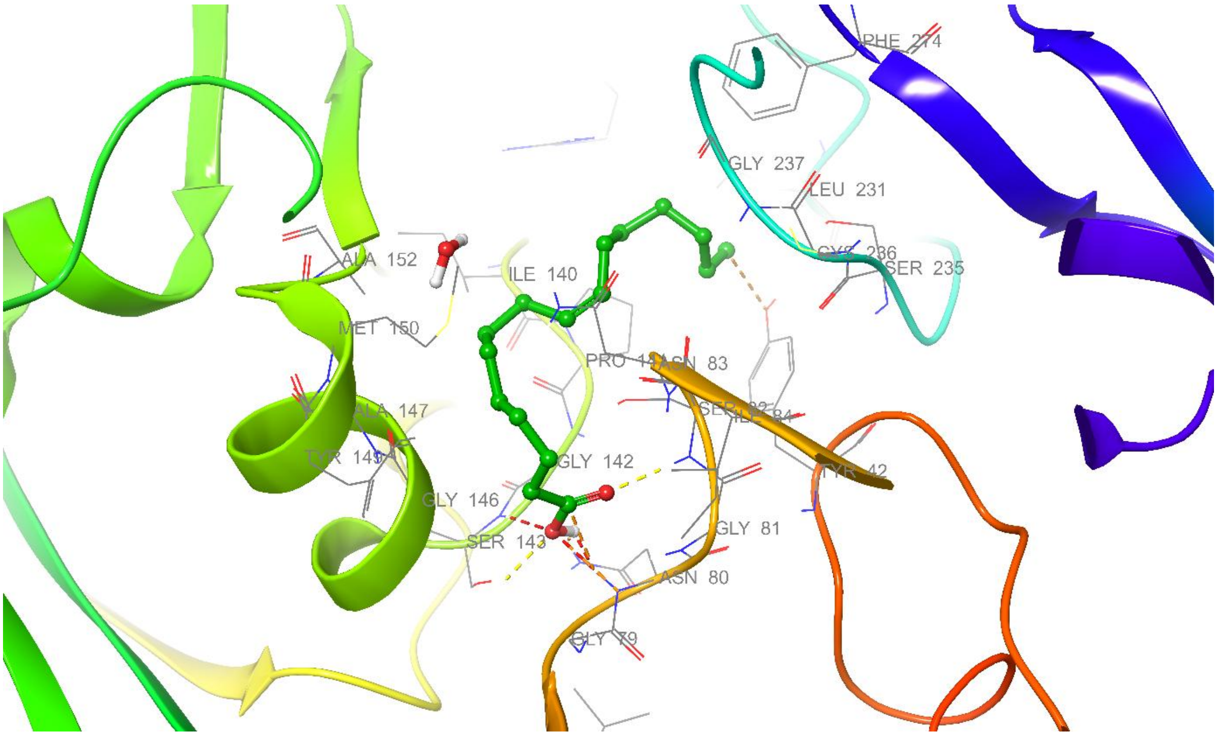

Figure 14S. 3D interaction diagram with 1HSK for **7**.

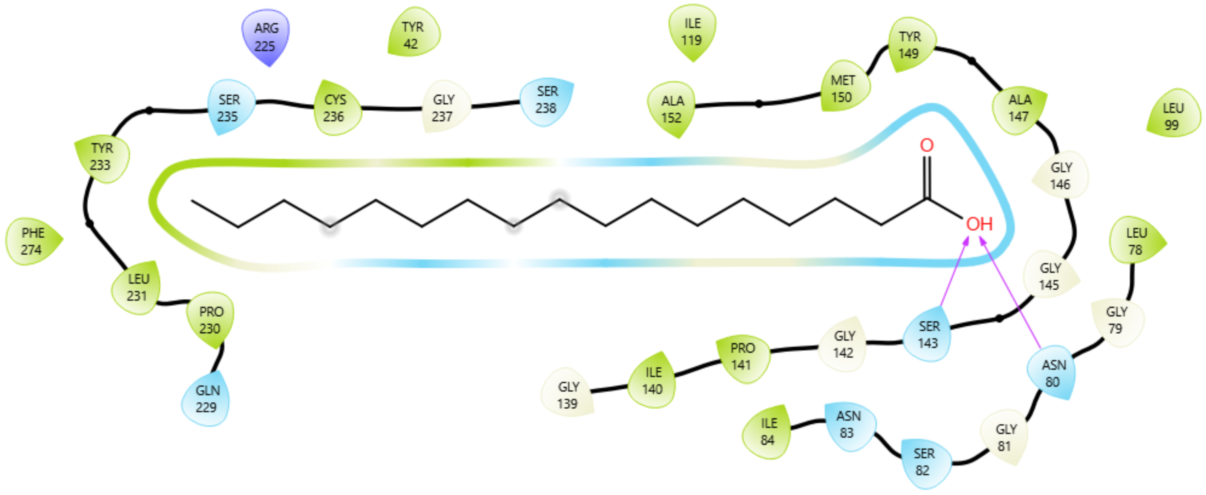

Figure 15S. 2D interaction diagram with 1HSK for **8**.

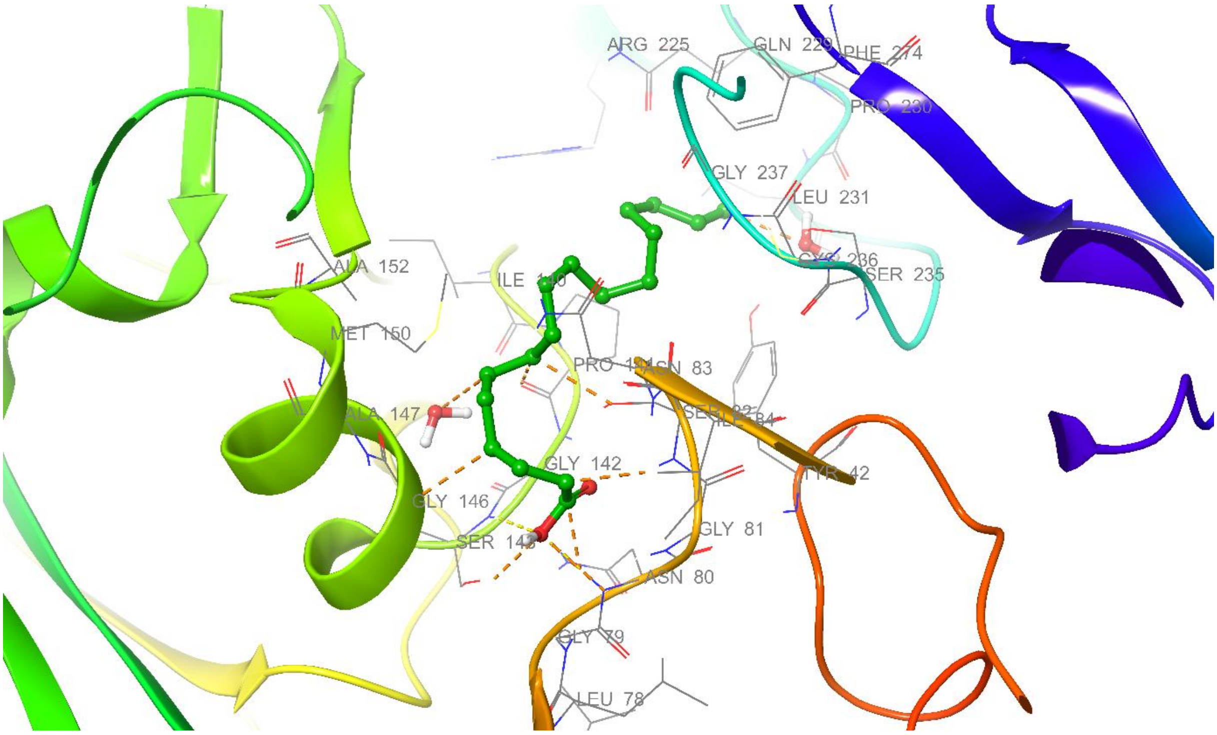

Figure 16S. 3D interaction diagram with 1HSK for **8**.

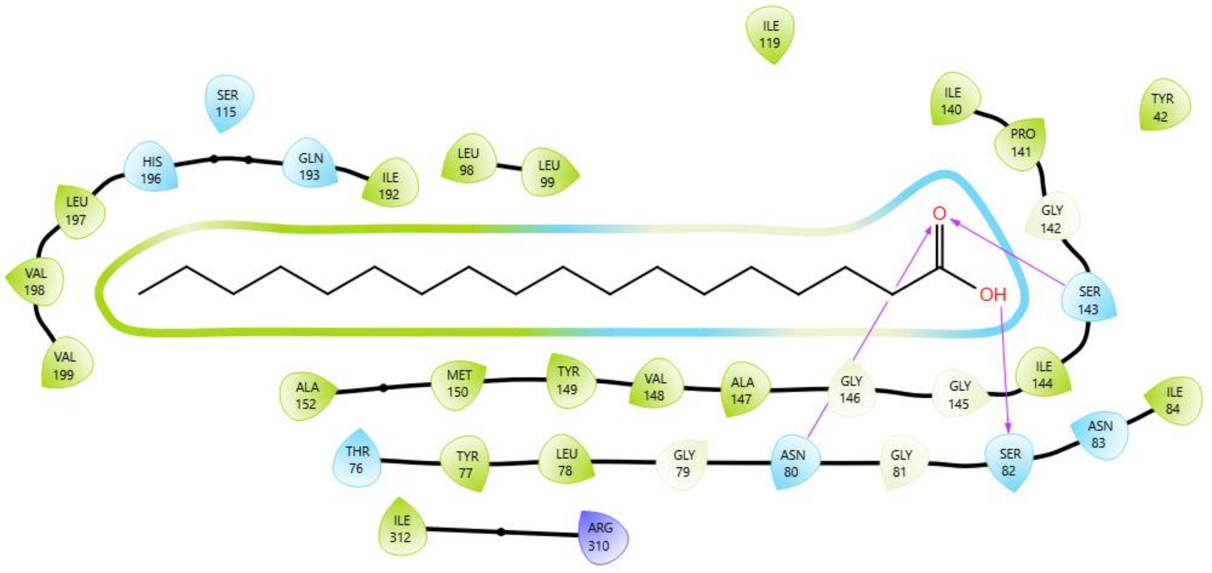

Figure 17S. 2D interaction diagram with 1HSK for **9**.

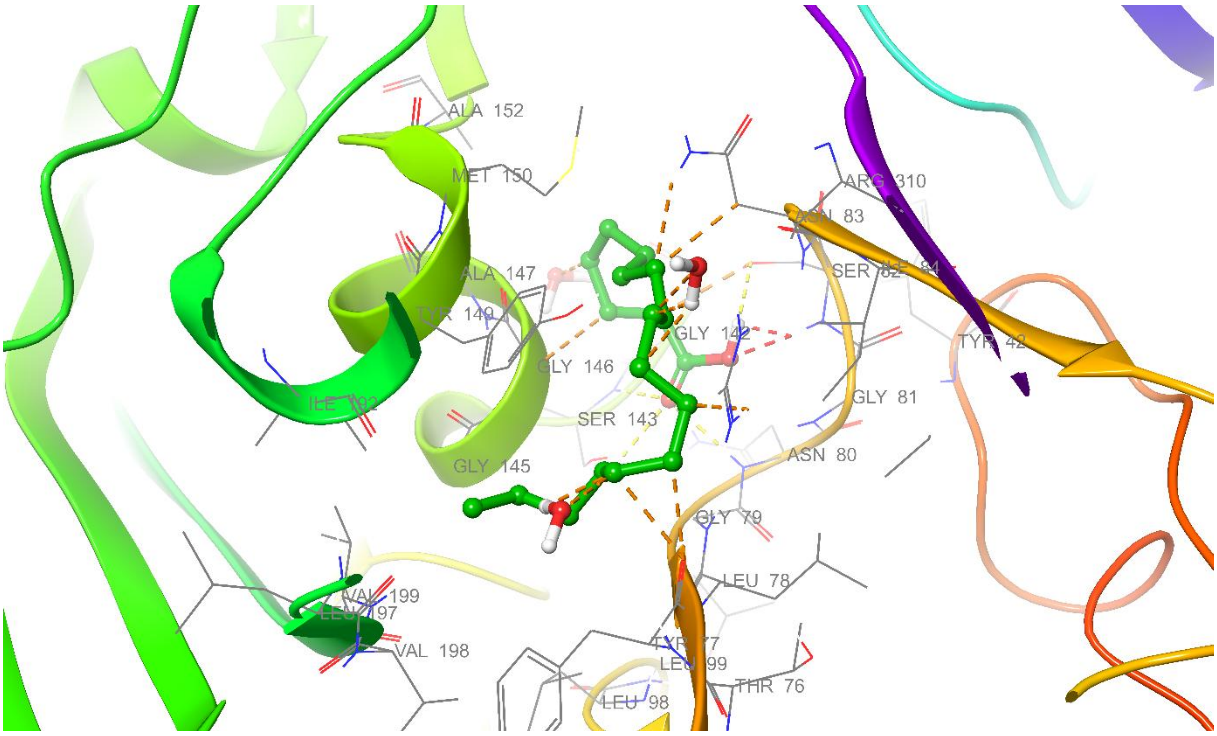

Figure 18S. 3D interaction diagram with 1HSK for **9**.

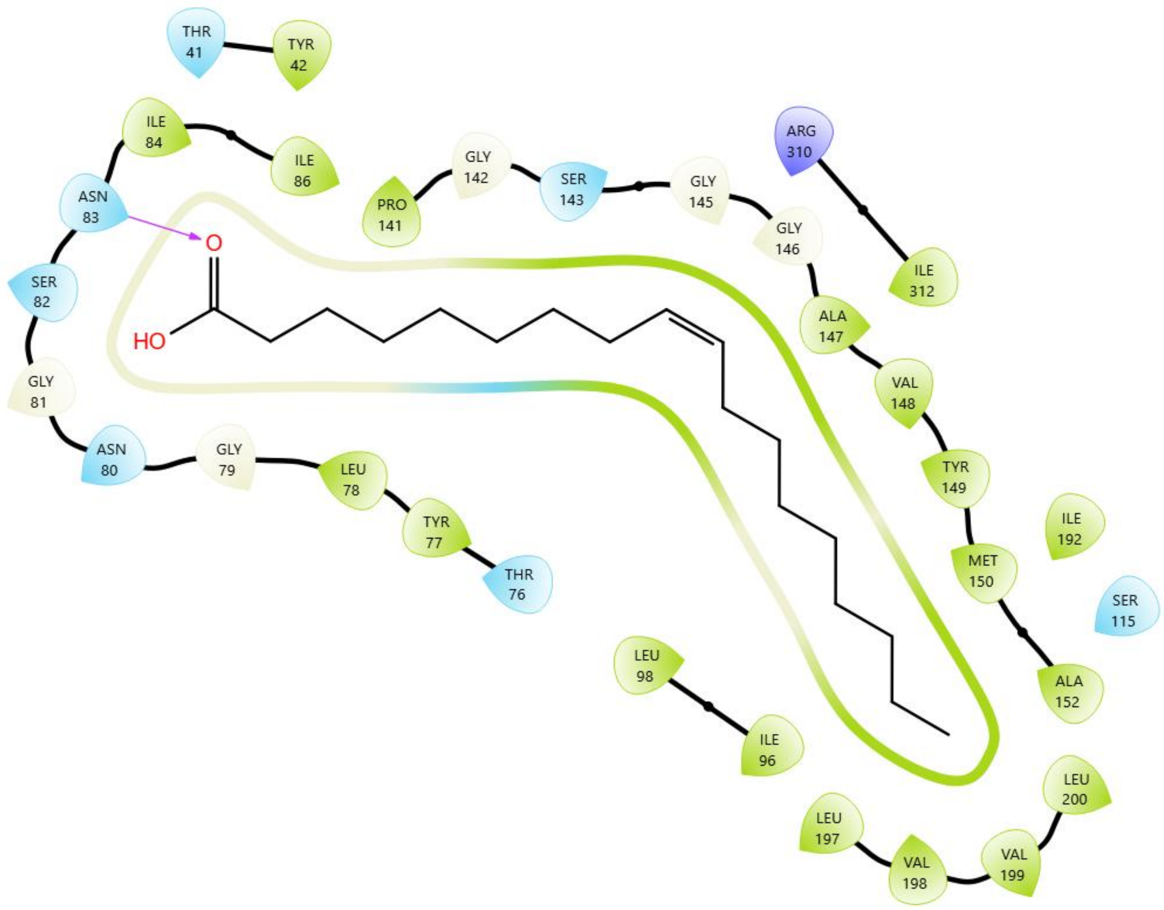

Figure 19S. 2D interaction diagram with 1HSK for **10**.

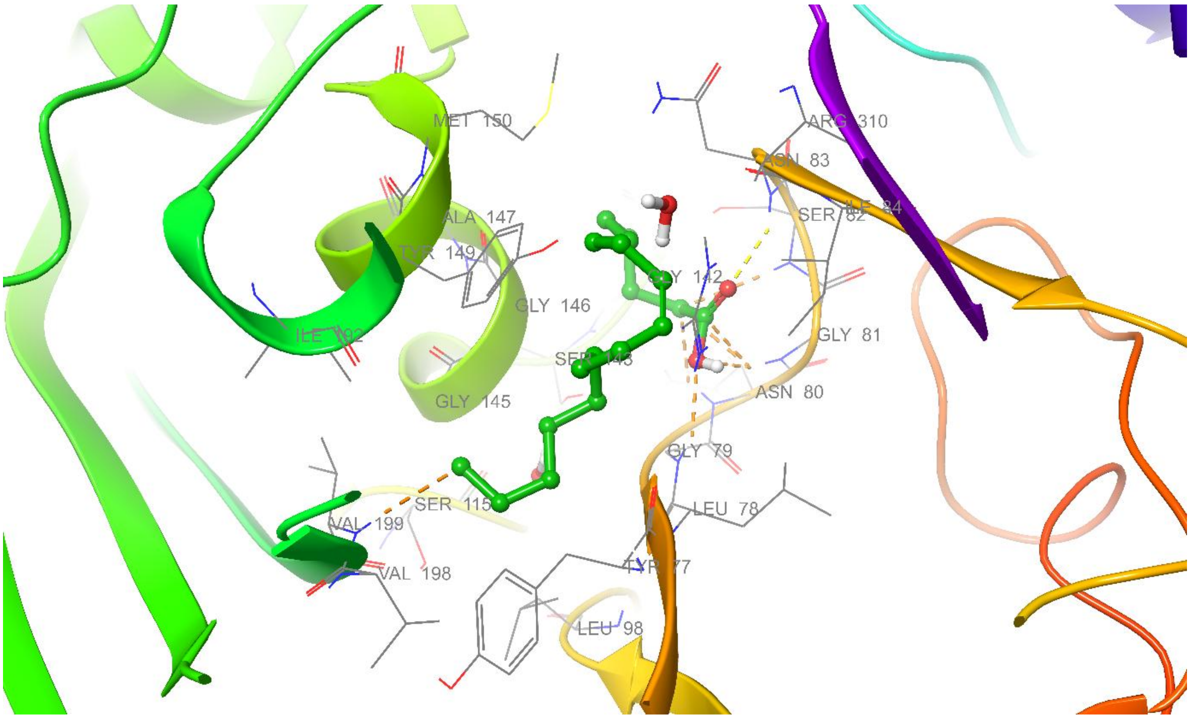

Figure 20S. 3D interaction diagram with 1HSK for **10**.

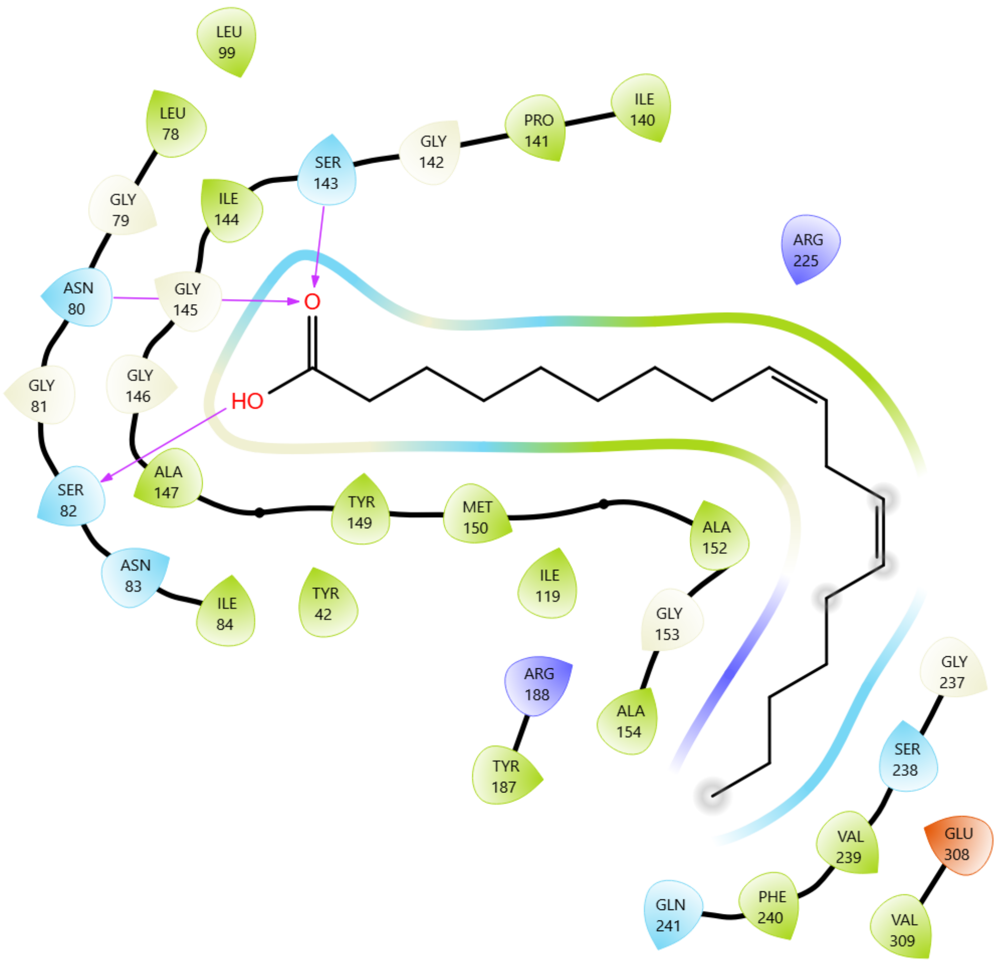

Figure 21S. 2D interaction diagram with 1HSK for **11**.

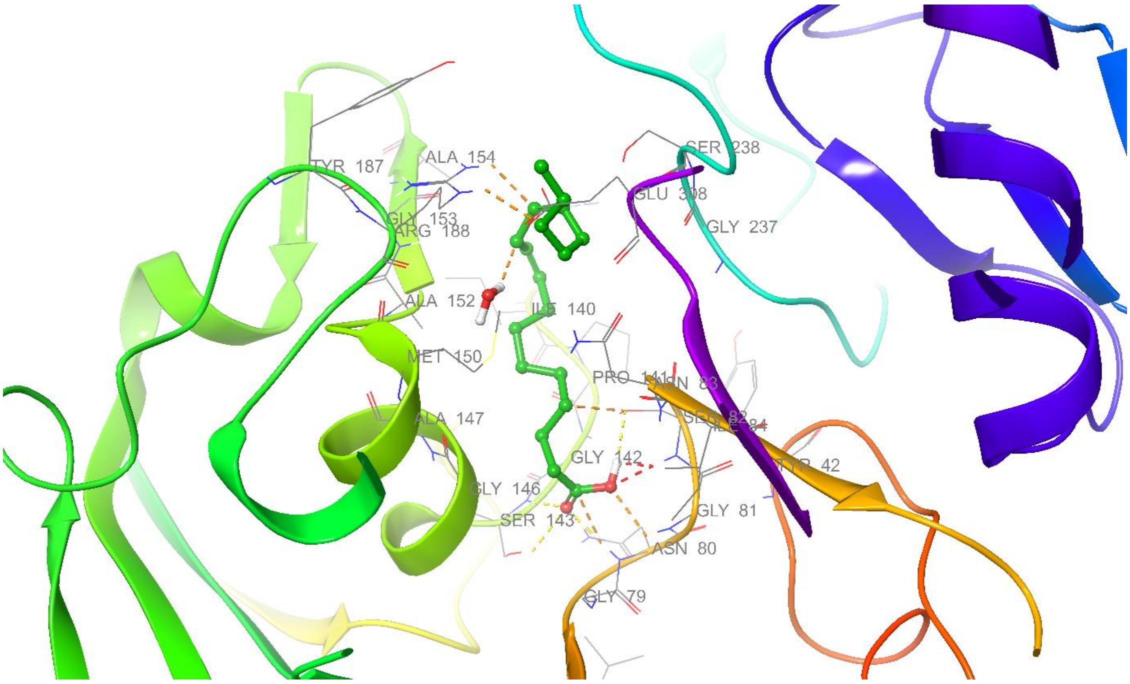

Figure 22S. 3D interaction diagram with 1HSK for **11**.

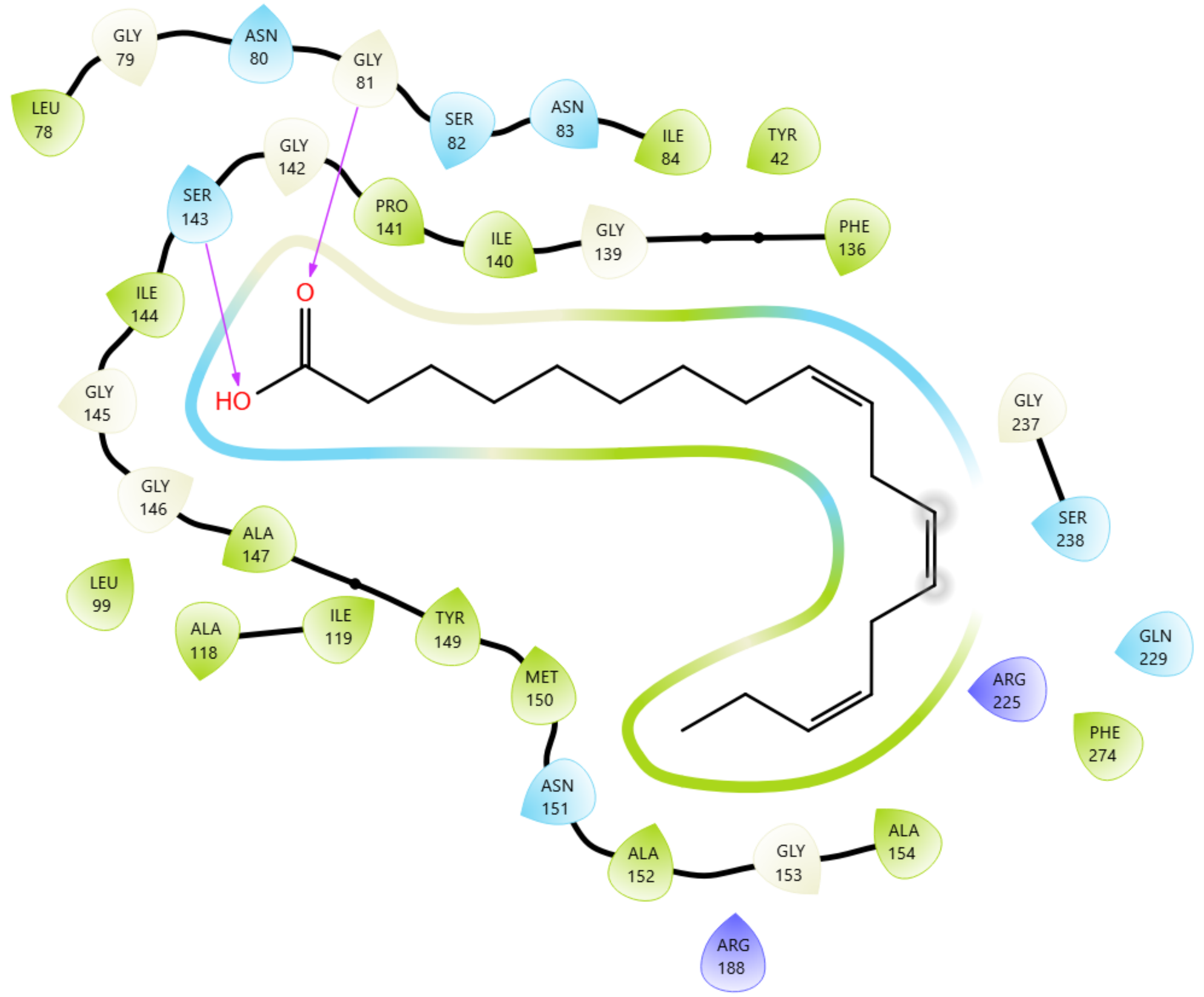

Figure 23S. 2D interaction diagram with 1HSK for **12**.

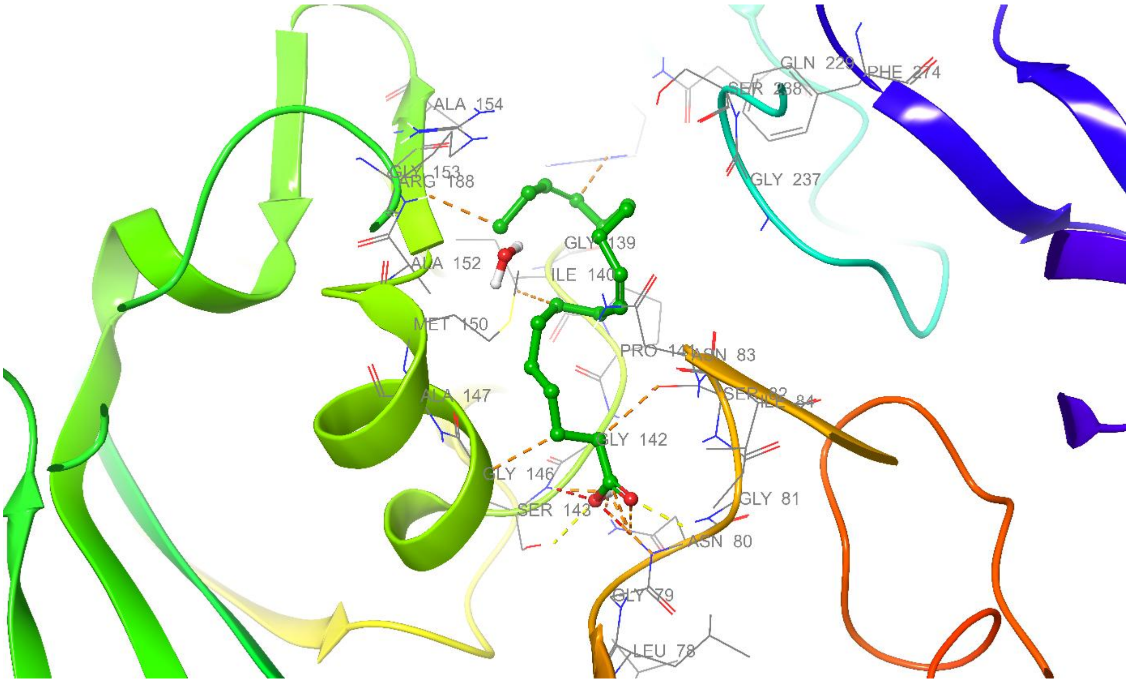

Figure 24S. 3D interaction diagram with 1HSK for **12**.

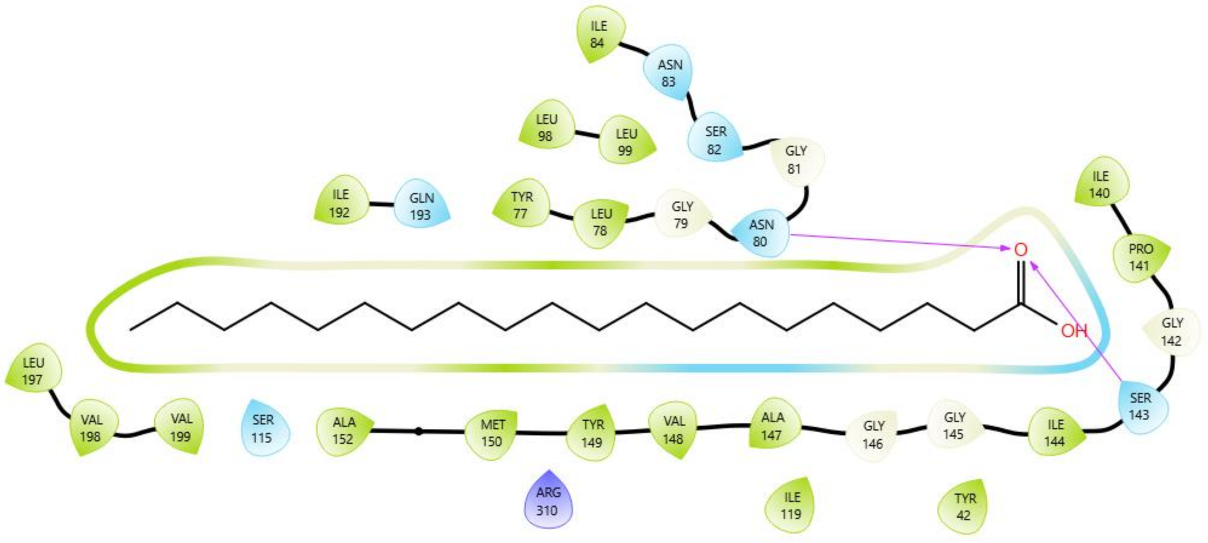

Figure 25S. 2D interaction diagram with 1HSK for **13**.

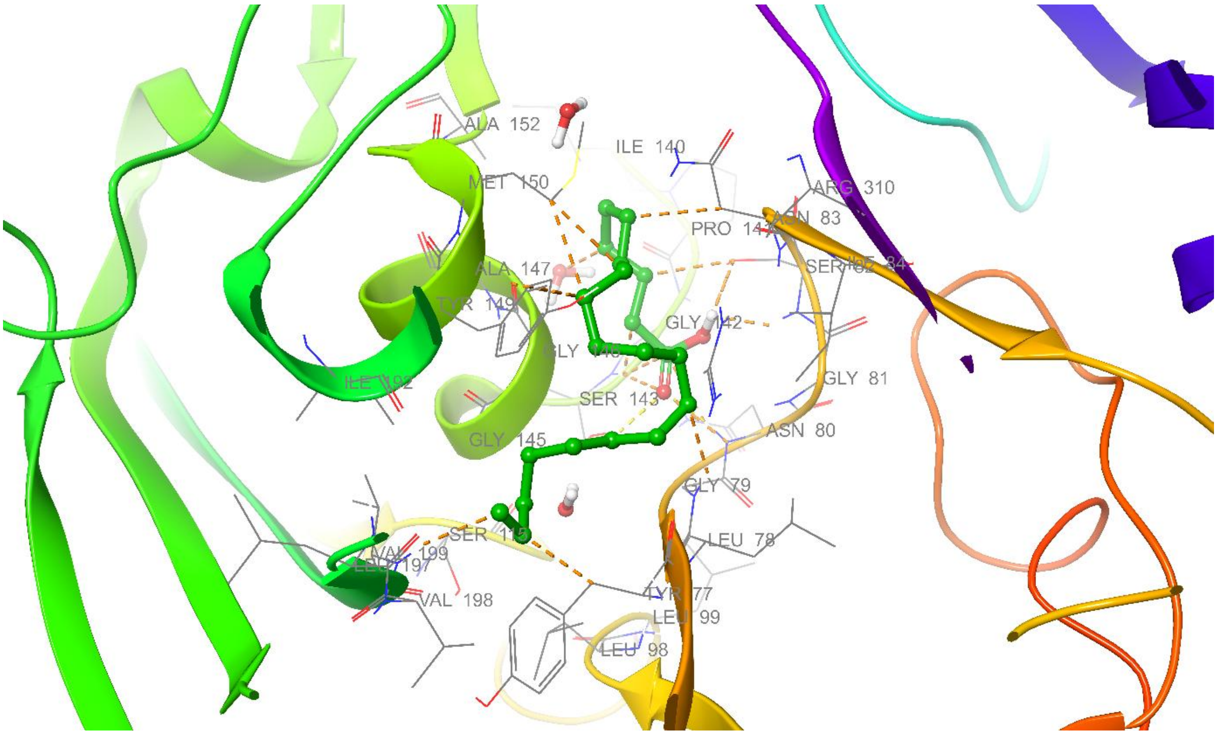

Figure 26S. 3D interaction diagram with 1HSK for **13**.

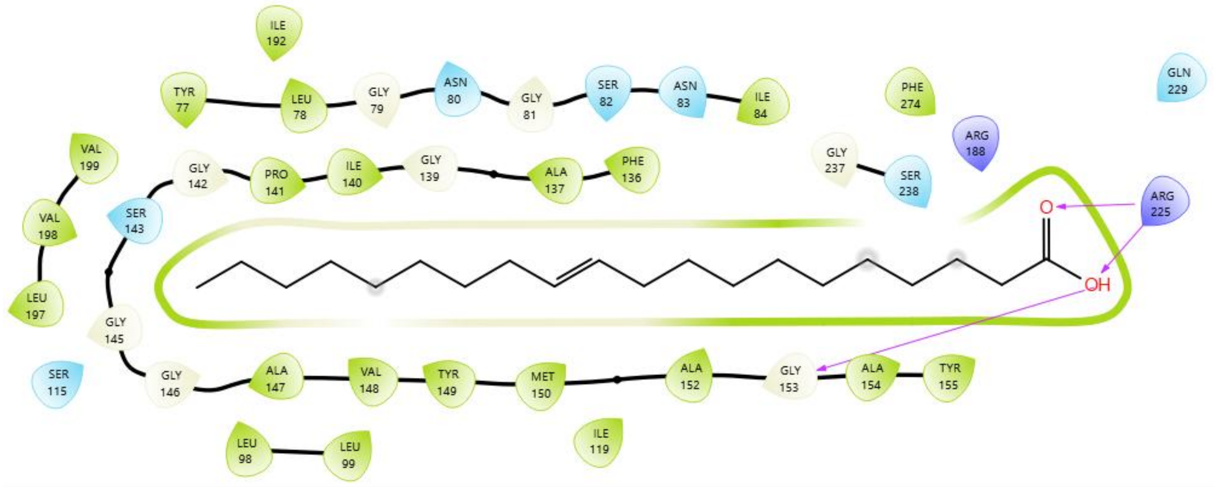

Figure 27S. 2D interaction diagram with 1HSK for **14**.

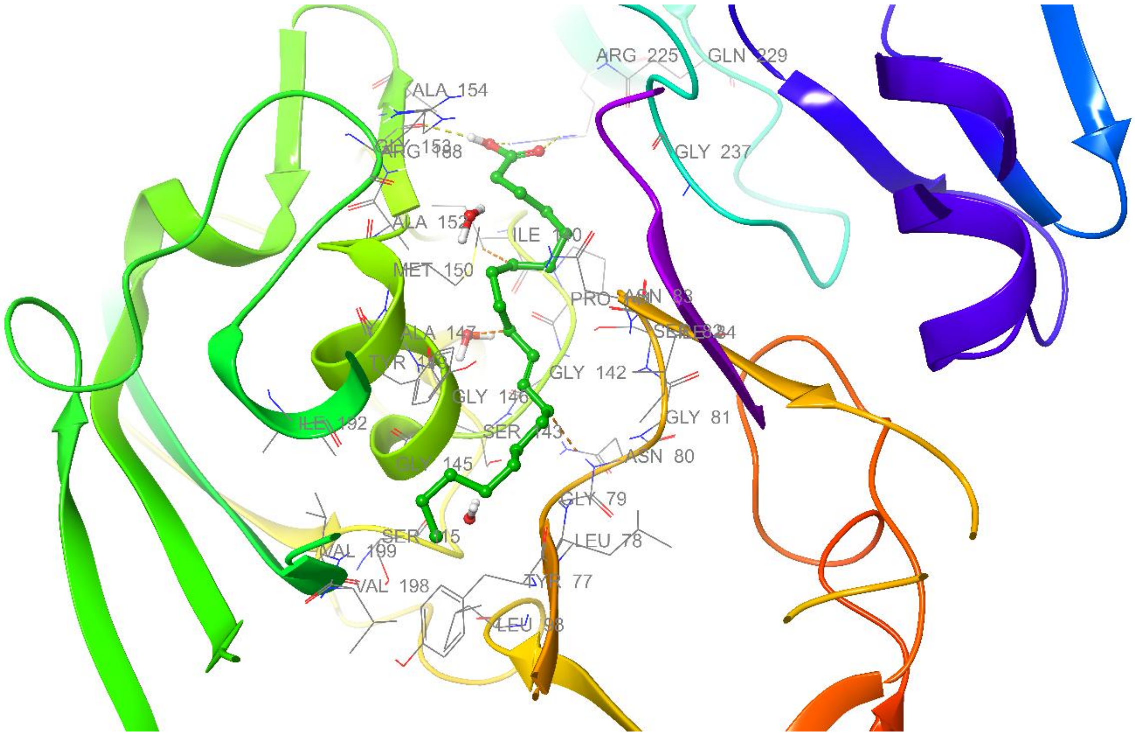

Figure 28S. 3D interaction diagram with 1HSK for **14**.

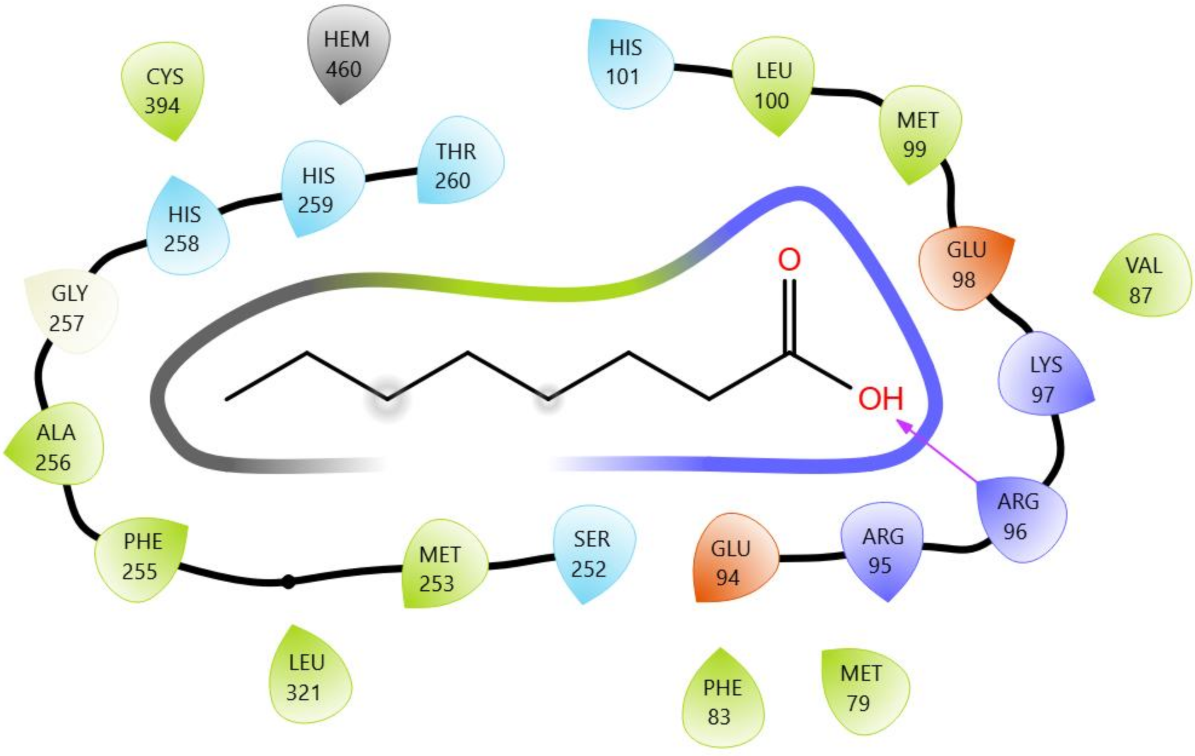

Figure 29S. 2D interaction diagram with 1EA1 for **1**.

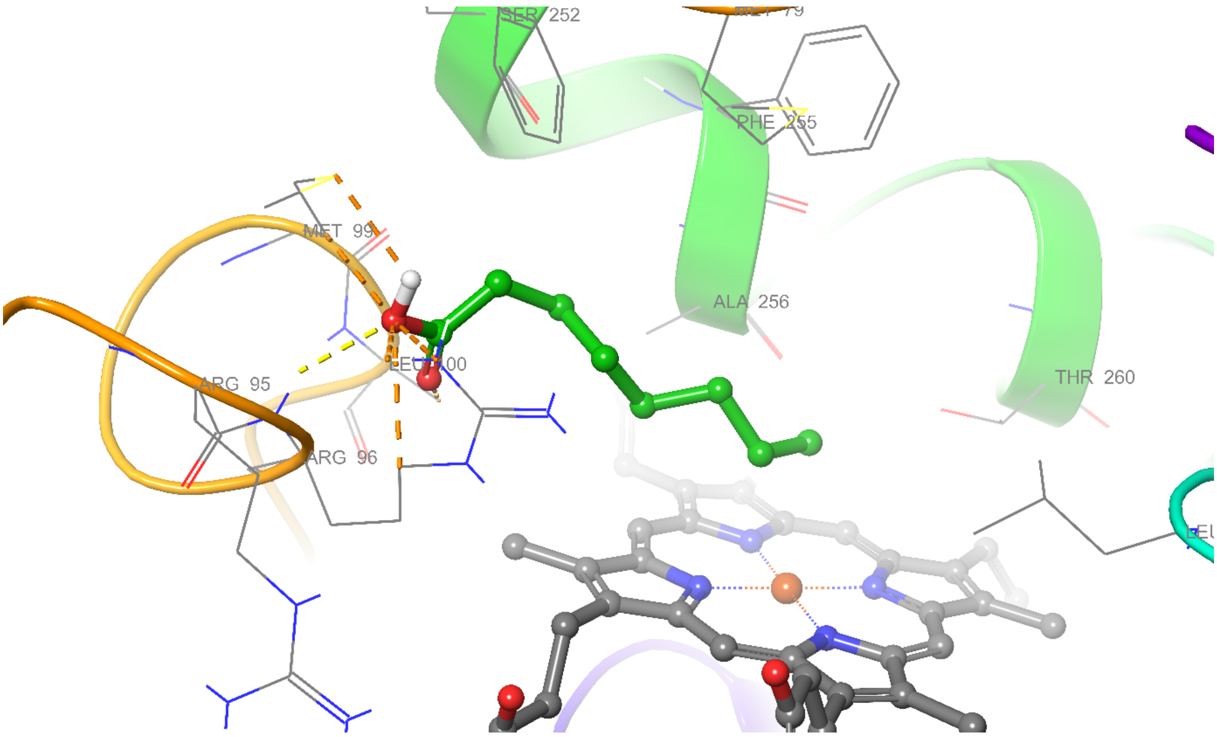

Figure 30S. 3D interaction diagram with 1EA1 for **1**.

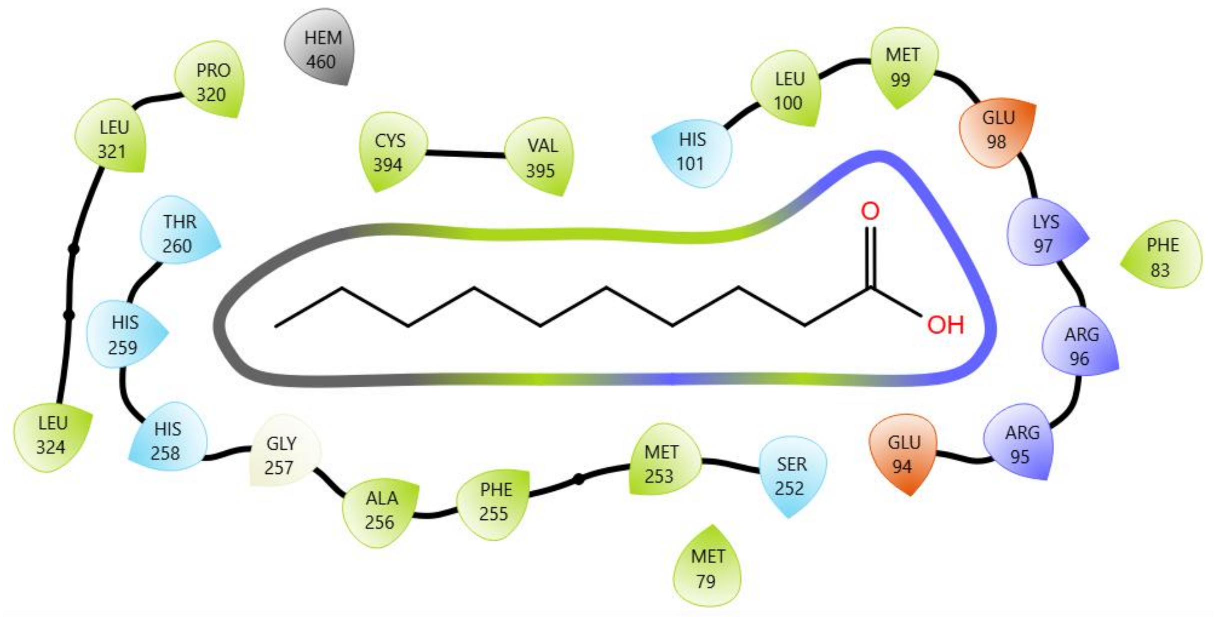

Figure 31S. 2D interaction diagram with 1EA1 for **2**.

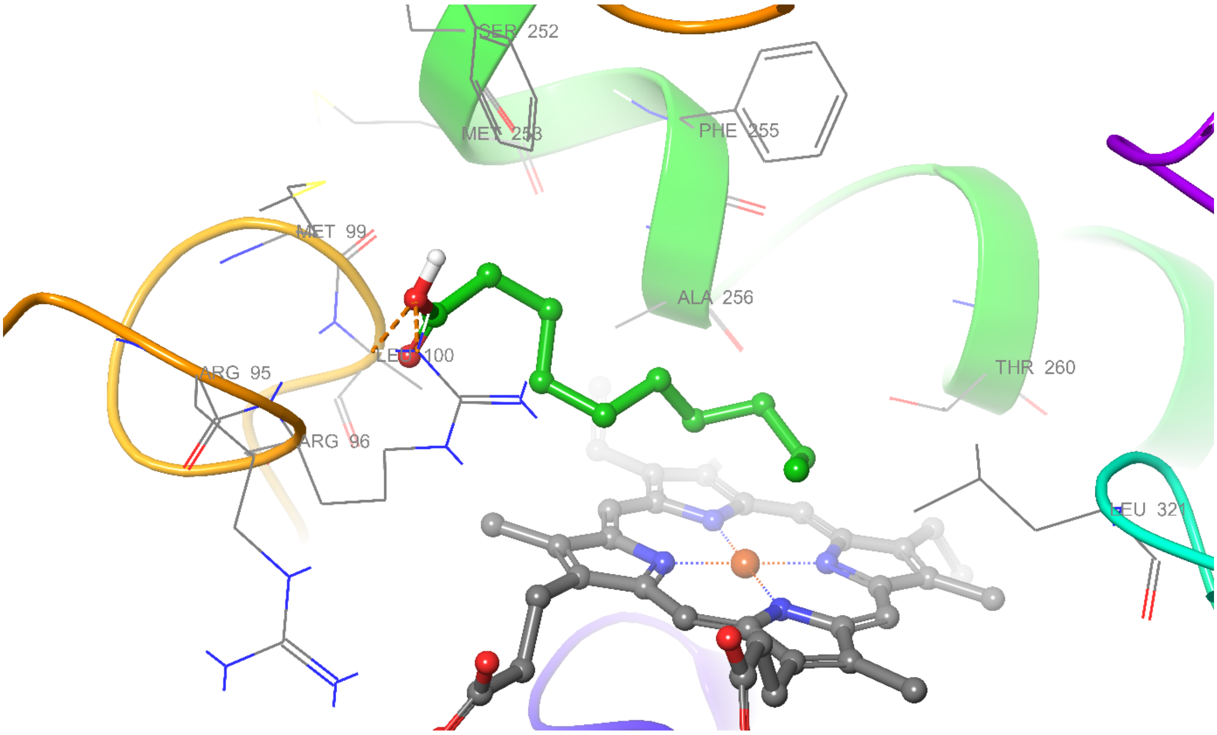

Figure 32S. 3D interaction diagram with 1EA1 for **2**.

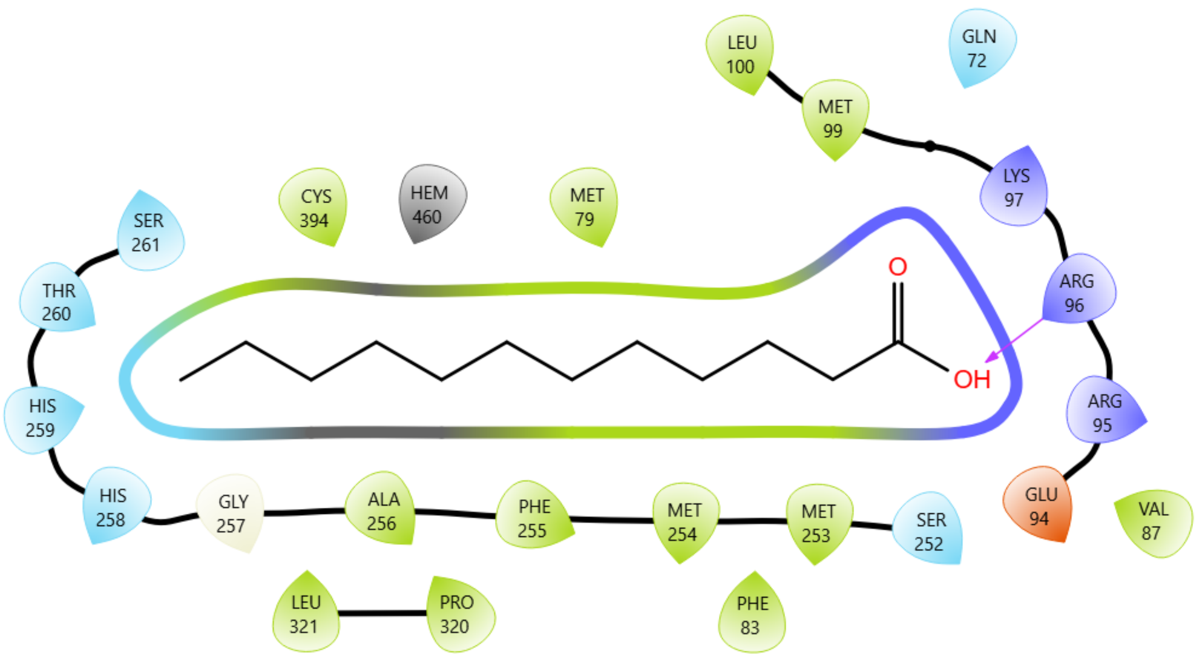

Figure 33S. 2D interaction diagram with 1EA1 for **3**.

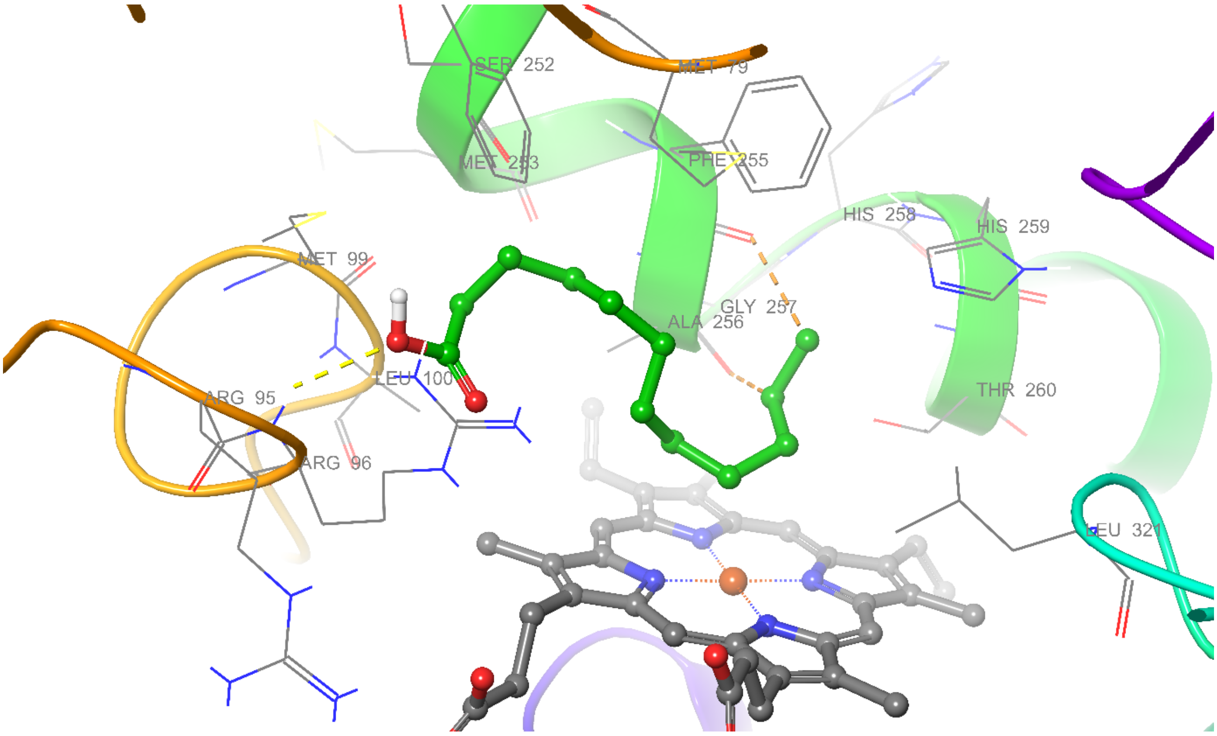

Figure 34S. 3D interaction diagram with 1EA1 for **3**.

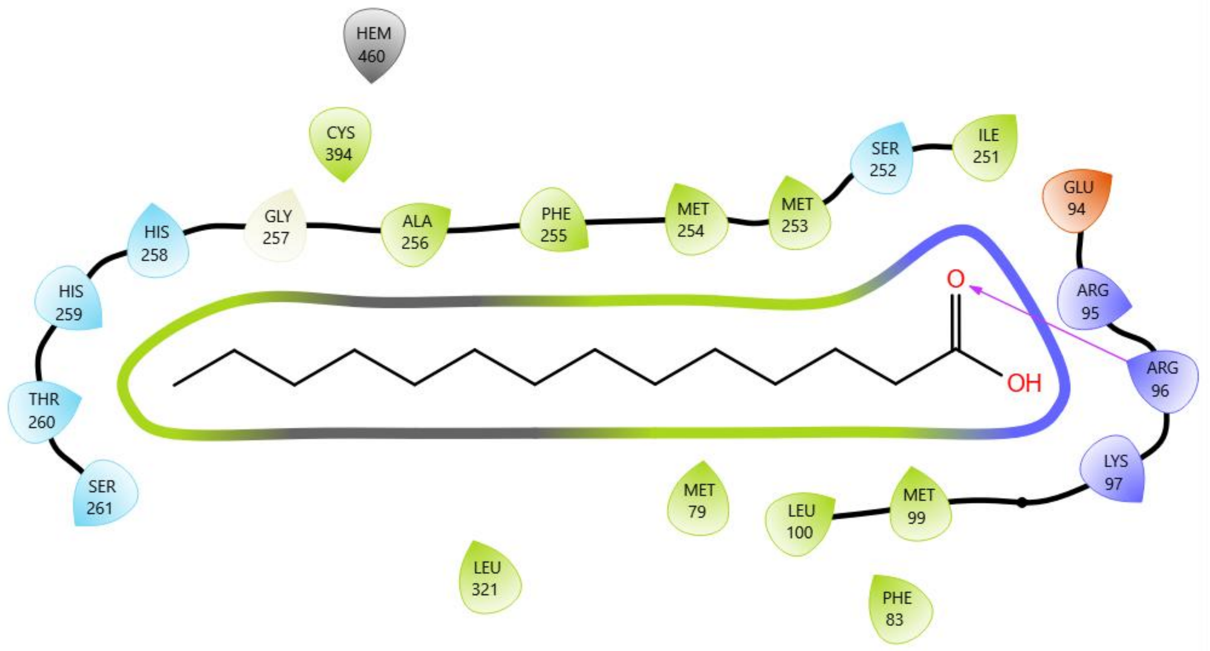

Figure 35S. 2D interaction diagram with 1EA1 for **4**.

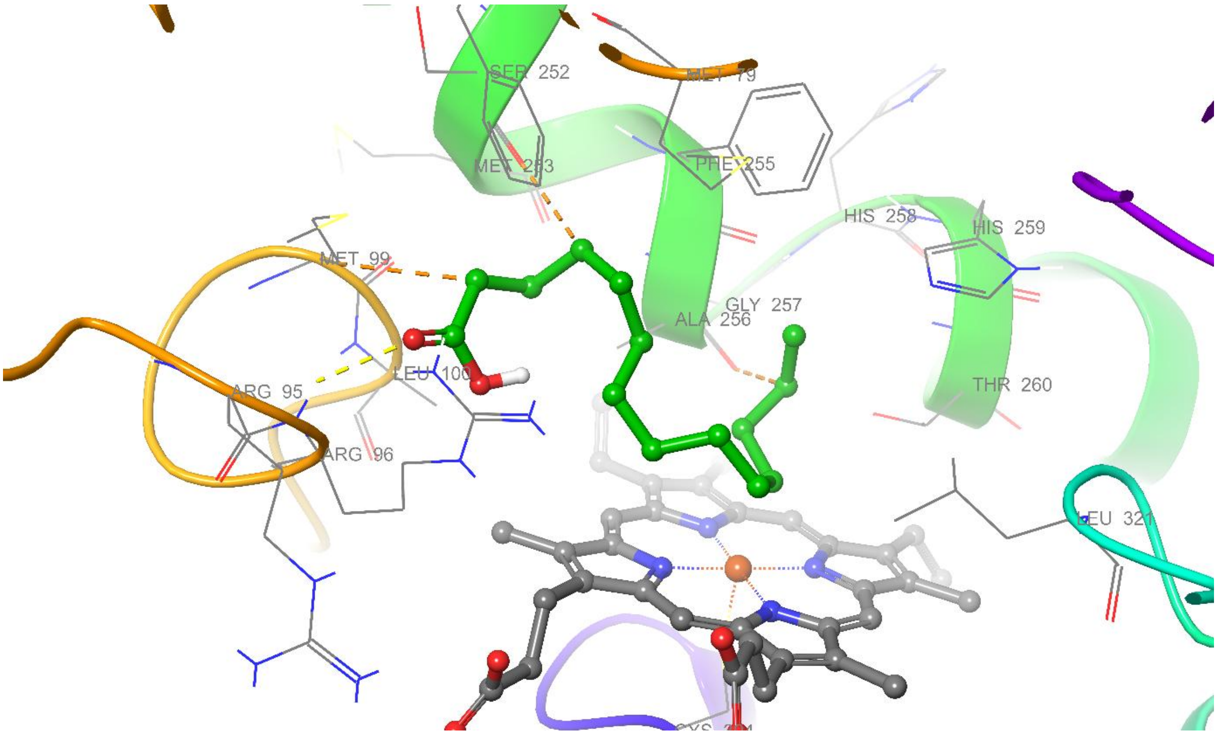

Figure 36S. 3D interaction diagram with 1EA1 for **4**.

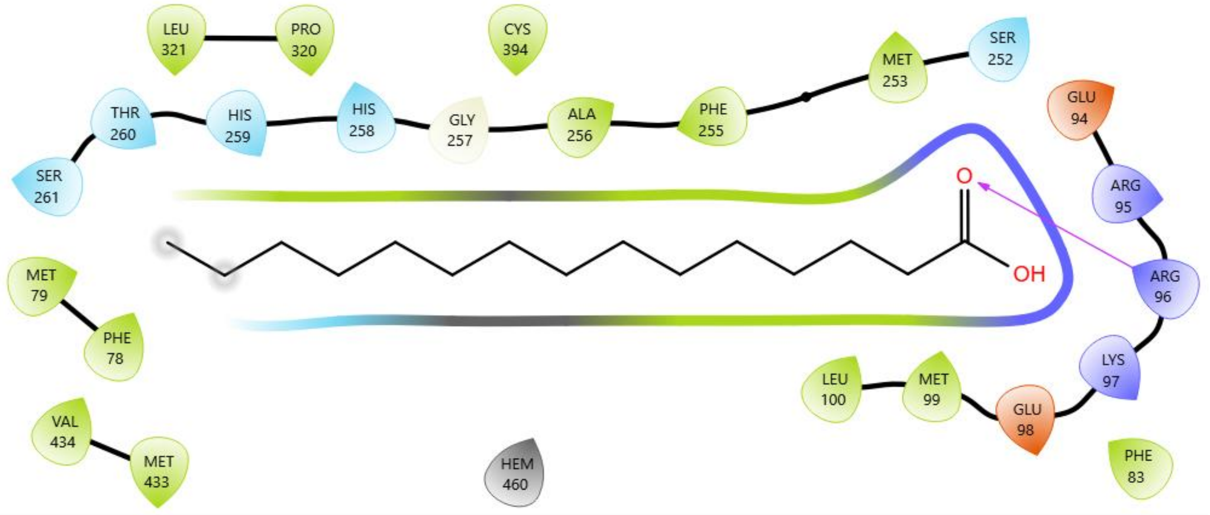

Figure 37S. 2D interaction diagram with 1EA1 for **5**.

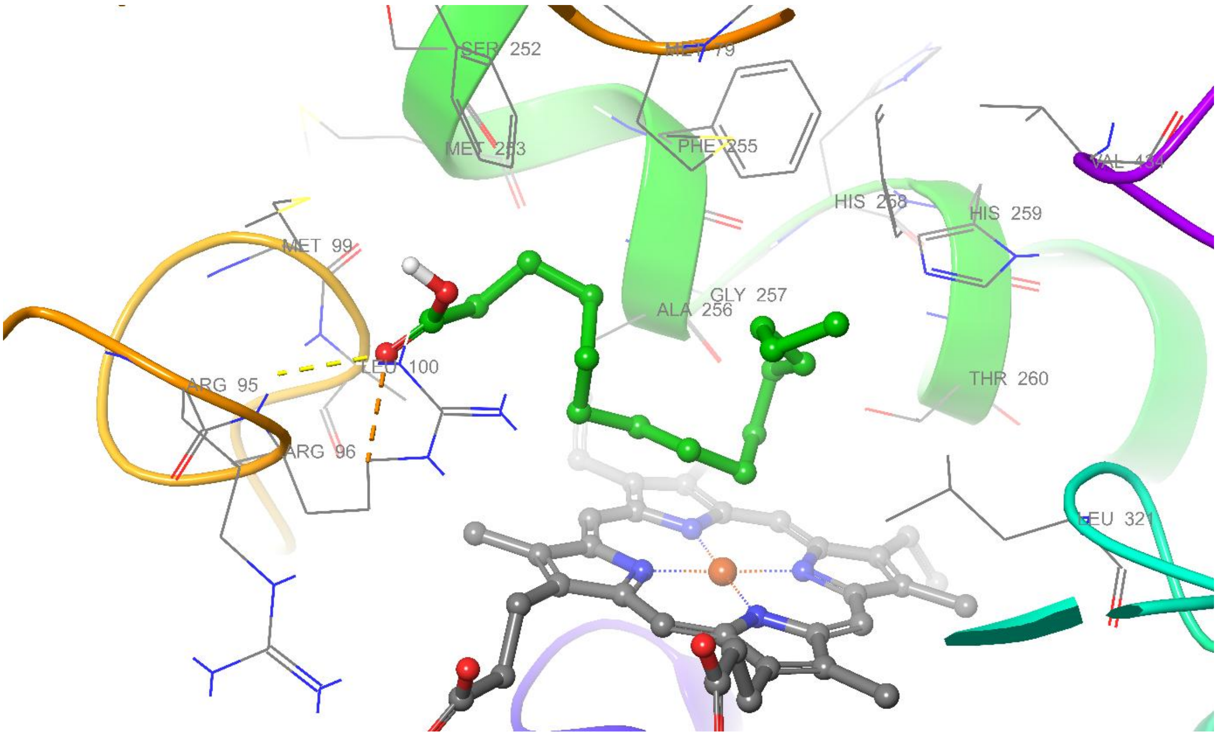

Figure 38S. 3D interaction diagram with 1EA1 for **5**.

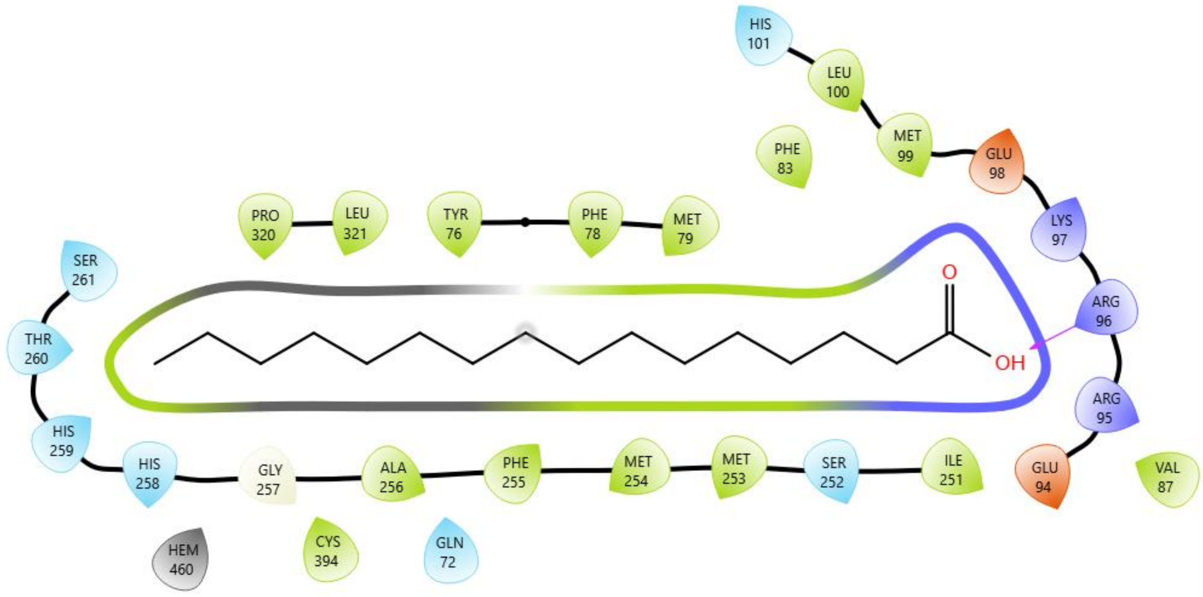

Figure 39S. 2D interaction diagram with 1EA1 for **6**.

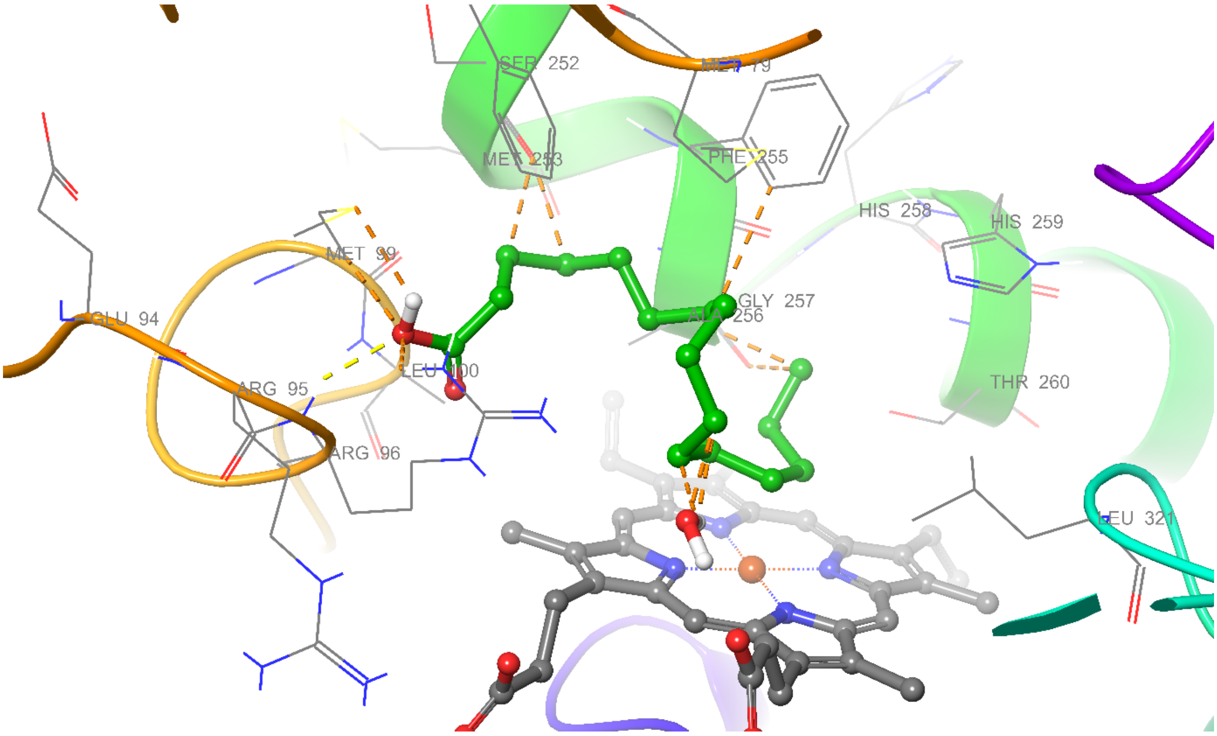

Figure 40S. 3D interaction diagram with 1EA1 for **6**.

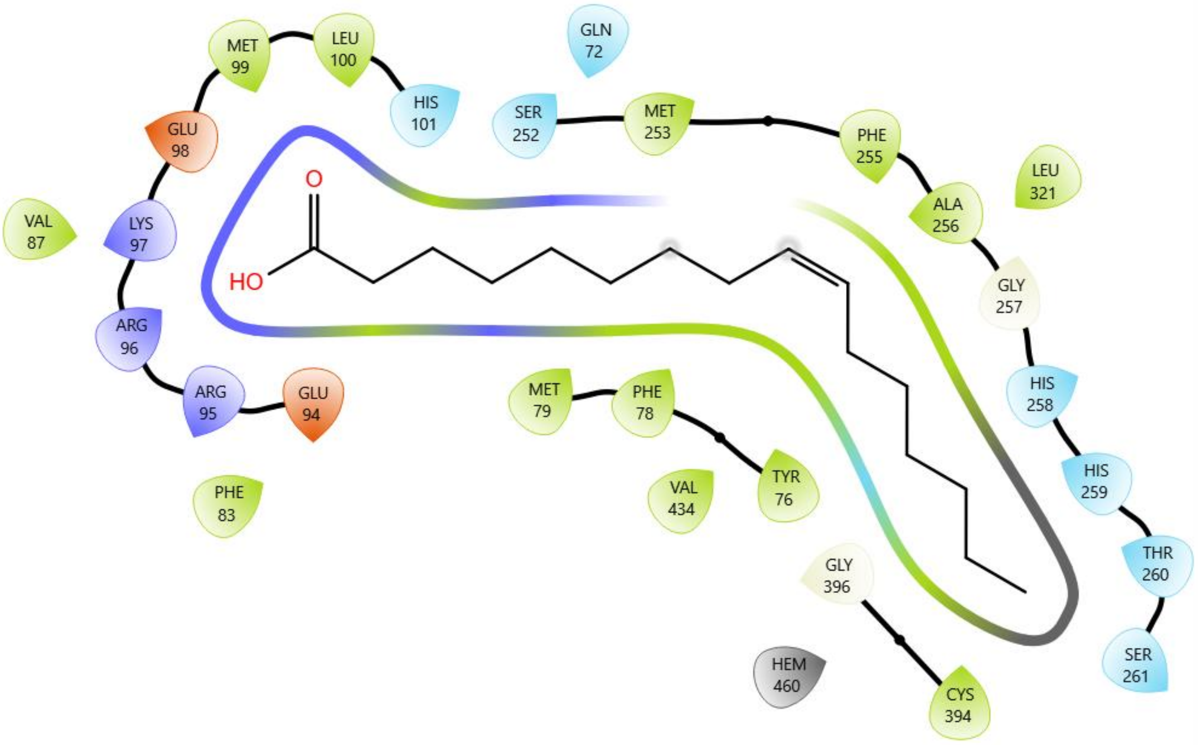

Figure 41S. 2D interaction diagram with 1EA1 for **7**.

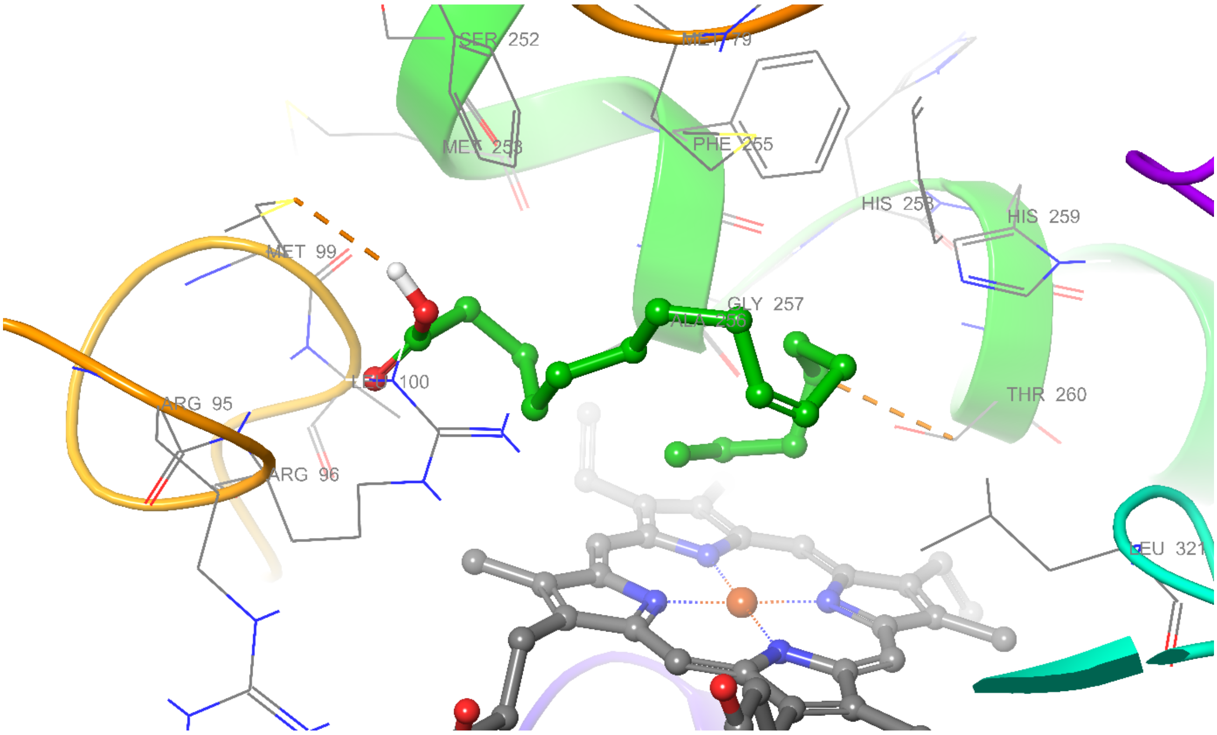

Figure 42S. 3D interaction diagram with 1EA1 for **7**.

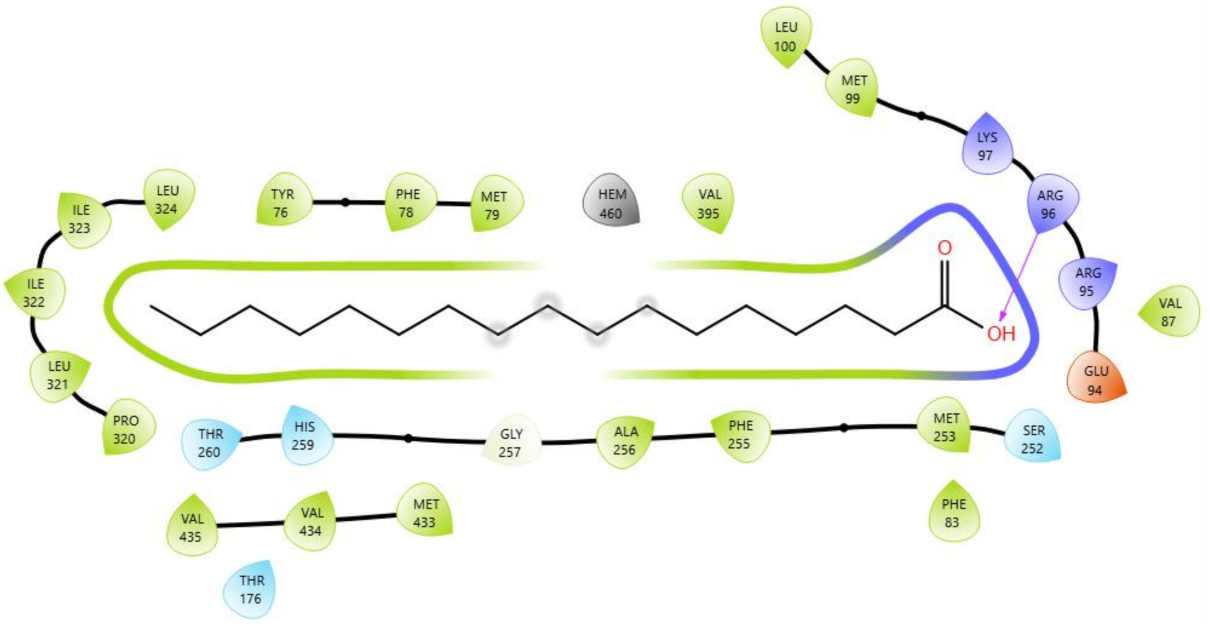

Figure 43S. 2D interaction diagram with 1EA1 for **8**.

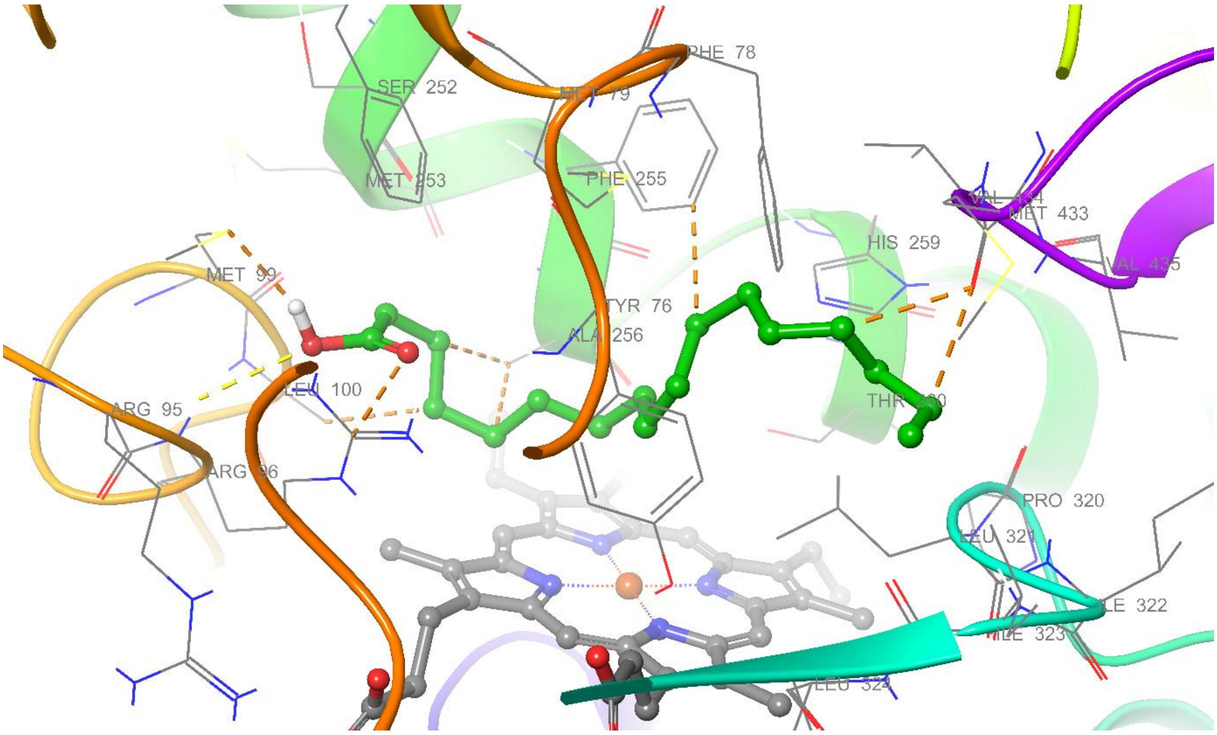

Figure 44S. 3D interaction diagram with 1EA1 for **8**.

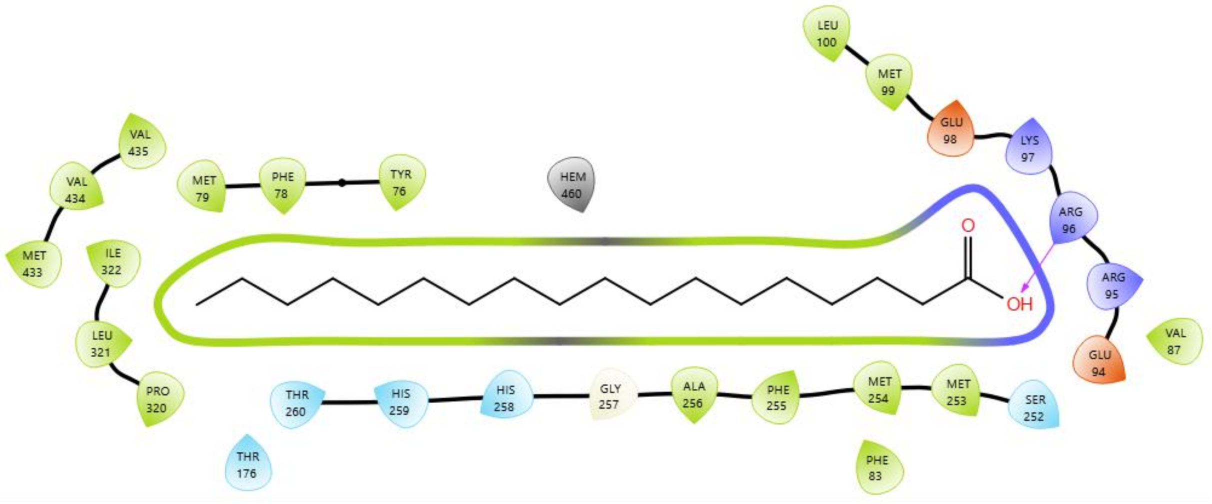

Figure 45S. 2D interaction diagram with 1EA1 for **9**.

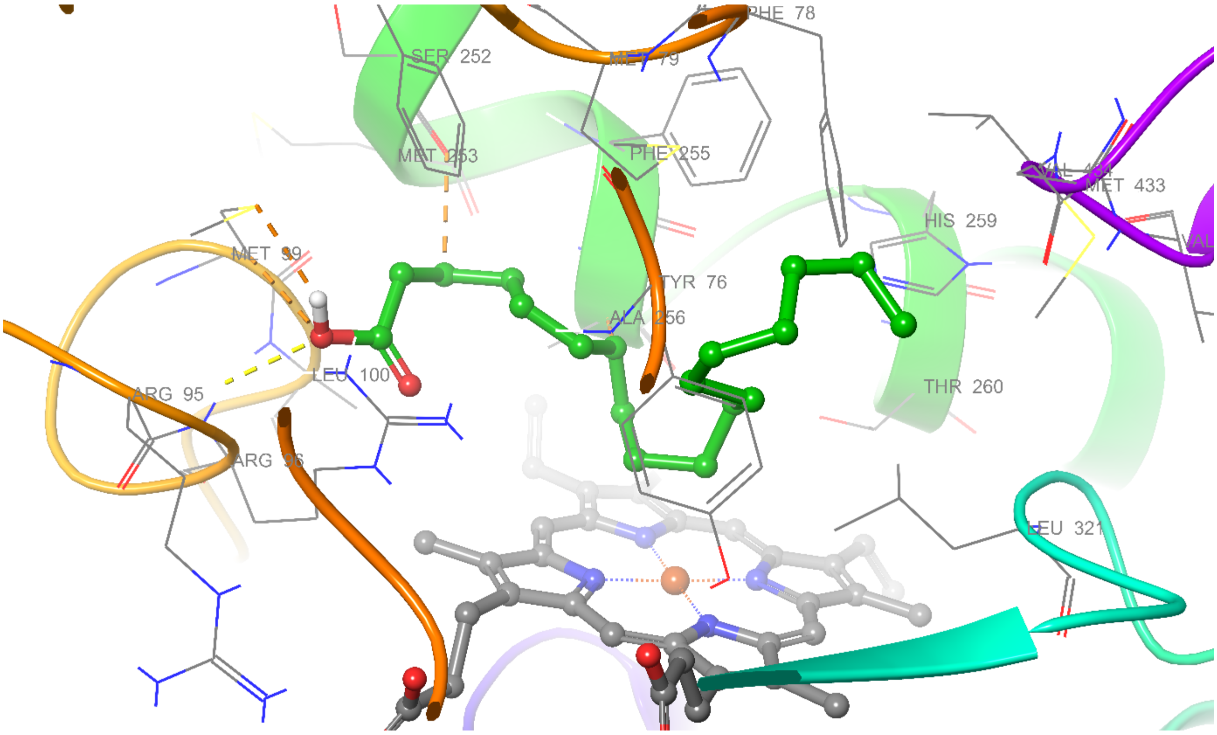

Figure 46S. 3D interaction diagram with 1EA1 for **9**.

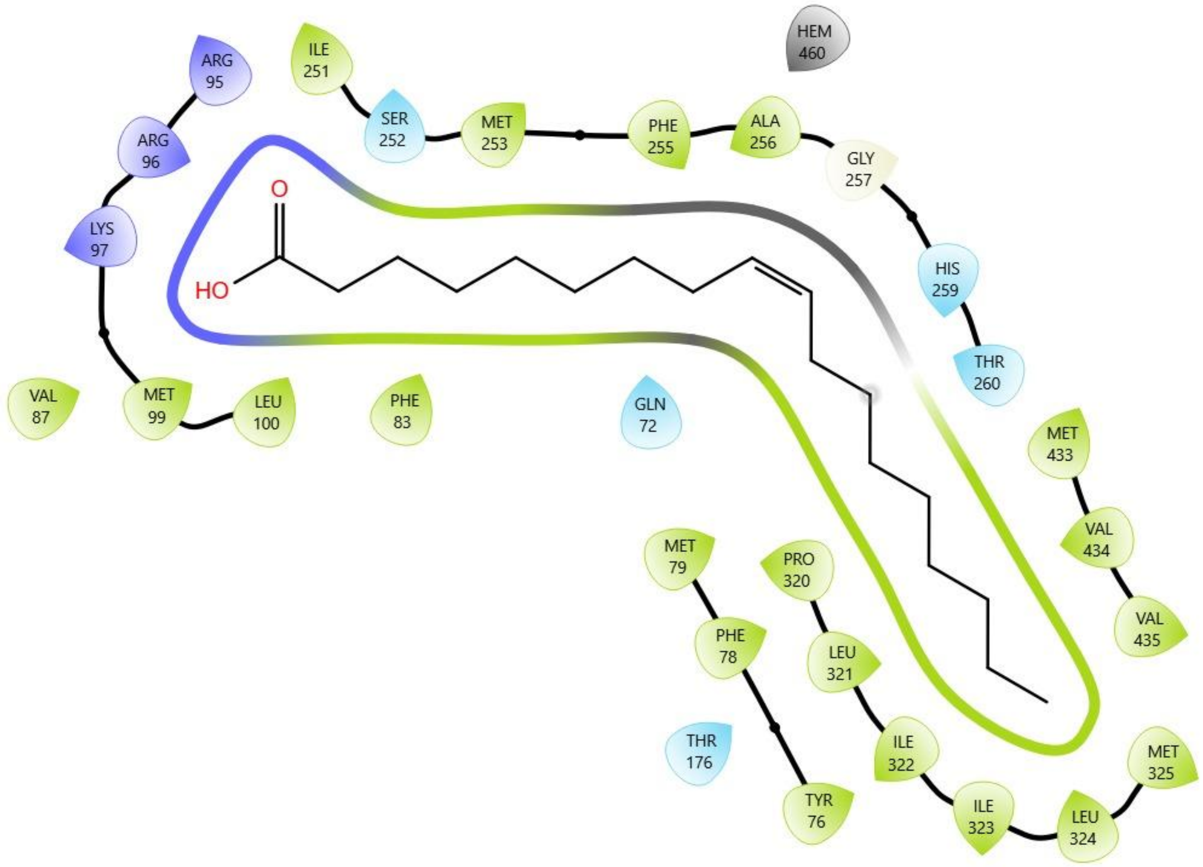

Figure 47S. 2D interaction diagram with 1EA1 for **10**.

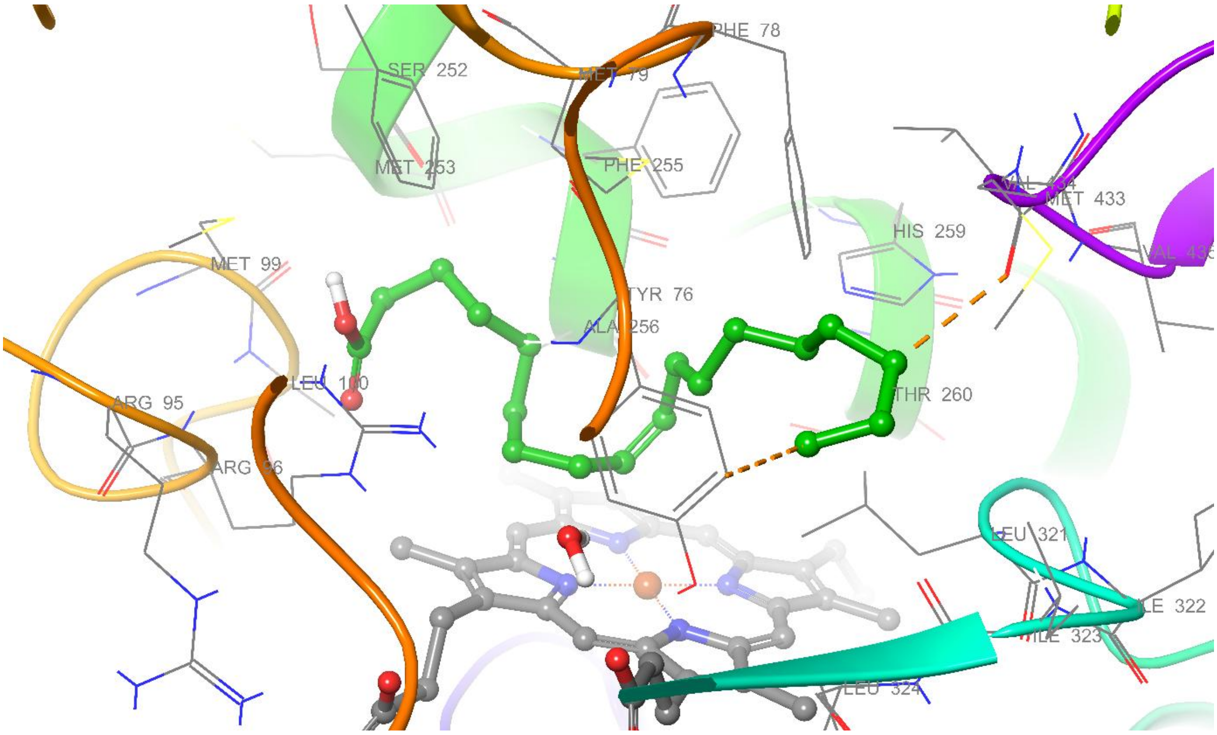

Figure 48S. 3D interaction diagram with 1EA1 for **10**.

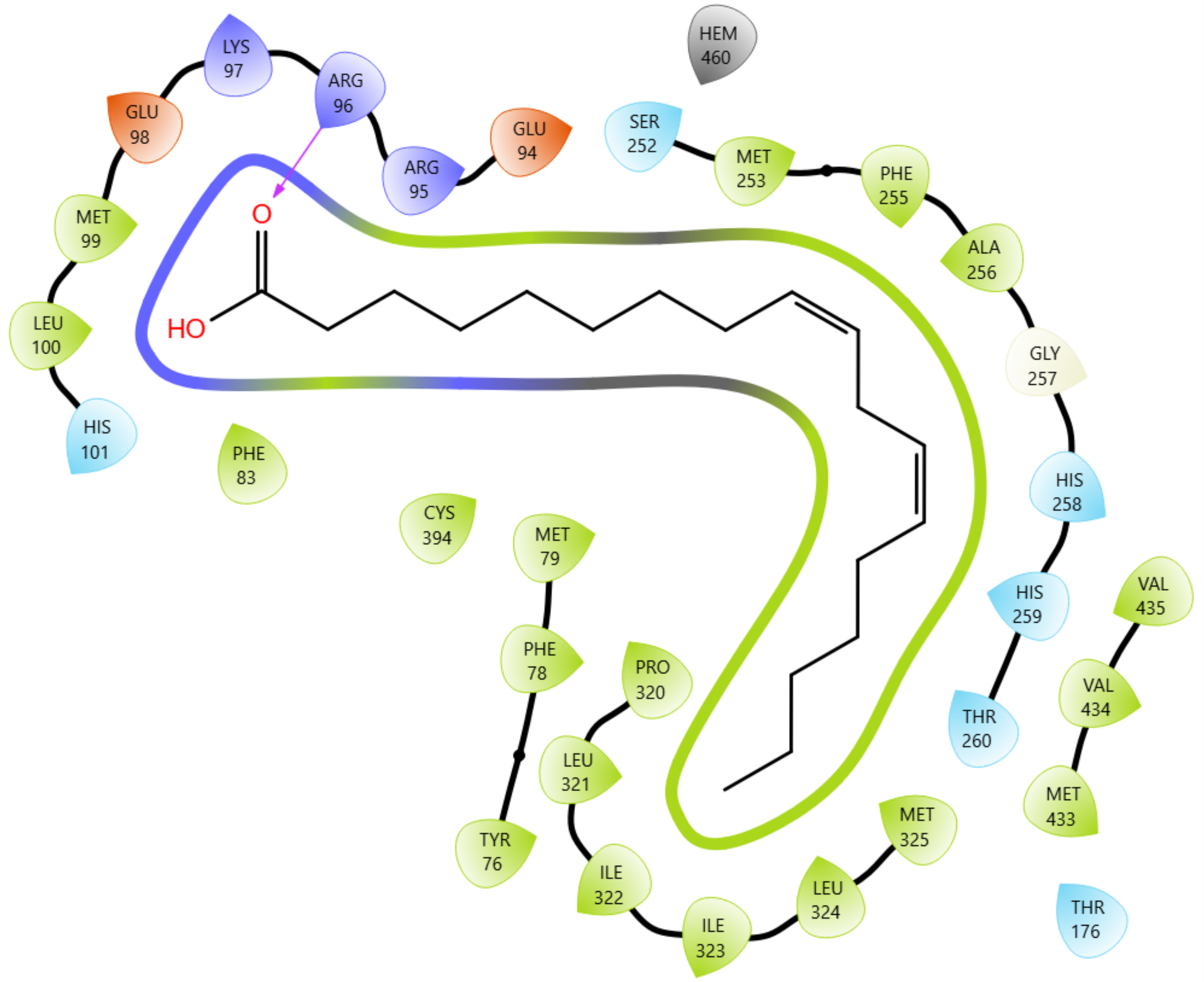

Figure 49S. 2D interaction diagram with 1EA1 for **11**.

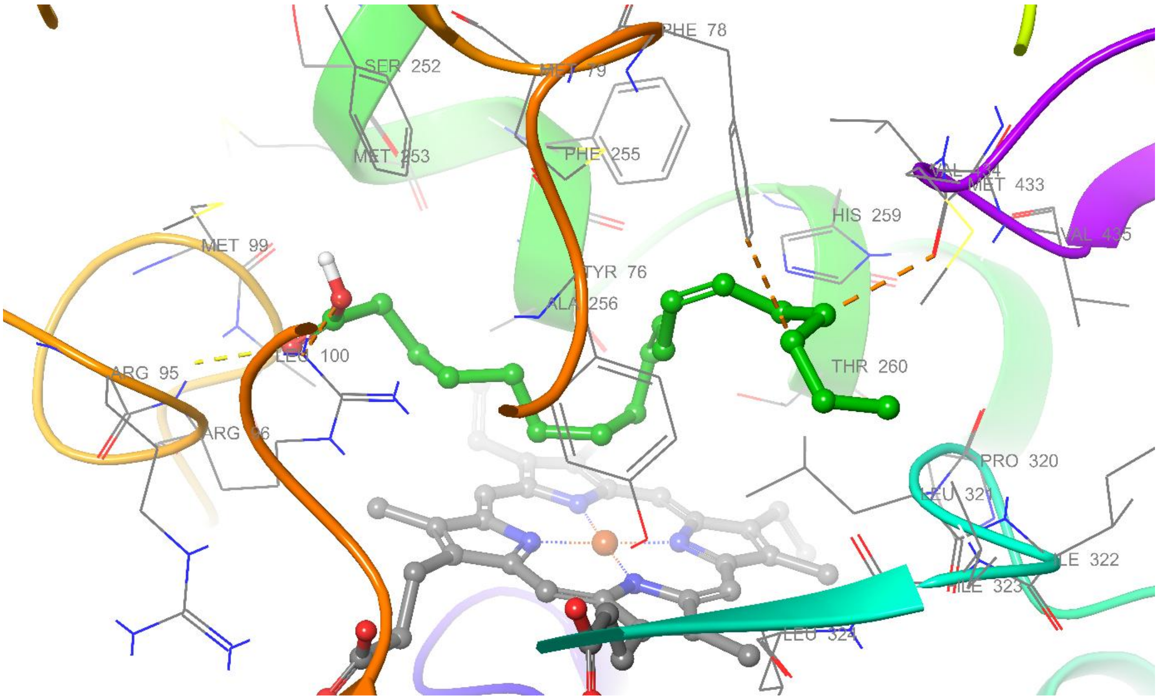

Figure 50S. 3D interaction diagram with 1EA1 for **11**.

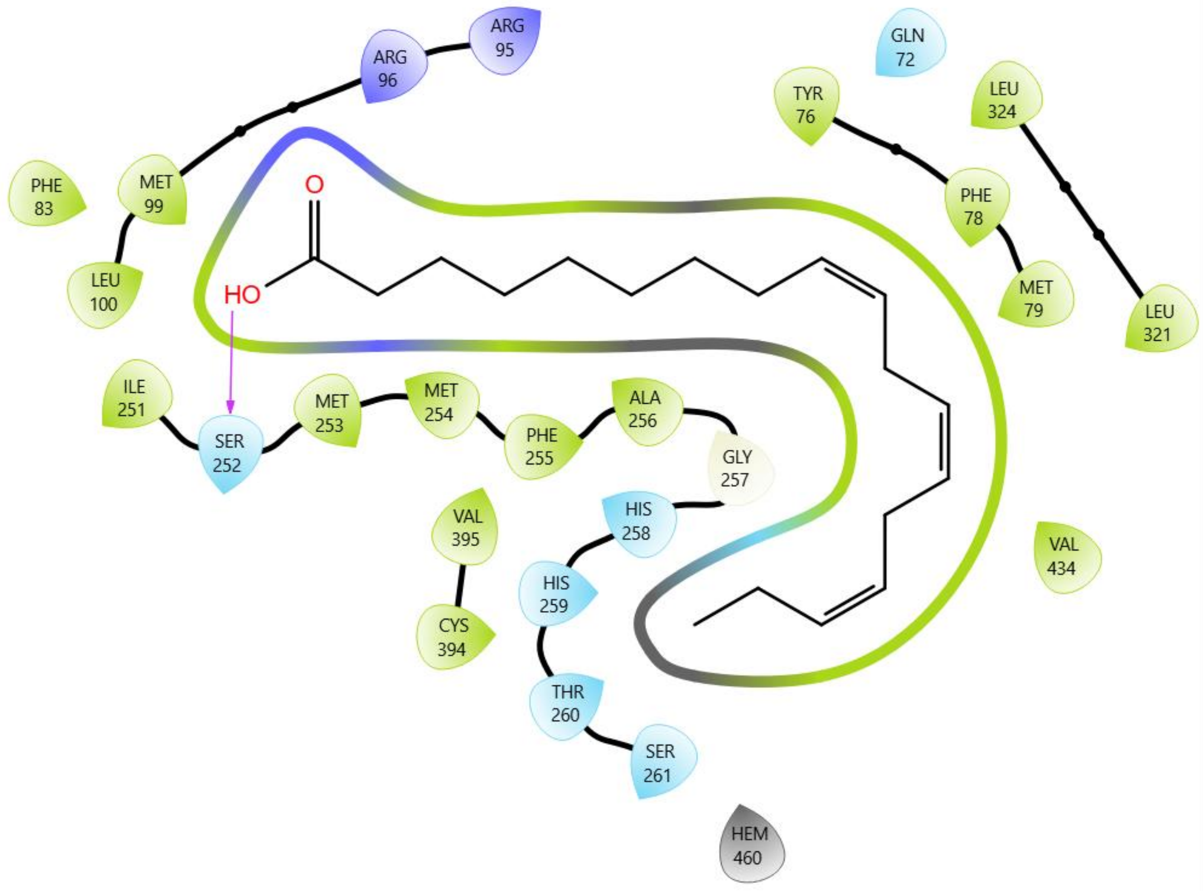

Figure 51S. 2D interaction diagram with 1EA1 for **12**.

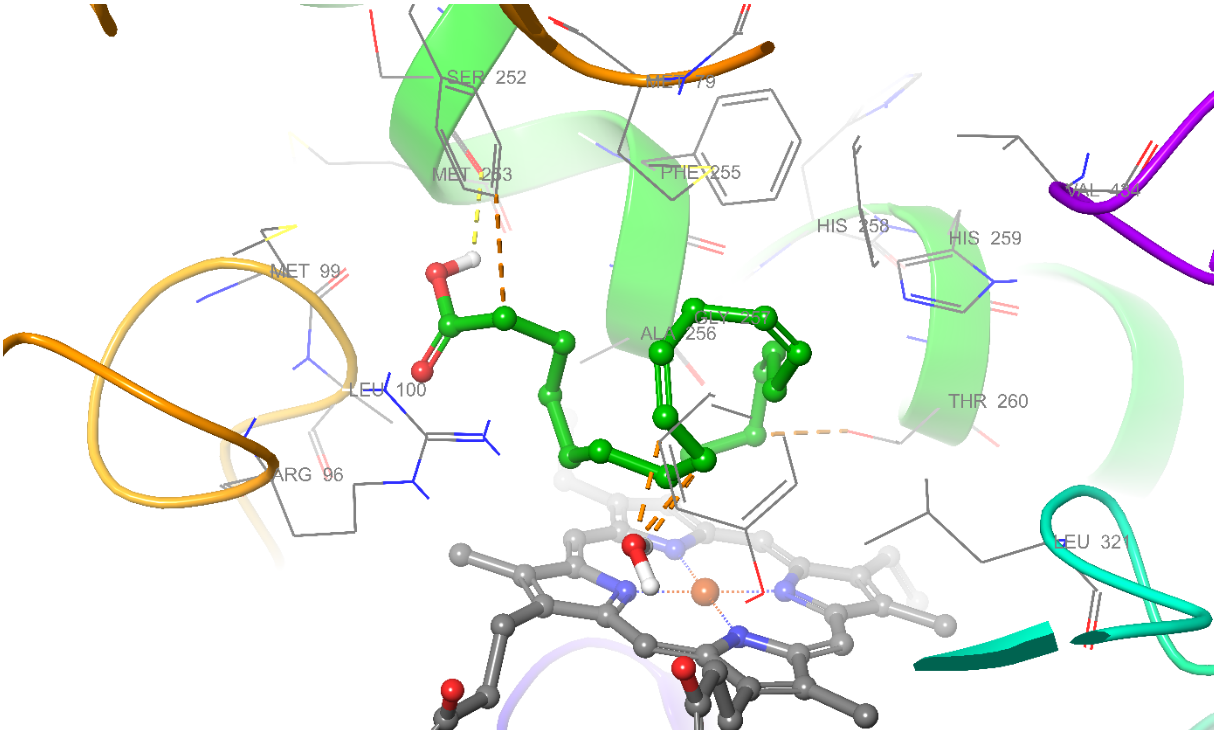

Figure 52S. 3D interaction diagram with 1EA1 for **12**.

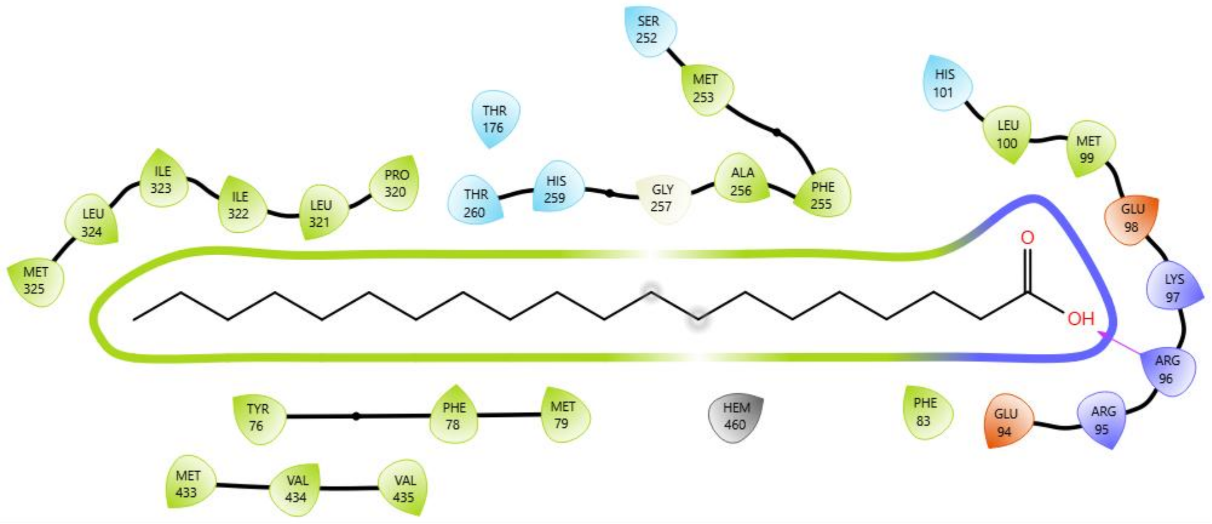

Figure 53S. 2D interaction diagram with 1EA1 for **13**.

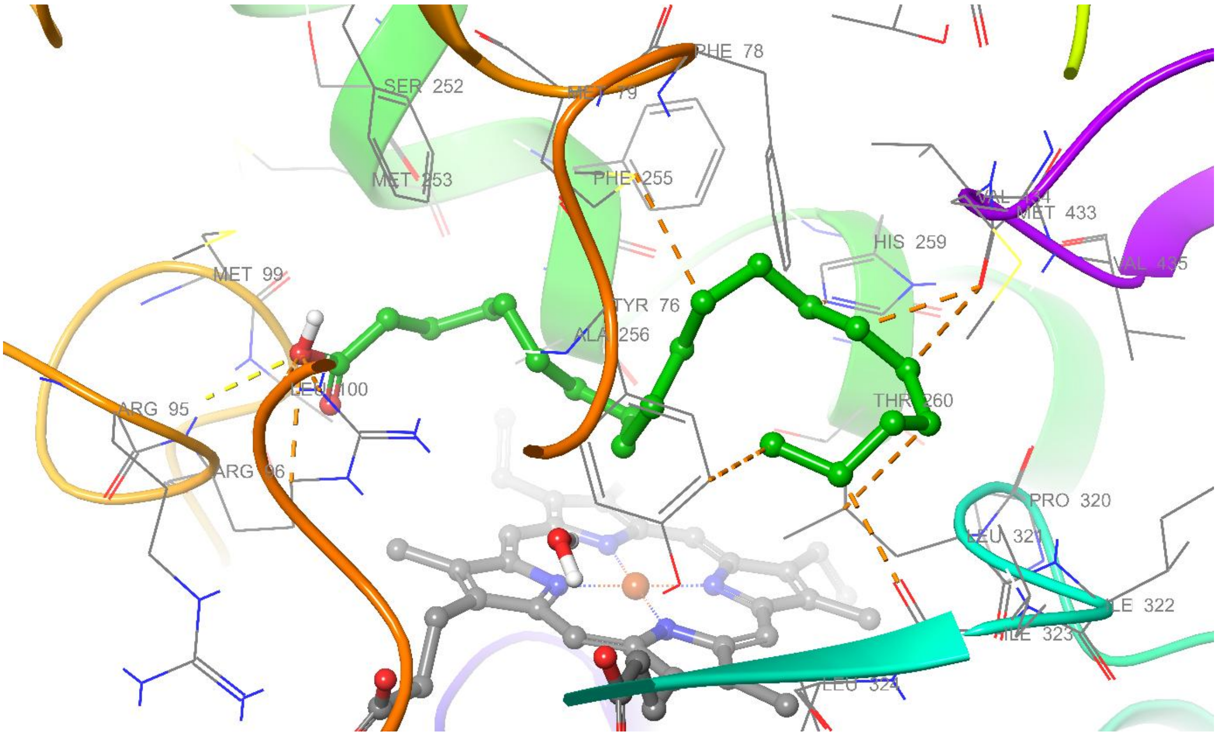

Figure 54S. 3D interaction diagram with 1EA1 for **13**.

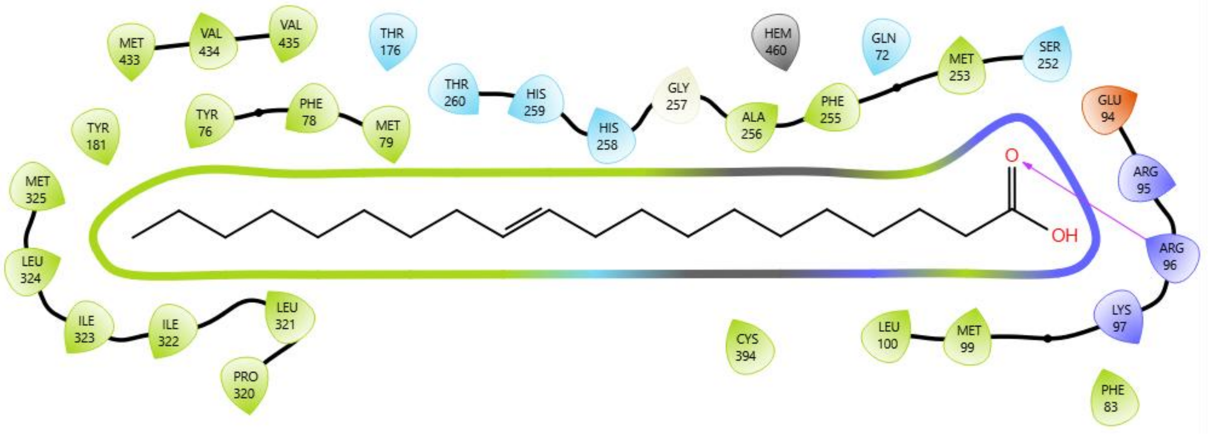

Figure 55S. 2D interaction diagram with 1EA1 for **14**.

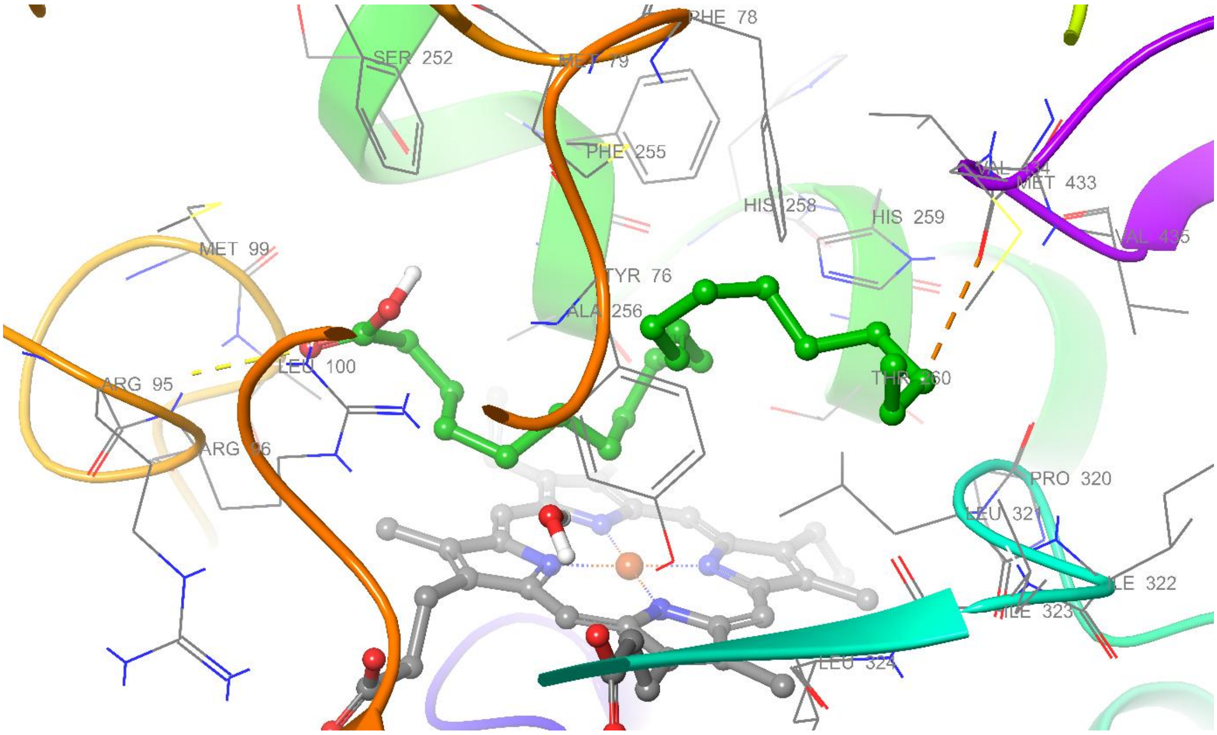

Figure 56S. 3D interaction diagram with 1EA1 for **14**.
